# Morphological evolution and modularity of the caecilian skull

**DOI:** 10.1186/s12862-018-1342-7

**Published:** 2019-01-22

**Authors:** Carla Bardua, Mark Wilkinson, David J. Gower, Emma Sherratt, Anjali Goswami

**Affiliations:** 1 Department of Life Sciences, Natural History Museum, London, UK; 2 Department of Genetics, Evolution and Environment, UCL, London, UK; 3 School of Biological Sciences, University of Adelaide, Adelaide, Australia

**Keywords:** Amphibia, Caecilians, Cranial, Evolution, Evolutionary rate, Gymnophiona, Integration, Macroevolution, Modularity, Skulls

## Abstract

**Background:**

Caecilians (Gymnophiona) are the least speciose extant lissamphibian order, yet living forms capture approximately 250 million years of evolution since their earliest divergences. This long history is reflected in the broad range of skull morphologies exhibited by this largely fossorial, but developmentally diverse, clade. However, this diversity of form makes quantification of caecilian cranial morphology challenging, with highly variable presence or absence of many structures. Consequently, few studies have examined morphological evolution across caecilians. This extensive variation also raises the question of degree of conservation of cranial modules (semi-autonomous subsets of highly-integrated traits) within this clade, allowing us to assess the importance of modular organisation in shaping morphological evolution. We used an intensive surface geometric morphometric approach to quantify cranial morphological variation across all 32 extant caecilian genera. We defined 16 cranial regions using 53 landmarks and 687 curve and 729 surface sliding semilandmarks. With these unprecedented high-dimensional data, we analysed cranial shape and modularity across caecilians assessing phylogenetic, allometric and ecological influences on cranial evolution, as well as investigating the relationships among integration, evolutionary rate, and morphological disparity.

**Results:**

We found highest support for a ten-module model, with greater integration of the posterior skull. Phylogenetic signal was significant (*K*_mult_ = 0.87, *p* < 0.01), but stronger in anterior modules, while allometric influences were also significant (*R*^*2*^ = 0.16, *p *< 0.01), but stronger posteriorly. Reproductive strategy and degree of fossoriality were small but significant influences on cranial morphology (*R*^*2*^ = 0.03–0.05), after phylogenetic (*p* < 0.03) and multiple-test (*p* < 0.05) corrections. The quadrate-squamosal ‘cheek’ module was the fastest evolving module, perhaps due to its pivotal role in the unique dual jaw-closing mechanism of caecilians. Highly integrated modules exhibited both high and low disparities, and no relationship was evident between integration and evolutionary rate.

**Conclusions:**

Our high-dimensional approach robustly characterises caecilian cranial evolution and demonstrates that caecilian crania are highly modular and that cranial modules are shaped by differential phylogenetic, allometric, and ecological effects. More broadly, and in contrast to recent studies, this work suggests that there is no simple relationship between integration and evolutionary rate or disparity.

**Electronic supplementary material:**

The online version of this article (10.1186/s12862-018-1342-7) contains supplementary material, which is available to authorized users.

## Background

A thorough understanding of the morphological evolution of a clade requires considering both intrinsic (e.g., developmental) and extrinsic (e.g., environmental) influences. Examining morphological evolution through clade history can reveal disparate patterns, from phylogenetic conservatism (e.g., [1, 2]) to repeated convergent evolution through adaptation (e.g., [3–6]) or directional evolution [7, 8]. Quantification of these patterns also often further demonstrates that different biological structures, or different parts of structures, may deviate in their patterns of evolution. Individual structures may have divergent localised functions or different developmental origins and therefore be subject to different constraints. However, each structure also contributes to the functionality and, ultimately, to the fitness of the whole organism. For example, multiple levels of functional and developmental interactions have been demonstrated within Felidae, from the level of the individual vertebrae [9], to different vertebral regions [10], to the level of the presacral vertebral column [11]. This hierarchy of interactions across the presacral vertebral column of felids demonstrates how multiple levels of organisation shape the morphological evolution of a complex structure.

The complex hierarchy of functional, developmental or genetical relationships among traits underlies the concepts of modularity and integration. Integration refers to the covariation or correlation amongst traits, while modularity refers to the partitioning of highly integrated traits into semi-independent subsets (modules). Modular structures can be identified as those that can be divided into subunits, or modules, that exhibit strong within-module integration (trait covariation) and weaker between-module integration [12–14]. Trait regionalisation in modular networks is hypothesized to promote evolvability [15], allowing strongly related traits to covary, and coevolve, with relative autonomy from other regions. Strong integration within modules has been found to facilitate (e.g., [9, 16, 17]), constrain (e.g., [18, 19]) or both facilitate and constrain [20] evolution, affecting the magnitude and direction of an organismal lineage’s response to selection [20–23]. More specifically, integration directs evolutionary shifts to favoured regions of morphospace, i.e. along paths of least resistance, which may promote homoplasy as organismal evolution is directed along similar evolutionary trajectories defined by the underlying architecture of trait interactions [20, 24, 25]. Identifying the structure of these trait interactions through quantitative analysis is thus central to advancing understanding of morphological evolution.

The focus of many studies of phenotypic integration and modularity is the vertebrate cranium, a complex structure with multiple layers of functional and developmental patterning [26]. Housing many important parts of the sensory, feeding, respiratory, and communication systems, the cranium has been shaped by numerous, often competing, demands. The cranium is also developmentally complex, with different embryonic origins (neural crest and paraxial mesoderm) and types of ossifications (endochondral and intramembranous) across the cranial bones. Previous studies have identified a complex modular cranial structure in some vertebrate clades, reflecting this functional and developmental complexity. A six-module model has been identified in carnivorans [18], macaques [27, 28], and across therians [29], suggesting this model may be conserved across a range of mammals. Modularity has also been studied across 15 orders of Mammalia [22], and similar patterns of trait correlations were found, suggesting a common covariance structure of the mammalian cranium. Avian crania have been proposed to have seven distinct modules [19], with some concordance with mammalian modules (vault, basicranium) as well as some novel modules (e.g., nares). Several studies have limited analysis to a higher level division of the neurocranium and facial modules, and this model also has strong support [30, 31]. Previous work on cranial modularity has been strongly biased towards amniotes (especially mammals). In contrast, studies of modularity and integration in non-amniotes (amphibians) are rare, limiting our understanding of the diversity of patterns of modularity and our ability to reconstruct evolutionary trends in those clades.

As the only extant non-amniote tetrapod clade, Lissamphibia (Anura, Caudata and Gymnophiona = frogs and toads, salamanders and newts, and caecilians, respectively) represents a unique lineage for comparison with patterns of modularity and integration identified in amniote crania. Evolutionary modularity has been explored in salamander crania using cranial ossification sequences, and support was found for two (or four) developmental modules in terms of coordinated timing of development [32]. However, landmark studies suggest salamander crania may be highly integrated, with no distinct modules across the cranium of the alpine newt [33] or the crania of species of *Triturus* [34]. Modularity has also been studied in frogs, by comparing morphological evolution and covariation in limbs and crania across the Myobatrachidae [1], testing alternative two or three-module models, but finding no strong support for any modular structure. Similarly, only weak support was found for models of three to five developmental, functional and hormonal modules [35] in crania of the anuran *Rhinella granulosa* complex. In a previous investigation, Sherratt [36] found support for a two-module model in caecilian crania, with independence of the snout relative to the rest of the cranium. While these studies have laid the groundwork for the study of modularity in Lissamphibia, advances in data capture and analytical approaches allow us to expand this work with high dimensional data that better represent the diversity of cranial morphology and directly compare support for a broad range of alternative patterns of modularity, as in recent studies of mammalian (e.g., [28]) and avian [19] crania.

Caecilians are the least studied and least speciose major clade (order) of Lissamphibia with, as of writing, 208 currently recognised species classified within 32 genera and ten families [37–39]. Elongate and limbless, ranging in adult size from approximately 60 mm (e.g., *Idiocranium russelli*: [40]) to over 1700 mm (*Caecilia thompsoni*: [41]), caecilians may superficially resemble snakes or earthworms. Fertilisation is internal, and caecilians are the only amphibians (with the exception of the tailed frog *Ascaphus truei*) where males have a copulatory organ [42]. Both viviparity and oviparity are evident in this clade [43] and precocial feeding (on maternal oviducts, or skin, or their secretions) using specialised, vernal dentitions occurs in at least some viviparous species [44, 45] and in some direct developing oviparous species [46], respectively. Caecilians possess two sets of jaw-closing muscles [47], a mechanism unique among vertebrates. The jaw joint is kinetic [48], which may enhance bite force [49].

Caecilians are predominantly found in tropical subsurface habitats, spending most of their time as adults in leaf litter and moist soil (e.g., [50]). Secondarily aquatic caecilians are found in freshwater systems of the neotropics and are restricted to the family Typhlonectidae (e.g., [51]). Head first burrowing has long been thought to influence cranial morphology (e.g., [47, 52–54]) because many caecilians exhibit stegokrotaphic (closed), approximately conical crania with a subterminal mouth (see [54, 55] but also, [56]). Additional aspects of being endogeic (living in soil), are also thought to influence caecilian cranial morphology, for example by restricting gape size and perhaps promoting rotational feeding [57]. Although insufficient observational data exist to confirm an absolute link between endogeicity and burrowing in caecilians, they are presumed to be highly correlated in this clade, because no caecilian has ever been reported (or found by us) using another organism’s burrow. Here we use the term fossorial to communicate both active burrowing and more generally living in soil. Miniaturisation has been documented in some caecilians (e.g., [58, 59]), although the extent to which this might be causally linked to fossoriality is unknown. An extensive study of cranial shape evolution across caecilians using 3D cranial landmarks across 141 species [60] showed that, following an early expansion of morphospace, caecilian cranial evolution was both divergent (clades occupying distinct areas of morphospace) and convergent (similarities attributed to dedicated fossoriality in distantly related taxa). Sherratt et al.’s [60] study represents the largest quantitative examination of morphological evolution across Gymnophiona to date and demonstrated the complexity of cranial evolution in caecilians, though cranial regions were not analysed separately, such that anatomically-localised patterns were not investigated.

Caecilian crania exhibit great variation in the number, size and position of cranial bones as a result of extensive and variable fusion of bones [55, 61]. Consequently, it is more difficult to quantify and compare cranial morphology across Gymnophiona compared to most other vertebrate orders, which largely have relatively conservative crania, in terms of element presence, such as mammals or birds. The highly variable nature of caecilian crania makes traditional landmarking approaches challenging, because this approach would not allow shape in all variably present or variably fused bones to be quantified. This is because all landmarks must be present across all specimens. Thus here we utilise a surface-based geometric morphometric approach, which allows inclusion of individual elements and grouped elements, with grouping of elements based on developmental and/or presumed functional relationships. Hence, variably present elements (e.g., mesethmoid) can be grouped with other adjacent, functionally or developmentally-related, and consistently present elements (e.g., frontal) so as not to exclude them from analyses. This method offers several benefits, including better representation of complex and variable structures and reduction in dependence on a limited number of homologous landmarks that would exclude much of the variation across caecilians. This approach has proven successful in recent studies of cranial morphology in mammals and birds (e.g., [16, 19, 62–64]). Using this approach, we quantify cranial morphological variation across Gymnophiona, sampling all extant genera. Our study also represents the first study to investigate a wide range of potentially modular structures within caecilian crania. We identify the best-supported model of cranial modularity and quantify morphological variation for each module. We test the relative strength of phylogenetic, allometric and ecological influences on morphology for each module. Finally, we investigate morphological diversity (disparity) and rates of morphological evolution for each module and across individual landmarks and semilandmarks, and test whether integration of traits facilitates or constrains evolution of the caecilian cranium.

## Results

### Modularity

Evaluating Modularity with Maximum Likelihood (EMMLi) analysis of the full trait correlation matrix identified the most parameterized model, the 16-module model, as the best fitting. However, as there is a tendency for analyses of densely sampled semilandmarks to favour highly parameterised models and because not all possible groupings of modules can be assessed in EMMLi analysis at present, we followed Felice and Goswami [19] and assessed the within- and between-region trait correlations for this model to determine whether any regions could be reasonably combined into larger modules. We merged regions into modules when the difference between the between-region correlation and the lowest within-region correlation was 0.2 or lower. We based this cut-off on observation of the pattern and distribution of region correlations. Consequently, for the non-corrected data, five of the defined cranial regions were combined with other regions to form multi-region modules, (see Additional file 1: Table S1), resulting in a ten-module model of modularity. The ten modules were: maxillopalatine (combining lateral, interdental plate, and palatine shelf regions of the maxillopalatine), quadrate (combining lateral and jaw joint surfaces), occipital (combining occipital region and occipital condyle), frontal-nasopremaxilla, ventral os basale-vomer, parietal, pterygoid, stapes, squamosal, and nasopremaxilla (palatal surface) (see Additional file 1: Figure S1, for region definitions).

A very similar pattern of trait correlations was obtained following jackknife resampling of our shape data down to 10% of our original landmark and semilandmark dataset, with the mean result proving near-identical to the full run using all shape data (Additional file 1: Figure S2a and Table S2). EMMLi analysis using only landmarks yielded a broadly similar pattern of modularity to the complete dataset (Additional file 1: Figure S2b and Table S3), although there were some differences worth noting. Some between-region trait correlations were higher (e.g., between the frontal and parietal), which is expected because the sampled landmarks are largely located along sutures with neighbouring bones and thus are likely to recover greater integration of those elements. In addition, within-region trait correlations were generally smaller, which is a result of the landmarks typically occupying extreme positions (e.g., sutures) within each region. These trait correlations therefore likely underestimate within-region trait correlations and exaggerate between-region trait correlations, because they may not be representative of the entire surface, or the entire shape, of a region. Consequently, the landmark-only analysis suggested the caecilian crania were more integrated than the analysis with the complete dataset.

**Fig. 1:**
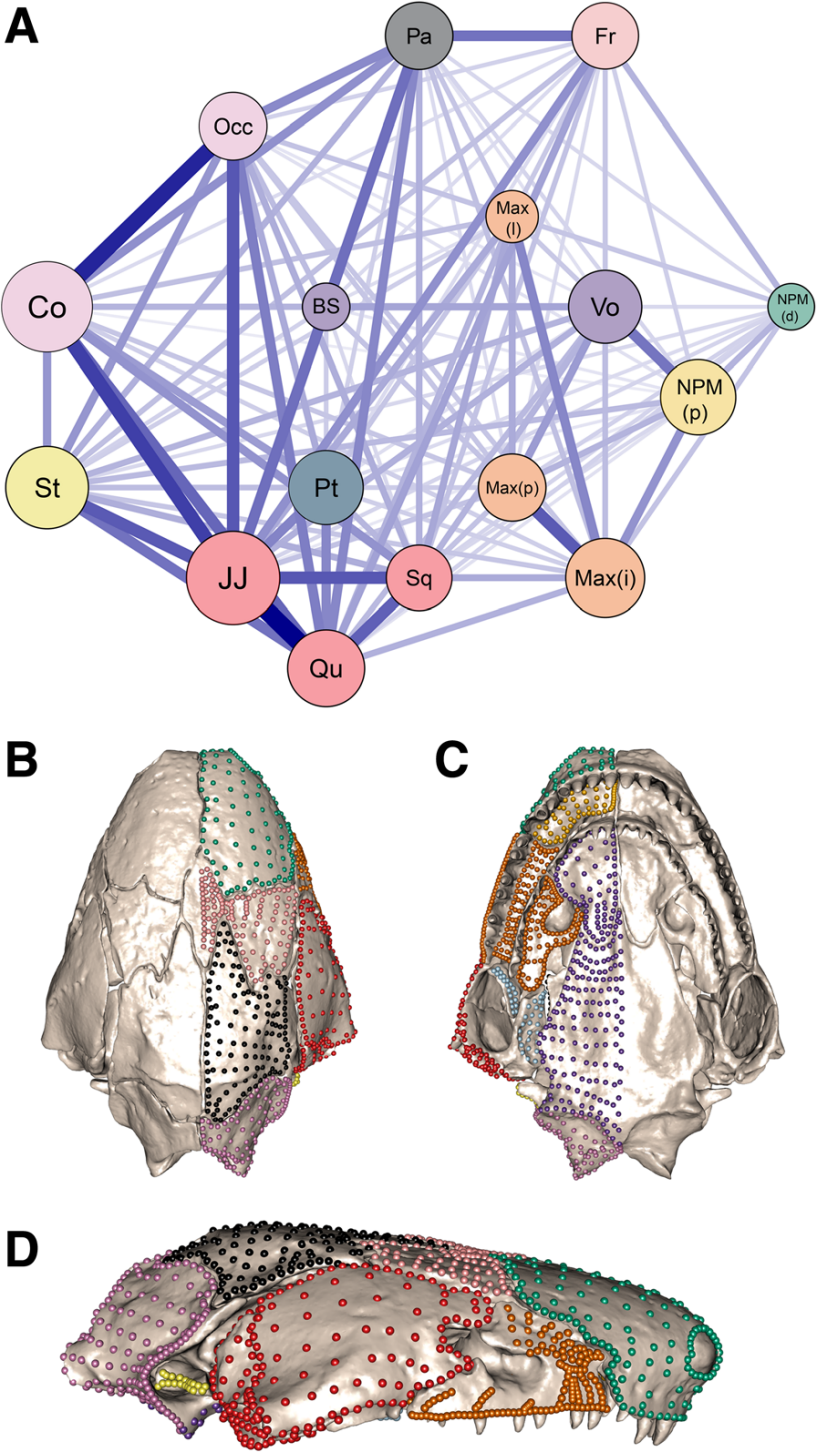
The ten-module model identified from the 16 cranial regions. **a** Network graph of the results from phylogenetically-corrected EMMLi analysis, showing the 16 cranial regions defined in this study, colour-coded by the ten identified modules. Regions were grouped into modules when the between-region trait correlation (represented by line thickness) was within 0.2 of the lowest internal trait correlation (represented by circle size). The resulting ten modules are visualised on *Siphonops annulatus* in (**b**) ventral, (**c**) dorsal and (**d**) lateral views. The ten modules comprise the following grouping of regions (see Additional file 1: Figure S1, for region definitions): **Fr (light pink)**: frontal (Fr); **Pa (black)**: parietal (Pa); **NPM(d)** (green): nasopremaxilla (dorsal) (NPM(d)); **Max (orange)**: maxillopalatine (lateral surface (Max(l)), interdental plate (Max(i)), palatine shelf (Max(p))); **Occ (light purple)**: os basale (occipital region (Occ) and occipital condyle (Co)); **Qu-Sq (red)**: quadrate (lateral surface (Qu) and jaw joint articular surface (JJ)) and squamosal (Sq); **BS-Vo (purple)**: ventral surface of os basale (BS) and vomer (Vo); **NPM(p) (gold)**: palatal surface of nasopremaxilla; **Pt (light blue)**: pterygoid (Pt); **St (yellow)**: stapes (St)

Analyses conducted after accounting for allometric and phylogenetic effects resulted in similar patterns of trait correlation among cranial regions (Additional file 1: Figure S2c-d and Table S4–S5). The same ten-module model was recovered as best fitting (following our post-hoc combining of some regions) from the allometry-corrected EMMLi analysis. However, the phylogenetically-corrected analysis recovered a slightly different ten-module model, in which the frontal and nasopremaxilla (dorsal surface) were in separate modules, but the squamosal was grouped with the lateral and jaw joint surfaces of the quadrate (Fig. 1). The ten modules for the phylogenetically-informed model were therefore the following: maxillopalatine (combining lateral, interdental plate, and palatine shelf regions of the maxillopalatine), quadrate-squamosal (combining lateral and jaw joint surfaces of the quadrate, and the squamosal), occipital (combining occipital region and occipital condyle), frontal, ventral os basale-vomer, parietal, pterygoid, stapes, nasopremaxilla (dorsal surface) and nasopremaxilla (palatal surface). We based our subsequent analyses on this ten-module model, because it takes into account shared evolutionary history.

Covariance ratio (CR) analysis yielded a largely similar pattern of cranial modularity to the best fitting model determined by EMMLi (Additional file 1: Table S6), with a significantly modular structure (*CR* = 0.59, *p *< 0.01). Concordant with EMMLi analysis, the strongest covariation among the 16 cranial regions was between the jaw joint and lateral surfaces of the quadrate (*CR =* 0.97) and between the occipital region and occipital condyle (*CR =* 0.94). The lateral surface of the quadrate and the squamosal showed relatively strong covariation (*CR* = 0.8), as did the ventral surface of the os basale and the vomer (*CR =* 0.79), and the three regions of the maxillopalatine (Max(l)-Max(i) *CR =* 0.73, Max(i)-Max(p) *CR =* 0.80, and to a lesser extent, Max(l)-Max(p) *CR =* 0.68). Compared with EMMLi analysis, the covariance ratio analysis identified the squamosal covarying more strongly with the parietal (*CR =* 0.84) than with the jaw joint articular surface of the quadrate (*CR* = 0.71).

Covariance ratio analysis of the landmark-only dataset revealed a similar pattern of trait correlation to that found in the original EMMLi analysis, but overall with a less-modular structure (*CR =* 0.88, *p* < 0.01), with stronger relationships between some regions, especially the frontal and parietal (Additional file 1: Table S7). The allometry-corrected CR analysis found a similar pattern of trait covariation (Additional file 1: Table S8), as did the phylogenetically-corrected CR analysis (Additional file 1: Table S9).

Running EMMLi (Additional file 1: Figure S3a and Table S10) and CR (Additional file 1: Figure S3b, Table S11) analyses with phylogenetically-corrected data for the ten-module model revealed largely concordant results, and both indicated that the cranium is more integrated posteriorly. The strongest integration between regions in both analyses was between the frontal and parietal and among the quadrate-squamosal, stapes, parietal and occipital modules. The most notable deviation between the two analyses was that CR analysis recovered relatively stronger covariation between the occipital and maxillopalatine modules, while EMMLi analysis recovered a relatively stronger correlation between the occipital and parietal modules.

### Cranial morphology

#### Morphological variation of the cranium

**Fig. 2:**
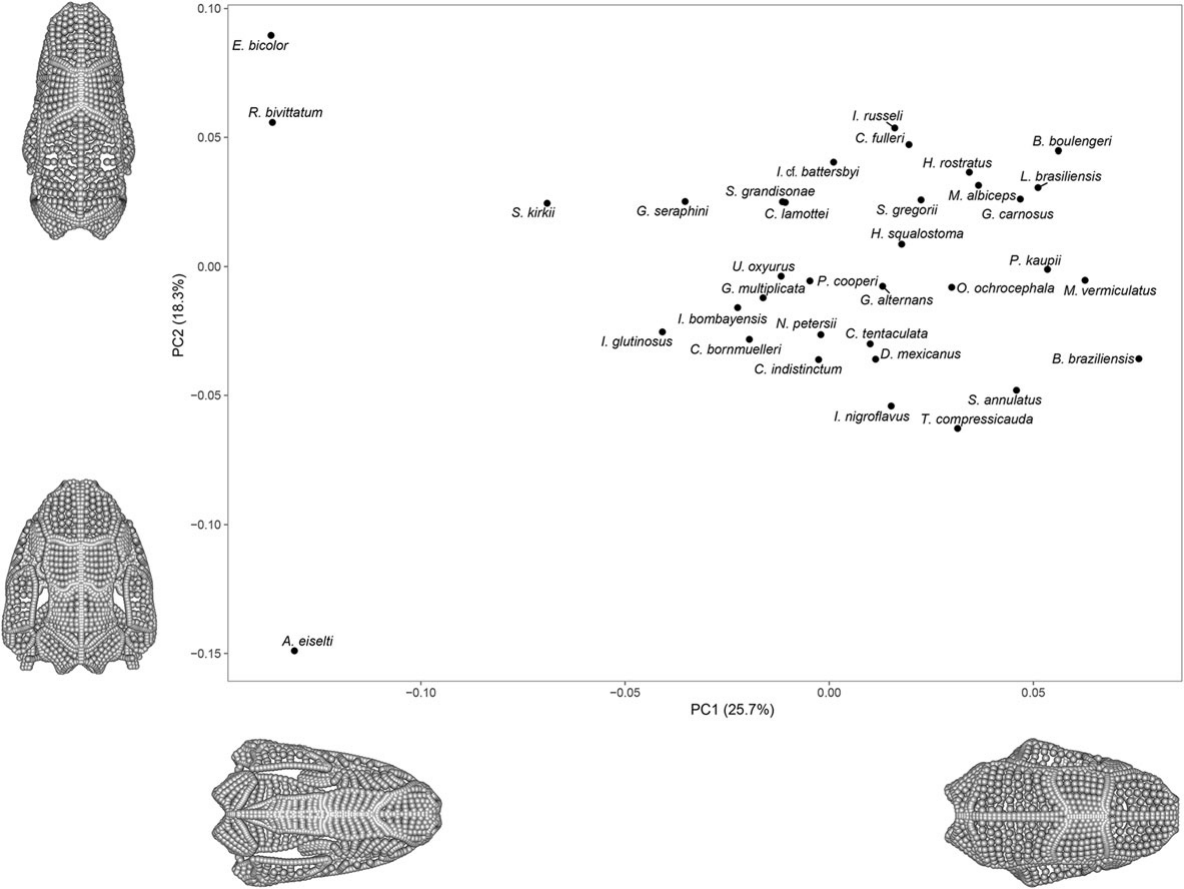
Morphospace of all 35 specimens constructed using the entire landmark and semilandmark dataset. Extreme shapes representing the positive and negative extremes along PC1 and PC2 are displayed, which are created by mirroring the landmark and semilandmark data and warping these data along PC1 and PC2. For a morphospace of PC1-PC3, see Additional file 1: Figure S7. For extreme shapes for PC1, PC2 and PC3 in dorsal, ventral, lateral, anterior and posterior aspects see Additional file 1: Figure S4–S6

Principal components analysis of the entire cranium identified 29 principal components (PCs) that explained 99% of cranial shape variation (Additional file 1: Table S12). The main variation along PC1 was related to the size of the upper temporal fenestra, the dorsoventral height of the cranium and the position of the jaw joint articulation relative to the occiput (Fig. 2, Additional file 1: Figure S4). Variation along PC2 was most obviously related to relative cranial width (Fig. 2, Additional file 1: Figure S5) and to the orientation of the maxillopalatine, squamosal, and quadrate. Along PC3, relative cranial width also varied, as well as the curvature of the ventral rim of the cranium in lateral view (Additional file 1: Figure S6).

The plot of the first two PCs (Fig. 2) most clearly separated *Epicrionops bicolor* and *Rhinatrema bivittatum* (the two sampled members of Rhinatrematidae, the sister group to all other caecilians) from the lungless (and morphologically highly disparate [65]) typhlonectid *Atretochoana eiselti.* Along PC3, *A. eiselti* was again representative of one extreme, with sampled Ichthyophiidae at the other extreme (Additional file 1: Figure S7). The distribution of taxa in a phylomorphospace (Additional file 1: Figure S8) suggested that variation in cranial morphology was somewhat phylogenetically structured, discussed further below, although close relatives were often not positioned close together.

#### Morphological variation of individual cranial modules

Distributions in morphospace varied across the individual cranial modules, and the number of PCs required to explain 99% of the variation ranged from six (stapes) to 24 (maxillopalatine). *Atretochoana eiselti* was generally the furthest from the remaining species in morphospace, most evidently for the quadrate-squamosal, stapes and occipital modules. However, *A. eiselti* occupied a similar position to other species for the nasopremaxilla (palatal surface), the pterygoid and the ventral os basale-vomer modules. Specimens of Rhinatrematidae (*E. bicolor* and *R. bivittatum*) occupied similar, extreme positions in morphospace for most cranial modules (including the parietal, quadrate-squamosal, and ventral os basale-vomer modules).

**Fig. 3:**
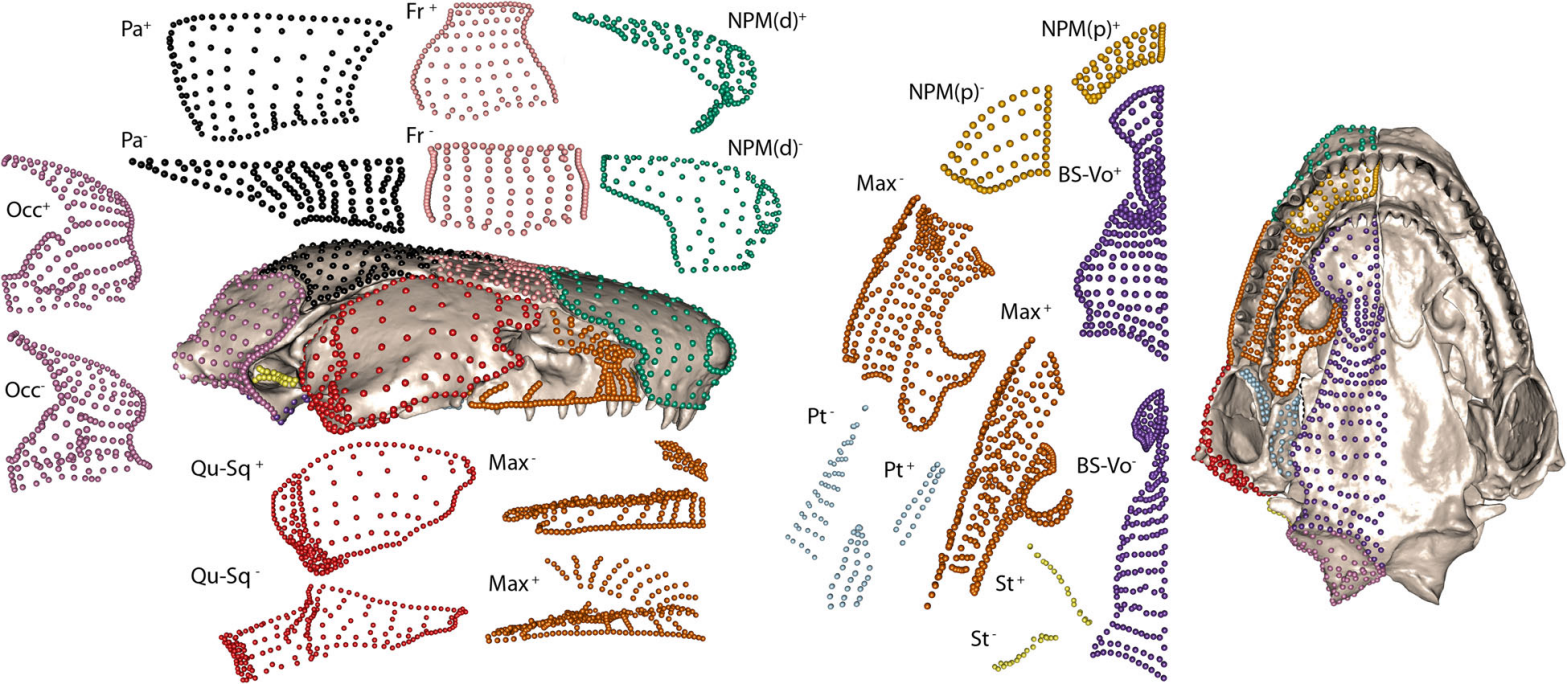
Shape variation for each cranial module. Exploded view of positive (+) and negative (−) shape extremes for each cranial module along PC1 (see Fig. 1 for module definitions). *Siphonops annulatus* is also presented in lateral (left) and ventral (right) aspect, with the landmarks and semilandmarks coloured by module. Extreme shapes were generated from individual module PCAs, so PC axes do not align and direction is arbitrary. All modules are presented in one view, except the maxillopalatine module (two views). Aspect of modules is consistent with cranial aspect, except for the occipital (posterior view) and the parietal and frontal (dorsal view). Specimens lacking a pterygoid or stapes module were removed from these PCAs, for visualisation purposes only

Shape variation for each module was assessed from the extreme shapes along PC1 (Fig. 3), and specimens closest to each extreme were identified (see Additional file 1: Figure S9–S18, for module morphospaces and extreme shapes). The main variation in the parietal module (Additional file 1: Figure S9) represented the bony enclosure of the vault, from a stegokrotaphic (closed) cranium (e.g., *Mimosiphonops vermiculatus*), to a zygokrotaphic cranium (with a large upper temporal fenestra, e.g., *E. bicolor*). The frontal module varied in shape from having approximately parallel anterior and posterior margins (e.g., *A. eiselti*) to laterally diverging anterior and posterior margins (*Ichthyophis nigroflavus*) (Additional file 1: Figure S10). The quadrate-squamosal module (Additional file 1: Figure S11) varied from an anteroposteriorly elongate squamosal and lateral surface of the quadrate, and transversely oriented jaw joint surface (e.g., *A. eiselti*), to a dorsoventrally taller squamosal, anteroposteriorly compressed lateral surface of the quadrate and more dorsoventrally oriented jaw joint (e.g., *Potomotyphlus kaupii*). The latter two species also represented the extremes for the shape of the stapes module (after removing specimens in which this module is absent, Additional file 1: Figure S12), from projecting posteriorly (*A. eiselti*) to anteriorly (*P. kaupii*). The pterygoid module (after removing specimens in which this module is absent) varied in the number of surfaces, from one (e.g., *Oscaecilia ochrocephala*) to two (e.g., *Ichthyophis bombayensis*), because this region was represented by the pterygoid and/or pterygoid process of the quadrate (Additional file 1: Figure S13). The main axis of variation for the dorsal surface of the nasopremaxilla module related to a relatively smaller naris and larger bony surface lateral to the naris at one extreme (e.g., *Brasilotyphlus braziliensis*) and a relatively larger naris and smaller bony surface lateral to the naris at the other extreme (e.g., *Crotaphatrema lamottei*) (Additional file 1: Figure S14). The depth of this module lateral to the naris revealed the variable presence of the tentacular foramen occupying this position. The maxillopalatine module (Additional file 1: Figure S15) has changed shape in response to its housing of the orbit or tentacular foramen (or both). A large maxillopalatine laterally was associated with a narrower ventral surface and larger contribution of the palatine shelf to the choana (e.g., *E. bicolor*), with *A. eiselti* at the other extreme. The palatal surface of the nasopremaxilla (Additional file 1: Figure S16) varied in anteroposterior depth, from deeper (e.g., *Crotaphatrema bornmuelleri*) to less tall (*E. bicolor*). The anteroposterior elongation of the fenestra ovalis was reflected in the lateral margin of the occipital module (Additional file 1: Figure S17), which varied from elongate anteroposteriorly (e.g., *Boulengerula boulengeri*) to approximately circular (e.g., *A. eiselti*). This module also incorporated variation in the position of the occipital condyle, likely reflecting differences in the orientation of the cranial-vertebral articulation. For the ventral os basale-vomer module (Additional file 1: Figure S18), the posterior process of the vomer extended further posteriorly and partially overlaid the ventral surface of the os basale at one extreme (e.g., *O. ochrocephala*), whereas at the other extreme the posterior process of the vomer was more lateral to the ventral surface of the os basale (e.g., *E. bicolor*).

#### Phylogeny

**Fig. 4:**
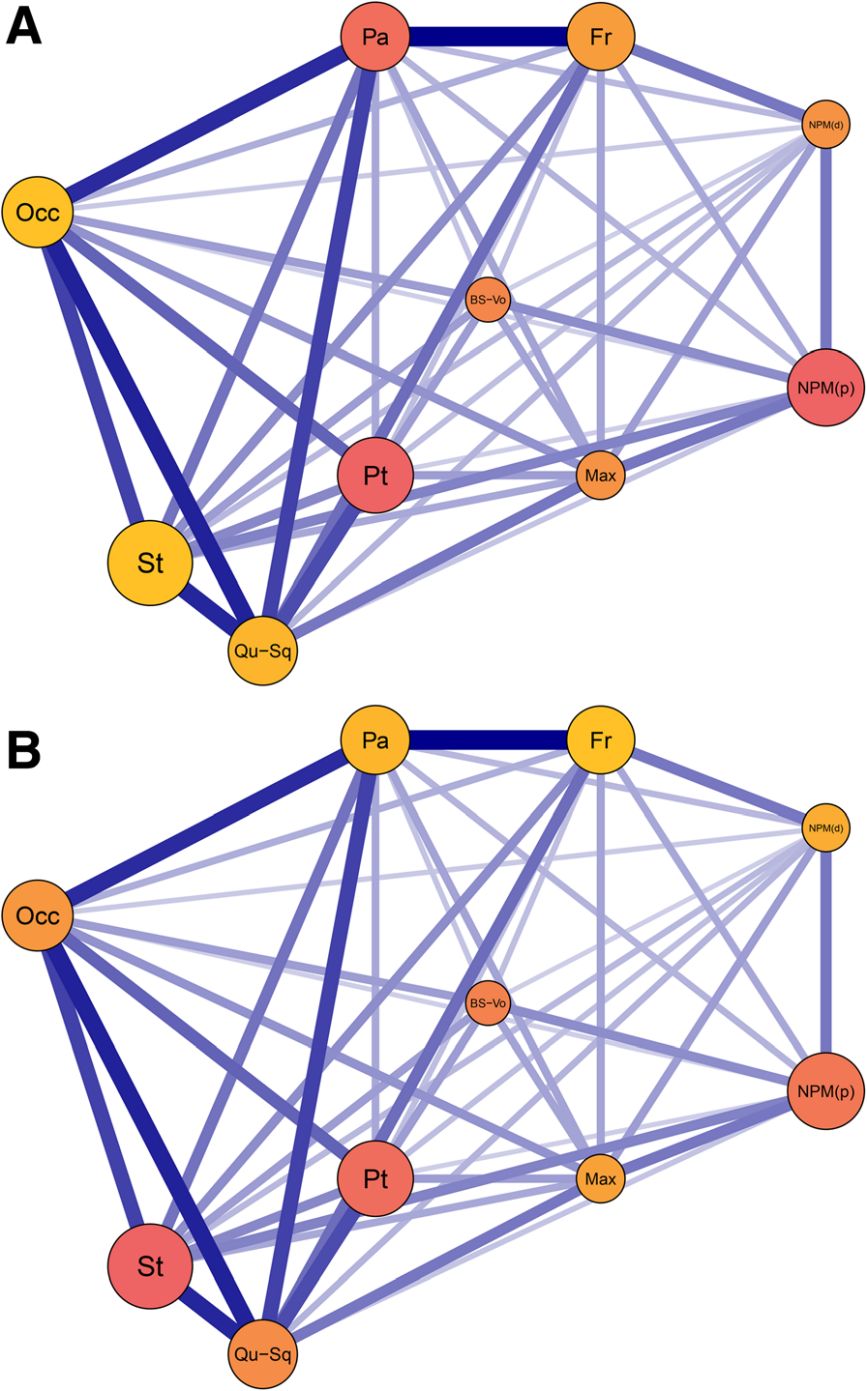
Influence of phylogeny and allometry across the ten cranial modules. Network graphs from EMMLi analysis of the ten-module model. Modules are graded low (yellow) to high (red) based on (**a**) phylogenetic signal (*K*_mult_) and (**b**) evolutionary allometry (*R*^*2*^). Circle size is proportional to internal trait correlation; line thickness is proportional to between-module trait correlation. Layout corresponds approximately to a cranium in right lateral view. See Fig. 1 for module definitions

Significant phylogenetic signal in cranial shape was found (*K*_mult_ = 0.87, *p* < 0.01). The degree of phylogenetic signal varied across cranial modules, being weaker for the posterior of the cranium (Fig. 4a and Table 1). Shape was not explained by phylogeny for one posterior module: the occipital (*K*_mult_ = 0.66, *p =* 0.06). For the stapes and quadrate-squamosal modules, phylogenetic signal was significant but relatively low (*K*_mult_ = 0.70, *p =* 0.04 and *K*_mult_ = 0.72, *p =* 0.02 respectively). Shape variation for all remaining cranial modules had stronger phylogenetic signal (*K*_mult_ = 0.87–1.16, *p* < 0.01 for all). The considerably weaker phylogenetic signal observed posteriorly in the cranium was, at least in part, explained by *A. eiselti*, because of its particularly extreme quadrate, squamosal and stapes morphologies. Results of analyses with and without this species are reported in Table 1. Variation in cranial size, as measured by centroid size (Additional file 1: Table S13), was not significantly phylogenetically structured (*K*_mult_ = 0.65, *p =* 0.18).

**Table 1:** Evolutionary rate, disparity, and integration and allometric, phylogenetic, and ecological signal in caecilian cranial modules

Module	Rate *σ*^*2*^_mult_ (×10^−8^)	Disparity (Procrustes variance) (× 10^− 6^)	Within-module correlation	Evolutionary allometry (*R*^*2*^)	Phylogenetic signal (*K*_mult_)	Phylogenetic signal (*K*_mult_), excluding *Atretochoana*	Fossoriality phylogenetic ANOVA (*R*^*2*^)
Frontal	1.33	6.32	0.70	0.05	0.87***	1.02***	0.05*/
Parietal	1.67	8.43	0.71	0.22***	1.05***	1.32***	0.10***/**
Nasopremaxilla (dorsal)	1.19	5.86	0.49	0.06*	0.93***	0.97***	0.03/
Maxillopalatine	1.64	7.84	0.50	0.19***	0.93***	0.97***	0.05**/*
Occipital	1.25	4.71	0.73	0.37***	0.66	0.75**	0.03/
Quadrate-Squamosal	2.73	10.24	0.71	0.21***	0.72*	1.12***	0.06*/*
Ventral os basale-vomer	0.91	4.97	0.46	0.12**	0.99***	1.03***	0.04/
Nasopremaxilla (palatal)	0.97	5.51	0.79	0.11*	1.16***	1.20***	0.06*/
Pterygoid	1.65	10.60	0.78	0.11*	1.13***	1.21***	0.01/
Stapes	1.28	4.76	0.87	0.19***	0.70*	1.05***	0.03/

Results for the ten identified cranial modules, where the within-module correlations are taken from EMMLi analysis using phylogenetically-corrected data. Significance of results for the last four columns is as follows: *p* values significant at the following alpha levels: * ≤ 0.05, ** ≤ 0.01, *** ≤ 0.001. Significance for differences in module shape related to fossoriality is before/after multiple-test correction. (See Fig. 1 for module definitions)

#### Allometry

Visualising morphological changes of the cranium associated with allometry found that a smaller cranial size was associated with a narrower, more elongate cranium, an absence of an upper temporal fenestra, and anterior placement of the jaw joint (Additional file 1: Figure S19, and S20, for a morphospace colour-graded by centroid size). Reconstructed morphologies were also generated for changes associated with size for each module (see Additional file 1: Figure S21). Smaller size of the occipital module was associated with relatively larger otic capsules (see Additional file 1: Figure S22).

Evolutionary allometry (accounting for phylogeny) for the entire cranium accounted for 16% of the shape variation (*R*^*2*^ = 0.16, *p* < 0.01). Allometry was not found to influence all cranial modules equally, with a stronger influence generally observed in posterior modules (Fig. 4b and Table 1). The occipital module was the most strongly influenced by allometry (*R*^*2*^ = 0.37, *p* < 0.01). Only the frontal module was nonsignificant for the allometric influence on morphology (*R*^*2*^ = 0.05, *p =* 0.14). Allometry accounted for 6–24% of the shape variation in the remaining modules.

#### Ecology

Reproductive strategy (*N* = 33) and degree of fossoriality (*N* = 35) were found to be small but significant influences on cranial shape after phylogenetic correction (*R*^*2*^ = 0.03, *p =* 0.02 and *R*^*2*^ = 0.05, *p* < 0.01 respectively), and remained significant after multiple-test correction (*p* = 0.04 and *p* = 0.01 respectively). Life history strategy (*N* = 34) was not found to be significantly associated with variation in cranial shape (*R*^*2*^ = 0.05, *p =* 0.08).

For the analyses of individual modules, phylogenetic ANOVAs found that five of the ten cranial modules exhibited a significant influence of degree of fossoriality on morphology (the parietal, quadrate-squamosal, nasopremaxilla (palatal surface), maxillopalatine and frontal modules- see Table 1). Multiple-test correction on the phylogenetic ANOVAs retained the parietal, quadrate-squamosal and maxillopalatine modules as exhibiting a significant influence of degree of fossoriality on morphology (Table 1).

#### Rate shifts associated with major ecological transitions

A significant shift in rate of morphological evolution coincided with the emergence of obligate aquatic adults (observed rate ratio of obligate to non-obligate aquatic species: 5.28, *p* < 0.01), although this result was explained largely by the morphologically highly disparate (and aquatic) *A. eiselti*: a significant rate shift here was not recovered when this species was excluded. Rate shifts in morphological evolution were also identified coincident with the evolutionary origin of direct development (observed rate ratio of direct to indirect developers: 1.42 *p* = 0.04) and of viviparity (observed rate ratio of viviparous to oviparous species: 2.20, *p =* 0.01), with faster rates of morphological evolution occurring after each innovation. After multiple-test correction, all rate shifts remained significant (*p* < 0.05).

### Evolutionary rates and disparity

#### Individual landmark and semilandmark disparity and rate

**Fig. 5:**
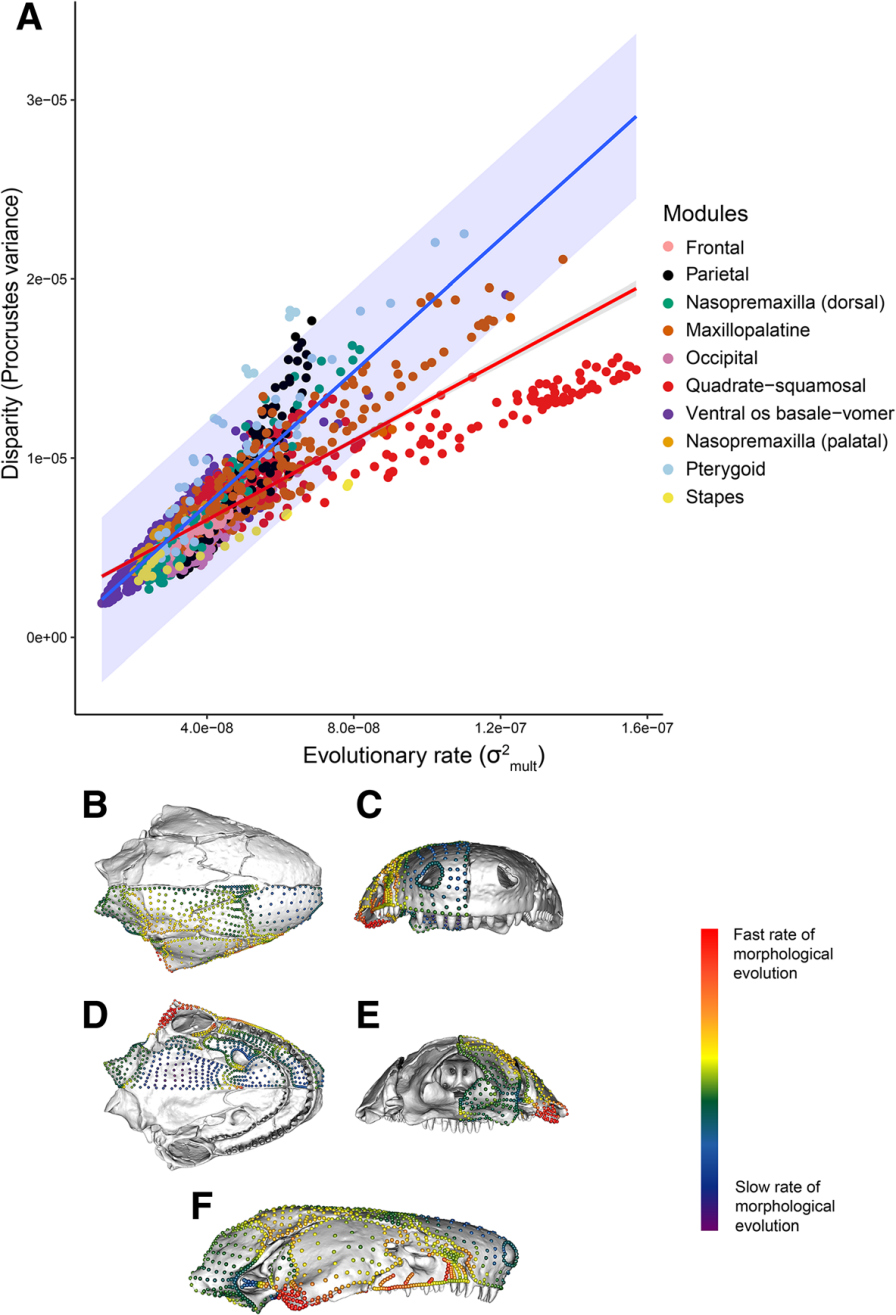
Evolutionary rate of individual landmarks and semilandmarks. **a** Regression of disparity on evolutionary rate for each landmark and semilandmark, colour-coded by module. The red line is the regression for the entire cranium. The blue line is the Brownian motion prediction, with shaded 95% interval. **b**-**f** Landmarks and semilandmarks on the sampled *Siphonops annulatus* cranium, colour-coded by evolutionary rate from low (purple) to high (red) in (**b**) dorsal, (**c**) anterior, (**d**) ventral, (**e**) posterior, and (**f**) lateral aspect. See (Additional file 1: Figure S23) for the pattern of disparity across landmarks and semilandmarks

When disparity (Additional file 1: Figure S23) and mean rate of morphological evolution (Fig. 5) of individual landmarks and semilandmarks were visualised, high rates and disparity were found consistently for landmarks and semilandmarks close to apertures. The maxillopalatine was found to have highest disparity and evolutionary rates laterally, where the orbit and tentacular foramen are variably housed. For the palatine shelf of the maxillopalatine, the highest disparity and rates were found on its post-choanal process, reflecting the variable contribution of this bone to the choanal rim. The nasopremaxilla had highest rates and disparity laterally, coinciding with the area variably involved in housing the tentacular foramen. The parietal landmarks and semilandmarks with the highest disparity and rate of morphological evolution were typically close to the variably present upper temporal fenestra. Some localised areas within modules were found to have little variation in the disparity and rate of morphological evolution of individual landmarks and semilandmarks, exhibiting consistently high (jaw joint articulation) or low (occipital condyle) values.

Analyses of landmarks and semilandmarks within cranial modules found a wide spread of rates and disparity, with overall strong but variable correlation between these. Although most landmarks and semilandmarks followed the relationship expected for evolutionary rate and disparity under a Brownian motion model (Fig. 5), some pterygoid and parietal landmarks and semilandmarks exhibited higher disparity than expected given their reconstructed evolutionary rates. Conversely, most quadrate-squamosal module landmarks and semilandmarks (and a few stapes landmarks and semilandmarks) exhibited lower disparity than expected given their rate.

#### Module integration, disparity and rate

**Fig. 6:**
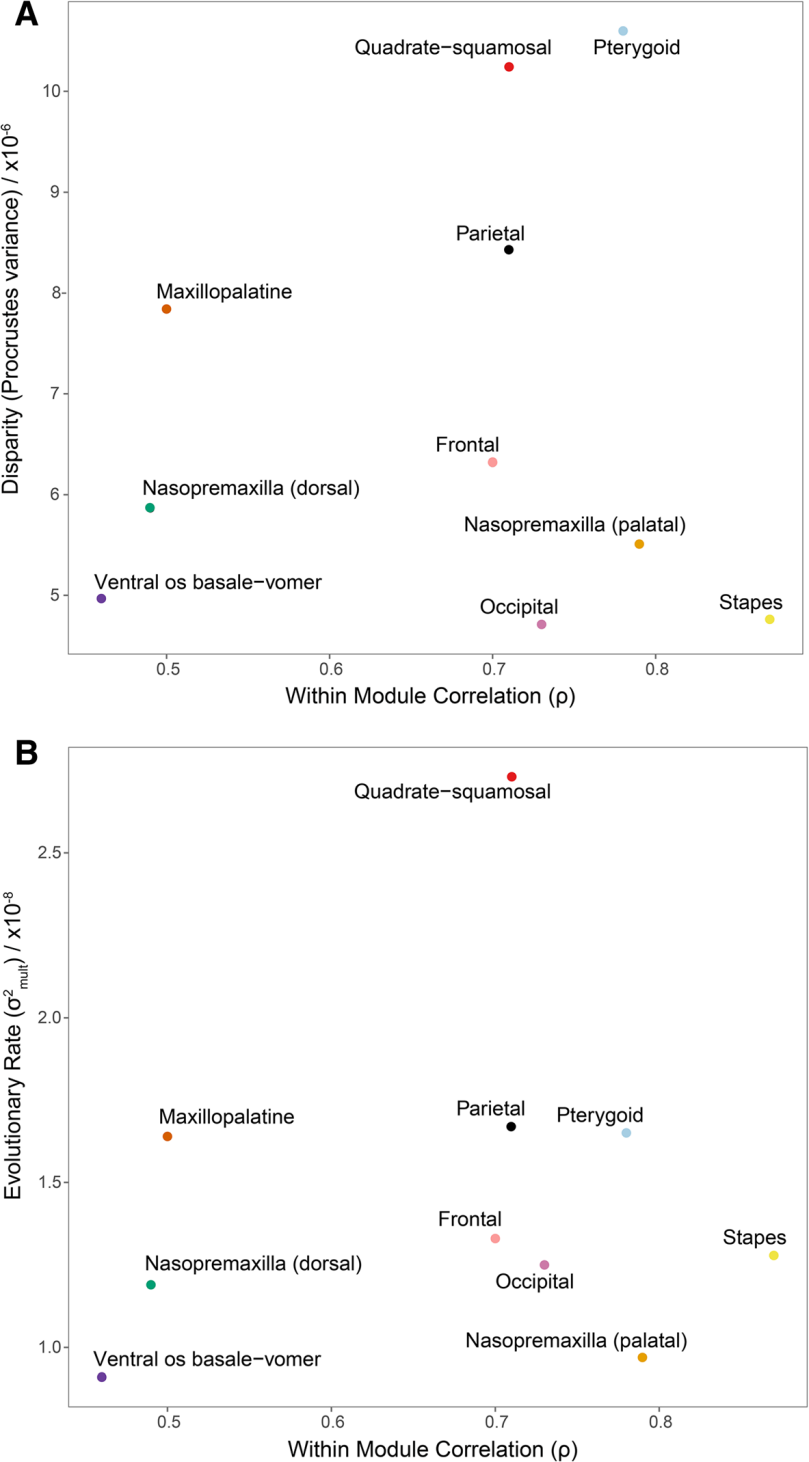
The relationship of integration with disparity and evolutionary rate. Regressions of magnitude of integration (estimated within-module correlation) on (**a**) disparity and (**b**) evolutionary rate for each cranial module (See Fig. 1 for module definitions). All relationships were non-significant

Disparity was found to be greatest in the most kinetic modules of the (generally akinetic) caecilian cranium (Table 1), with the pterygoid module exhibiting the highest disparity (1.06 **×** 10^− 5^), followed by the quadrate-squamosal module (1.02 **×** 10^− 5^). The occipital and stapes modules had the lowest disparity (4.71 **×** 10^− 6^, 4.76 **×** 10^− 6^ respectively). Most modules had significantly different disparities (Additional file 1: Table S14). Of the 45 pairwise differences between the modules, 32 were significant (*p* < 0.05, of which 24 were highly significant, *p* < 1.00 **×** 10^− 7^) and 13 were not (*p* > 0.05). The three largest differences were the pterygoid module with the occipital, stapes and ventral os basale-vomer modules, respectively. There was a nonsignificant relationship between integration and disparity (Fig. 6a) (Multiple *R*^*2*^ < 0.01, *p =* 0.90). The pterygoid and quadrate-squamosal modules had high integration and disparity. However, the stapes, nasopremaxilla (palatal surface) and occipital modules all had high integration but very low disparity. Low integration and disparity was found for both the dorsal surface of the nasopremaxilla and the ventral os basale-vomer modules. A nonsignificant relationship was also evident from the regression of rates against integration (Fig. 6b) (Multiple *R*^*2*^ < 0.01, *p =* 0.91). The quadrate-squamosal module had a significantly faster evolutionary rate than the occipital, ventral os basale-vomer and both nasopremaxilla modules, and the parietal had a significantly higher rate than the nasopremaxilla (palatal surface) module. No other rates were significantly different (see Additional file 1: Table S15).

## Discussion

### Modularity

This study provides a dense landmark and semilandmark sampling of cranial shape to quantify cranial modularity and morphological evolution across Gymnophiona. Caecilian crania are highly modular, with a ten-module model receiving most support from our high-dimensional dataset. Our model suggests that caecilian crania are more modular (in terms of number of modules) than those of mammals (e.g., [29]) and birds [19]. This may be partly because more cranial elements are present in caecilian crania, and there is greater variation in presence or absence of cranial elements compared to many other vertebrate orders. Conversely, one may have expected higher integration across caecilian crania given the relatively similar (endogeic) ecologies of most caecilians and thus more restricted functions of caecilian crania when compared with mammals and birds, though this expectation and result perhaps partly reflects a lack of knowledge about caecilian ecological diversity. Similar to birds [19], we identify an occipital module in caecilians, but we do not find distinct palatal and basisphenoid modules. Instead, one palatal surface (the vomer) forms a module with the ventral os basale (analogous to the basisphenoid region of birds), while the two maxillopalatine shelves form a module with the lateral surface of the maxillopalatine, and the palatal surface of the nasopremaxilla acts as its own module. This result suggests unusual (among the major tetrapod groups examined thus far) complexity underlying the organisation of these tooth-bearing surfaces. This is unsurprising given the specialised feeding of caecilians, including the unique dual jaw-closing mechanism, rotational feeding and typically double row of marginal teeth. Caecilian crania exhibit stronger integration posteriorly than anteriorly, with the crania more modular anteriorly. Analysis of our ten-module model indicates also that much morphological variation is localised to particular cranial modules. The subdivision of some bones into multiple modules demonstrates that the limits of osteological units do not necessarily represent the boundaries of modules, and that modules do not necessarily map directly onto traditional anatomical regions [9]. The nasopremaxilla and os basale (both formed through the fusion of multiple bones [61]) each contribute to two modules, suggesting that multiple factors influence trait integration and, ultimately, element morphology. Development of entirely exploratory methods investigating modularity using high dimensional data may help to address whether additional cranial elements may contribute to more than one module.

Our model represents the most modular pattern identified within Amphibia to date (in terms of number of modules), which may largely reflect differences in analyses. Modularity has been investigated in Gymnophiona using cranial landmarks [36], finding that (among a range of two-module models) a model separating the snout from the rest of the cranium is best-supported across three levels of variation (fluctuating asymmetry, within-, and among-species). For the anuran *Rhinella granulosa* complex, a range of best-supported patterns of modularity across species has been found, including models based on functional, hormonal-regulated and developmental modules [35]. No significant modular structure was found for the anuran family Myobatrachidae [1]. These studies did not investigate highly modular models, limiting exploration to two- [36], three- [1] and three- and five-module [35] models. Our inclusion of semilandmark data in addition to landmarks allows a denser sampling of morphology across cranial regions and reduced reliance on landmarks located predominantly at bone or region boundaries, thus facilitating a more extensive investigation of a wider range of modular structures. Previous studies of other taxa using semilandmarks have identified more modular systems (e.g., [16, 19]), suggesting, perhaps unsurprisingly, that high density morphometric data generally recover evidence for more modular structures, which may be expected in complex systems such as crania. Increasing numbers of landmarks or testing more modular patterns with landmark-only datasets may also result in more modular patterns, but this result is also likely due to the sampling of morphology that is not limited to boundaries or discrete structures. Analysis of our landmark-only dataset finds stronger correlations among adjacent cranial regions (e.g., the frontal and parietal) when compared with our full dataset, consistent with the hypothesis that integration between structures can be overestimated when using only landmarks, as these largely represent the shared boundaries of elements rather than their overall structure.

### Phylogeny and allometry

Our investigation into phylogenetic and allometric influences on caecilian cranial morphology produces broadly concurrent results to those of Sherratt et al. [60], with evolutionary allometry accounting for 14–16% of the morphological variation in both datasets. Strong phylogenetic structure is found in both studies, but our study finds greater overlap among clades in morphospace (although our morphospace distribution is not allometry-corrected). This comparison demonstrates that both approaches (landmarks and surface-based methods) recover broadly similar patterns of macroevolution but surface-based methods may provide distinct insights. Our surface-based method also reveals the interplay of phylogenetic and allometric constraints across caecilian crania. The quadrate-squamosal, occipital and stapes modules exhibit relatively weak phylogenetic signal. This low phylogenetic signal in posterior modules is partly driven by one species, *A. eiselti,* which exhibits particularly extreme quadrate, squamosal and stapes morphologies, compared with its closest relatives. *Atretochoana eiselti* is positioned especially far from *P. kaupii* in a PC1- PC2 morphospace (Fig. 2), revealing extreme morphological divergence. *Atretochoana eiselti* is also the only lungless caecilian, which has been interpreted as causally linked to its unique cranial shape [66]. Shape variation for the occipital module has low phylogenetic signal even when excluding the highly disparate *A. eiselti*, but exhibits the highest influence of allometry on shape. The size of the semi-circular canals, and their distance from the centre of the organism, is associated with improved balance [67], which is considered important for fossorial predators with reduced reliance on visual cues [68]. Maintaining a minimum size of the otic capsules may explain the strong influence of allometry observed for this module, as can be seen in the shape changes associated with allometry (Additional file 1: Figure S22). Most posterior cranial modules, although exhibiting low phylogenetic signal in shape variation, also bear the strongest indications of the influence of allometry. Conversely, modules with the strongest phylogenetic signal (nasopremaxilla (palatal surface) and pterygoid) show relatively little effect of allometry (second and third lowest across the cranium, respectively). These contrasting patterns of phylogeny and allometry suggest that cranial module size is not generally phylogenetically structured, an interpretation supported by centroid size lacking significant phylogenetic signal (*K*_mult_ = 0.65, *p =* 0.18).

### Ecology

Reproductive strategy and degree of fossoriality are found to be small but significant influences on gross cranial morphology in caecilians, even after correcting for multiple tests. Life history is not found to be a significant influence on cranial shape. Degree of fossoriality has the strongest influence on the shape of the parietal and quadrate-squamosal modules, consistent with the understanding that more dedicated fossoriality in caecilians is associated with a more solidly constructed, stegokrotaphic cranium (e.g., [52, 69]), though see [56]). A closed, stegokrotaphic cranium in caecilians is characterised by the contact of the parietal and squamosal bones and the covering of the jaw adductor muscles. In Gymnophiona, the *interhyoideus posterior* muscle has become a novel jaw-closing muscle, allowing a compensatory reduction in size of the ancestral jaw adductors and closure of the upper temporal fenestra in some species [47]. This rearrangement of jaw-closing muscles across caecilians influences the morphology of the jaw joint articular surface of the quadrate. Surprisingly, signal for the influence of degree of fossoriality on the shape of the snout (the dorsal surface of the nasopremaxilla) is relatively weak, despite the snout’s role in head-first burrowing. On a larger scale, the main axes of cranial shape variation across caecilian crania are associated with solidity (closed temporal fenestra, PC1) and elongation (bullet-shaped cranium, PC2), which are both considered to correlate with burrowing ability (e.g., [52, 69]), although may also be related to miniaturisation (because similarly-sized specimens cluster in morphospace). This investigation into the influences on cranial morphology is limited by the scarcity of ecological data (e.g., [50, 70]) and the lack of current understanding concerning the relationships among endogeicity, burrowing ability and miniaturisation for caecilians, highlighting the requirement for additional fieldwork and studies of natural history. In addition, improved knowledge of caecilian ecology would enable future studies to better characterise degree of fossoriality, without partial reliance on cranial characters.

*Atretochoana eiselti* is a clear outlier in morphospace. Our analyses concur with Wilkinson and Nussbaum’s [65] cladistic analysis, which used 141 morphological characters to resolve evolutionary relationships within Typhlonectidae, finding the terminal branch subtending *A. eiselti* to exhibit the fastest rate of morphological evolution. Together, these analyses contribute clear quantitative support for the qualitative documentation and interpretation of the extremely divergent cranial morphology of *A. eiselti* [66, 71]. As the largest lungless tetrapod and only known lungless caecilian [66, 72], the constraint of respiratory buccal pumping has been lifted, and this release (along with a reduction of constraints associated with fossoriality) is reflected in cranial morphology, with a uniquely large gape and cheek architecture [66, 73]. *Atretochoana eiselti* is a member of a clade including the three obligate aquatic typhlonectid species in our dataset, and the significant increase in the rate of cranial module shape evolution along the stem of this lineage suggests that this ecological transition promoted a faster rate of cranial evolution (although the signal for this is caused to a substantial degree by *A. eiselti*). The faster rates of cranial module shape evolution associated with the emergence of direct development and viviparity (and the significant influence of reproductive strategy on cranial morphology) indicate a role for early life-history mode substantially influencing adult cranial morphology.

### Within-module analyses

Using a surface-based approach, morphological variation can be visualised and quantified in great detail, and disparity and rates of morphological evolution can be investigated within cranial modules. Landmarks are difficult to identify consistently in the most disparate regions of caecilian crania, and thus these regions would be underrepresented using traditional landmarking approaches. Observation of the extreme morphologies along the main axes of variation for each individual cranial module allows a quantification of anatomical variation across caecilians. In addition to quantifying morphological variation within modules, disparity and rate of morphological evolution can be compared within and across modules. Disparity and rate of cranial morphological evolution are strongly correlated in Gymnophiona, and vary widely within each cranial module, with high disparity and rates particularly evident in areas associated with major cranial fenestrae and foramina. Comparing the relationship between observed landmark and semilandmark evolutionary rates and disparities with that expected under a Brownian motion model reveals that most quadrate-squamosal (and some stapes) landmarks and semilandmarks are less disparate than expected. The quadrate-squamosal module exhibits the third lowest phylogenetic signal, and the stapes is second lowest, suggesting that these modules may have undergone convergent evolution.

### How integration influences rates and disparity

Our results do not support a strong relationship between magnitude of integration and either evolutionary rate or disparity. This is similar to findings from the comparison of the crania of domestic dogs to other Carnivora, which showed that domestic dog crania are incredibly disparate (a similar magnitude to the disparity across the entire order), and yet integration and modularity of the cranium appear relatively conserved throughout domestication [31]. In our study, highly integrated modules have both more (e.g., quadrate-squamosal and pterygoid) and less (e.g., stapes and occipital) disparity than do weakly integrated modules. Among cranial modules with strong integration, the cheek region modules (quadrate-squamosal, pterygoid) display the highest disparity but the otic region modules (occipital, stapes) display the lowest. We interpret this dichotomy as indicating that integration may promote evolutionary exploration of morphospace for some cranial modules but constrain it for others, or that integration may not impact morphological disparity to any substantial degree. The caecilian cheek region is variably kinetic [48, 66], with feeding function perhaps varying among species according to diets and habitat. The otic region however may be more constrained in shape evolution, as a result of minimum requirements associated with the functionality of the sensory system housed in the otic capsules. The otic region may also be constrained by a narrower spectrum of more fundamental function in cranial-skeletal articulation. Whereas previous studies have found some support for greater integration of traits constraining morphological evolution and limiting disparity (e.g., [19]), others have found support for greater integration facilitating specialisation [9] or a mixed pattern [18]. These differences may depend on the alignment of each module’s direction of selection with the path that integration facilitates in morphospace [20, 25, 74, 75]. Our results suggest that integration may variably limit or promote morphological evolution (or have little effect), and studies of more systems are required before general patterns might be detected and exceptions explained.

We are unable to detect any significant relationship between evolutionary rate and integration, providing further evidence that integration may not necessarily influence the rate of morphological evolution [20, 25]. Not all modules have significantly different evolutionary rates, in concordance with a previous study that found the best-supported model of modularity is not necessarily fully congruent with the partitioning of shape based on variation in evolutionary rates [76]. As suggested by the “fly in the tube” model [25], integration is more likely to limit the area of morphospace in which species evolve than to limit the speed at which they move around this preferred region of space. Thus, similar rates of evolution may not be a good indicator of trait integration, and different rates of evolution do not necessarily indicate that traits are not integrated. Integrated traits may vary or evolve in a coordinated manner but at different speeds for a variety of reasons. Other factors may be a stronger influence on the pace of evolution. For example, environmental variability may be a primary driver of evolutionary rate, with low climatic variation driving both high integration and high evolutionary rate in the mandibles of canids and mustelids [77]. Furthermore, scallop shells from different ecomorphs have been found to vary in evolutionary rate but not in strength of integration, suggesting that environment may play a more important role in shaping the tempo of evolution [78]. We do however find that the fastest evolving caecilian cranial modules (pterygoid, quadrate-squamosal and parietal) have the highest disparities. Our analyses of rate of morphological evolution would be improved with the addition of fossil specimens [79] (which requires discoveries of well-preserved caecilian fossils, as the caecilian fossil record is currently very poor), and incorporating retrodeformed fossils or new undeformed fossils into future analyses will aid in refining these results. Nonetheless, the variation in rate and disparity that we observe across the caecilian cranium in this study demonstrates that the study of complex structures can benefit from identifying and analysing modules to understand localised factors shaping morphological evolution.

### The quadrate-squamosal module

The quadrate-squamosal module supported in this study corresponds to the kinetic quadrate-squamosal apparatus (QSA) previously identified [48], and stands out as the fastest evolving and second most disparate module, with the second strongest influence of fossoriality on its morphology. The QSA is believed to play an important role in the bite force of caecilians, by increasing the leverage of the jaw-closing muscles [49]. The dual jaw-closing mechanism of caecilians is unique among vertebrates, with muscles present on both sides of the jaw joint articular surface of the quadrate [47]. The rotational movements of the QSA amplify the force of the accessory jaw-closing muscle posterior to the jaw joint, the *m. interhyoideus posterior* (IHP) [48]. This streptostylic jaw joint system allows caecilians to effect a strong bite force over a range of gape angles, which may facilitate their typically generalist diet [49]. Bite force is also strong when the caecilian mouth is shut [48] which may facilitate rotational feeding, which is thought to be an important strategy for caecilians given their typically narrow gape [57]. Many caecilian species also have specialist feeding requirements early in ontogeny, with intraoviducal feeding in viviparous species and maternal dermatophagy [46] in some oviparous species. Functionally, the cheek region is therefore critical in both precocial feeding of young and generalist feeding as adults. The pivotal roles of the quadrate and squamosal in the unique dual jaw-closing mechanism may have driven the formation of the quadrate-squamosal module. This module is likely a prime target of selection, reflected in shape variation by its high disparity, rate of morphological evolution, and ecological signal, and its weak phylogenetic signal.

## Conclusions

Our high-dimensional morphological data have enabled us to quantitatively identify patterns within and across modules and across the cranium, which has provided insights into the hierarchical organisation and evolution of the caecilian cranium. Our analyses demonstrate that caecilian crania are highly modular, and that shape evolution of caecilian crania is influenced by reproductive strategy and degree of fossoriality, but the strength of this latter effect, along with the extent of phylogenetic and allometric constraints, varies across the ten identified modules. The unique dual jaw-closing mechanism and complex feeding mode of caecilians has likely driven the formation of a highly disparate and fast-evolving quadrate-squamosal ‘cheek’ module, which appears a key target of selection within caecilian crania. Overall, magnitude of module integration is not consistently associated with higher or lower shape disparity or rate of morphological evolution, suggesting that strong integration of traits variably promotes or restricts (or has little effect upon) morphological evolution of caecilian cranial modules.

## Methods

### Specimens

We generated and analysed data from the crania of 35 caecilian species, sampling all 32 extant genera and ten families (Table 2), to capture a broad range of phylogenetic and ecological diversity. All specimens were spirit-preserved and were scanned at the Natural History Museum (NHM) with a Nikon (Metris) X-Tek HMX ST 225 System and volumes were digitally dissected to create 3D isosurface models (.ply) of cranial bone using VG Studio MAX v.2.0 [80], as described by Sherratt [36]. All specimens were adults and of mixed sex, because a single-sex sample for the analysed species was not available and because interspecific variation far exceeds sex-specific variation [60]. Models were cleaned and prepared for morphometric analysis in Geomagic Wrap (3D Systems). Specifically, the external surfaces of caecilian crania are textured by neurovascular foramina and blind pits, the latter serving as attachment points for the skin [55, 81]. Small foramina hinder the application of semi-automated morphometric methods such as that used here, so these foramina were digitally filled on the cranial reconstructions using Geomagic Wrap.

**Table 2:** Specimens used in this study

Species	Family	Catalogue number	Sex	Total Length (mm)
*Atretochoana eiselti*	Typhlonectidae	NHMW 9144	F	735
*Boulengerula boulengeri*	Herpelidae	BMNH 2000.474	?F	165
*Brasilotyphlus braziliensis*	Siphonopidae	AMNH A51751	M	260
*Caecilia tentaculata*	Caeciliidae	BMNH field tag MW3945	M	533
*Chikila fulleri*	Chikilidae	DU field tag SDB1304	F	212
*Chthonerpeton indistinctum*	Typhlonectidae	MCP field tag MW16	M	325
*Crotaphatrema bornmuelleri*	Scolecomorphidae	NHMW 14859	M	275
*Crotaphatrema lamottei*	Scolecomorphidae	BMNH field tag MW5741	M	265
*Dermophis mexicanus*	Dermophiidae	MVZ 179395	M	415
*Epicrionops bicolor*	Rhinatrematidae	BMNH 78.1.25.48	F	230
*Gegeneophis carnosus*	Indotyphlidae	UK field tag MW295	M	155
*Geotrypetes seraphini*	Dermophiidae	BMNH field tag MW4543	M	195
*Grandisonia alternans*	Indotyphlidae	BMNH 1956.1.13.39	M	220
*Gymnopis multiplicata*	Dermophiidae	BM1907.10.9.10	M	460
*Herpele squalostoma*	Herpelidae	BMNH field tag MW4534	M	345
*Hypogeophis rostratus*	Indotyphlidae	UMMZ 179847	F	225
*Ichthyophis bombayensis*	Ichthyophiidae	BMNH 88.6.11.1	M	320
*Ichthyophis glutinosus*	Ichthyophiidae	NMSL field tag MW1773	F	401
*Ichthyophis nigroflavus*	Ichthyophiidae	BMNH 1967.2775	M	420
*Idiocranium russeli*	Indotyphlidae	BMNH 1946.9.5.80	F	95
*Indotyphlus* cf. *battersbyi*	Indotyphlidae	AMNH 89788	?	202
*Luetkenotyphlus brasiliensis*	Siphonopidae	BMNH 1930.4.4.1	F	?
*Microcaecilia albiceps*	Siphonopidae	MCZ A-58412	?	181
*Mimosiphonops vermiculatus*	Siphonopidae	KUH 93271	?F	185
*Nectocaecilia petersii*	Typhlonectidae	BMNH 61.9.2.6	F	590
*Oscaecilia ochrocephala*	Caeciliidae	MCZ 4268	F	330
*Potomotyphlus kaupii*	Typhlonectidae	IRNSB 12447	?	355
*Praslinia cooperi*	Indotyphlidae	BMNH 1907.10.15.154	F	165
*Rhinatrema bivittatum*	Rhinatrematidae	BMNH field tag MW2395	F	229
*Schistometopum gregorii*	Dermophiidae	MCZ 20143	F	300
*Scolecomorphus kirkii*	Scolecomorphidae	BMNH 2005.1388	F	380
*Siphonops annulatus*	Siphonopidae	BMNH 1956.1.15.88	M	340
*Sylvacaecilia grandisonae*	Indotyphlidae	BMNH 1969.1589	F	259
*Typhlonectes compressicauda*	Typhlonectidae	BMNH field tag MW5820	M	305
*Uraeotyphlus oxyurus*	Ichthyophiidae	MNHN 1994.419	M	256

Specimens are from the following institutions: American Museum of Natural History, New York, USA (AMNH), Natural History Museum, London, UK (BMNH), Delhi University, New Delhi, India (DU), Institut royal des Sciences naturelles de Belgique, Brussels, Belgium (IRSNB), University of Kansas, Museum of Natural History, Lawrence, USA (KUH), Museu de Ciências e Tecnologia da PUCRS, Porto Alegre, Brazil (MCP), Muséum national d’Histoire naturelle, Paris, France (MNHN), Museum of Vertebrate Zoology, Berkeley, USA (MVZ), Naturhistorisches Museum, Zoologische Abtheilung, Vienna, Austria (NHMW), National Museum of Sri Lanka, Colombo, Sri Lanka (NMSL), University of Kerala, Thiruvananthapuram, India (UK), University of Michigan Museum of Zoology, Ann Arbor, USA (UMMZ)

Models were decimated in Geomagic Wrap down to approximately 700,000 faces, reducing computational demands for morphometric data collection (detailed below) without compromising detail. Mirroring of models was implemented for specimens whose right-hand side was damaged (*Praslinia cooperi*, *E. bicolor*), using the ‘mirror’ function in Geomagic Wrap, and subsequent data collection was performed on only the right side of the cranium.

### Phylogeny

Phylogenetic relationships and relative divergence dates among sampled taxa are important for reconstructing evolutionary modularity and macroevolutionary patterns. A phylogeny was constructed (Fig. 8) for our taxon sampling by modifying San Mauro et al.’s [82] Bayesian relaxed-clock timetree (See Additional file 1: Figure S2 from [82]) that sampled 20 of the species used in the present study. The following modifications were made. Seven species were directly swapped with their monophyletic corresponding congener sampled in San Mauro et al.’s [82] tree: we added *Microcaecilia albiceps, Grandisonia alternans, Indotyphlus* cf. *battersbyi, Gegeneophis carnosus, Typhlonectes compressicauda, Scolecomorphus kirkii* and *C. bornmuelleri* in place of *M. sp, G. alternans, I. maharashtraensis, G. ramaswamii, T. natans, S. vittatus* and *C. lamottei,* respectively. *Ichthyophis nigroflavus* replaced *(I. bannanicus + I. asplenius)* and *E. bicolor* replaced *E.* cf. *marmoratus. Gymnopis* is paraphyletic in San Mauro et al.’s tree, so we added *G. multiplicata* as sister to *Dermophis* arbitrarily halfway along the equivalent branch to where San Mauro et al.’s two *Gymnopis* specimens join the tree. The remaining species in our dataset lack congeners in San Mauro et al.’s tree, so were placed into the phylogeny as follows: *A. eiselti* was added as sister to (*Typhlonectes + Potomotyphlus*) based on Maciel et al. 2017 [83], arbitrarily three-quarters of the way along the branch subtending (*Typhlonectes + Potomotyphlus*); *Nectocaecilia petersii* was then added as sister to (*Typhlonectes* + *Potomotyphlus* + *Atretochoana*) based on Wilkinson & Nussbaum [65], arbitrarily halfway along the internal branch subtending that clade; *B. braziliensis* was added as sister to *Microcaecilia* (see e.g., [37, 84]), placed halfway (arbitrarily) between *M. albiceps* and the split between that species and its divergence from other (non-*Brasilotyphlus*) siphonopids*; M. vermiculatus* was added (halfway, arbitrarily) between the *Siphonops*-*Luetkenotyphlus* divergence and their divergence from *Microcaecilia + Brasilotyphlus* (based on [37, 85]); *Sylvacaecilia grandisonae* was added as sister to ((*Indotyphlus* + *Gegeneophis*) + (*Praslinia* + *Hypogeophis* + *Grandisonia*)) based on Wilkinson et al. [37] halfway (arbitrarily) between the internal branch subtending that clade and its split from *Idiocranium.*

### Ecology

Due to their secretive, mostly endogeic lifestyles and mostly tropical distributions, caecilians are seldom encountered and rank among the most poorly-known orders of extant vertebrates. Many species are known from very few specimens and there is very little published ecological information and no compilations of trait data to draw upon when investigating caecilian ecology or ecological correlates (e.g., [50, 70]). Probably all species of caecilian are capable of some burrowing but in the general absence of quantitative data on caecilian burrowing abilities and on other aspects of endogeicity we divided caecilians into five mutually exclusive categories of presumed increasing degree of fossoriality (reflecting burrowing ability and other aspects of endogeic adaptation) on the basis of a combination of basic ecological and morphological data. The analytically unordered categories are: 0. aquatic species (which sometimes burrow in soft substrates); 1. tailed species (which have relatively short and stout bodies and zygokrotaphic skulls); 2. tailless species with zygokrotaphic skulls; 3. tailless species with stegokrotaphic skulls and open orbits; and 4. tailless species with closed orbits. Terrestrial species more likely to be found in leaf litter and on the surface (and not only in deeper soils) belong to categories 1–3. This simple and unambiguous categorisation corresponds with our intuitions based on the unparalleled experience of MW and DJG with diverse caecilians (including representatives of all ten families and all but seven of the 32 currently recognised genera) in the field, and is consistent with what little ecological data have been published [52], but it merits further critical testing. To the extent that the definitions of some of these categories are based on cranial morphological features, there is the potential for a degree of circularity in our analyses, but since our analyses are based on much more than these specific features we do not consider the circularity to be vicious. We also categorised species based on terrestrial versus aquatic adults, recognising only three obligate aquatic species in our sample (*A. eiselti*, *P. kaupii* and *T. compressicauda* from Typhlonectidae).

Data on reproductive strategy (oviparity versus viviparity) and life history (with or without a larval stage) were taken from San Mauro et al. (see Fig. 4 and Additional file 1: Figure S2, both from [82]) supplemented by personal observations (Additional file 1: Table S16). Species-level data were not always available, so we categorised 13 and 14 species for life history and reproductive strategy respectively based on traits known for congeners or other closest relatives (see Additional file 1: Table S16).

### Morphometric data collection

#### Regions

To compare hypotheses of modularity, regions must be defined a priori. Division of crania into many regions allows the testing of many alternative models of modularity. We defined 16 cranial regions that could be identified across all specimens (see Additional file 1: Figure S1 and Table S17). This was the highest partitioning of the skull that was reasonably achievable, because each region needs clear defining borders to ensure regions are comparable across taxa. Some elements, such as the maxillopalatine and the os basale, could be divided into three regions, while the nasopremaxilla could be divided into two regions. These divisions represent potentially divergent functional regions of these elements, and were defined by anatomical structures such as tooth rows and muscle attachment ridges. The remaining regions in most instances were individual cranial elements, but bones that are absent (or not visible on external surfaces) in some taxa must be grouped with bones present in all taxa if regions are to be comparable across all taxa while minimizing physical gaps in the representation of the cranium. Across Gymnophiona, the nasal, premaxilla, and septomaxilla (when present) may fuse to form the nasopremaxilla [61, 86]. In addition, the prefrontal fuses to the maxillopalatine during development in most (but not all) species [61, 86]. Furthermore, the stapes, mesethmoid, and pterygoid (or ‘ectopterygoid’- see [61] for discussion) are variably present. Some regions therefore represent multiple cranial elements, which were grouped on the basis of shared developmental fate (e.g., the prefrontal with the maxillopalatine) or adjacency (e.g., the mesethmoid is closest in position to, and typically sandwiched between, the frontals on the dorsal surface of the skull roof). In most specimens, some individual cranial elements are separated by unossified tissue. Where bones constituting a single region were separated by a small gap, the gap was filled in using Geomagic Wrap.

#### Landmarks and curve semilandmarks

**Fig. 7:**
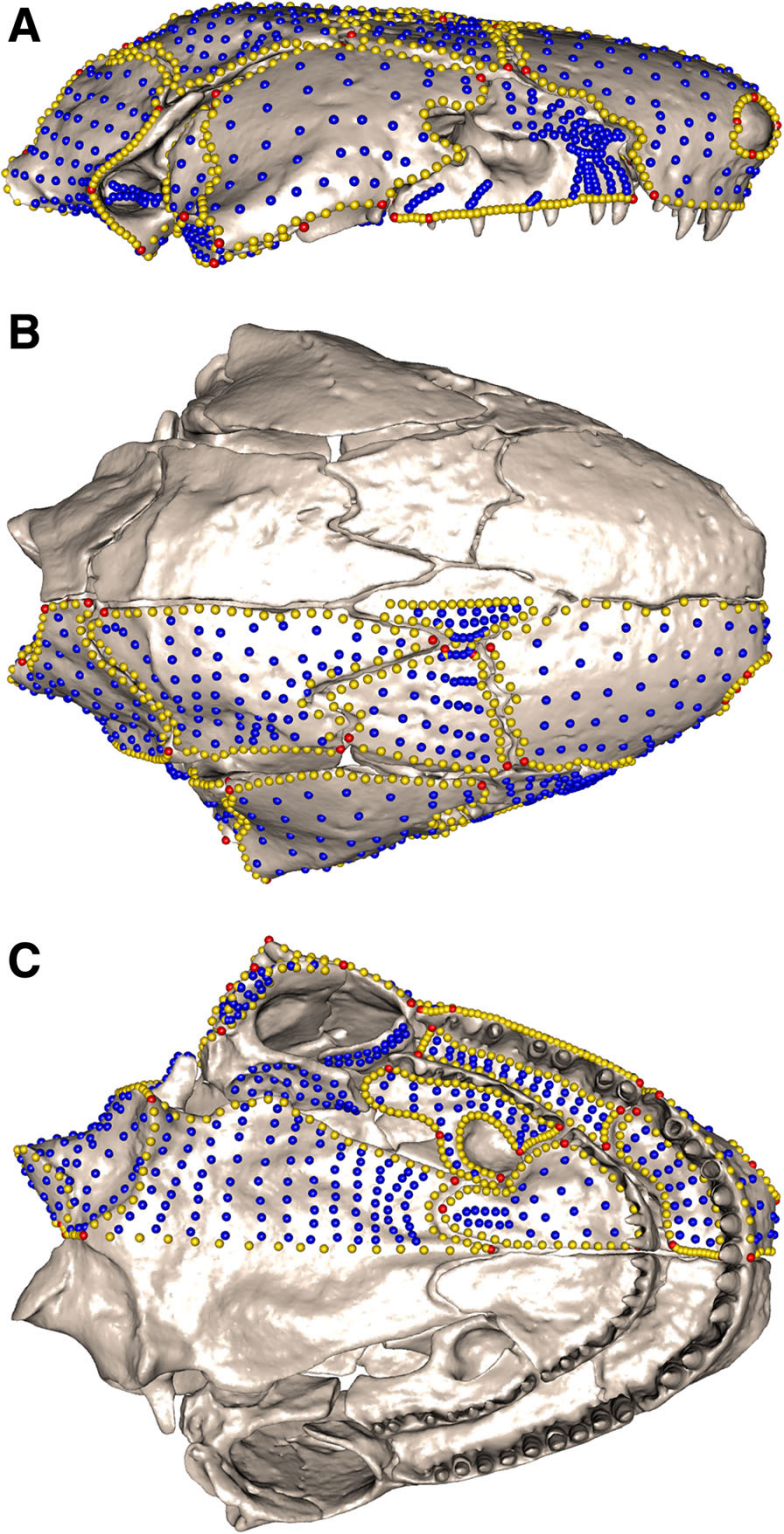
Landmark and semilandmark data. Landmarks and semilandmarks in (**a**) lateral, (**b**) dorsal and (**c**) ventral views, shown on *Siphonops annulatus*. Points are coloured as follows: landmarks (red), curve semilandmarks (yellow) and surface semilandmarks (blue)

Type I and Type II landmarks [87] and sliding semilandmarks (points regularly spaced along ‘curves’ [63]) were placed manually onto each reconstructed caecilian cranium (see Fig. 7) in IDAV Landmark Editor v.3.6 [88], to define the 16 regions (as detailed above and in Additional file 1: Figure S1 and Table S17). Fifty-three landmarks were placed on the right-hand side of the cranium, three of which were along the midline (Table 3). Curves were placed around large apertures (orbit, tentacular foramen, choana, naris) to exclude these regions during surface point digitisation (details below). Curves were also placed along tooth rows on the maxillopalatine, premaxilla, and vomer in order to exclude them from regions because of their variable presence across genera (pterygoid teeth, where present, were removed digitally in Geomagic Wrap). Fifty-seven curves were placed on the right-hand side of each cranium, anchored by landmarks, with each curve composed of between one and ten regularly-spaced semilandmarks (Additional file 1: Table S18). This resulted in 277 semilandmarks placed on each cranium. Curves were later resampled to between three and 30 semilandmarks each, with the number of semilandmarks reflecting our attempt to best capture shape (for code and a description see SI in [89]), This approach resulted in a total of 687 semilandmarks equidistantly placed along curves. During the sliding procedure, the semilandmarks were slid to minimise bending energy.

**Table 3:** Landmarks used in this study

	Landmark position
1	Nasopremaxilla, palatal surface: medial extreme of tooth row
2	Maxillopalatine, lateral surface: posterior extreme
3	Nasopremaxilla, dorsal surface: anteromedial position
4	Nasopremaxilla, dorsal surface: medial suture with frontal
5	Frontal: most lateral suture position with nasal
6	Parietal: suture with os basale along midline
7	Os basale: dorsal extreme of foramen magnum
8	Vomer: posterior extreme, medial to the choana
9	Os basale: ventral extreme of foramen magnum
10	Os basale, ventral surface: anteromedial extreme on the anteriorly projecting process
11	Quadrate: medial extreme of jaw joint articular surface
12	Quadrate: lateral extreme of jaw joint articular surface
13	Nasopremaxilla: lateral suture with frontal
14	Maxillopalatine, interdental plate: anteromedial extreme of tooth row
15	Maxillopalatine, interdental plate: posterolateral extreme
16	Maxillopalatine, interdental plate: anteromedial extreme
17	Maxillopalatine, maxillary plate: anterior extreme
18	Maxillopalatine, maxillary plate: posterior extreme of tooth row
19	Maxillopalatine, maxillary plate: inflection point where bone splits to surround the choana
20	Nasopremaxilla, palatal surface: lateral extreme of tooth row
21	Nasopremaxilla, palatal surface: posteromedial extreme
22	Vomer: medial extreme of tooth row
23	Vomer: lateral extreme of tooth row
24	Os basale, ventral surface: medial position on the muscle ridge
25	Os basale, ventral surface: lateral position on the muscle ridge
26	Os basale, ventral surface: closest position to vomer
27	Os basale: dorsal extreme of occipital condyle
28	Os basale: ventral extreme of occipital condyle
29	Os basale: posterior extreme of fenestra ovalis
30	Os basale: lateral extreme along suture with parietal
31	Squamosal: anteromedial extreme
32	Squamosal: ventral extreme along suture with maxillopalatine
33	Squamosal: posteromedial extreme
34	Squamosal: posteroventral extreme
35	Frontal: suture with nasal along midline
36	Frontal: suture with parietal along midline
37	Frontal: lateral extreme suture with parietal
38	Parietal: suture with frontal along midline
39	Parietal: anterolateral position of parietal
40	Parietal: posterolateral extreme*
41	Maxillopalatine, lateral surface: anterior extreme of tooth row (dorsal)
42	Maxillopalatine, lateral surface: posterior extreme of tooth row, behind last tooth
43	Nasopremaxilla, dorsal surface: posterolateral extreme above tooth row
44	Nasopremaxilla, dorsal surface: anterior extreme of nares opening
45	Nasopremaxilla, dorsal surface: dorsal extreme of nares opening
46	Nasopremaxilla, dorsal surface: lateral extreme of nares opening
47	Nasopremaxilla, dorsal surface: ventral extreme of nares opening
48	Quadrate, lateral surface: anterolateral extreme
49	Maxillopalatine, interdental plate: anterolateral to the most posterior tooth
50	Os basale: anteromedial suture with parietal
51	Maxillopalatine, maxillary plate: posterior extreme of choanal rim
52	Maxillopalatine, maxillary plate: anterior extreme of choanal rim
53	Quadrate, lateral surface: anteromedial extreme
54	Quadrate, lateral surface: maximum curvature of jaw joint articular surface
55	Stapes, lateral aspect: anterior extreme of the rod, positioned midway dorsoventrally*
56	Stapes: position adjacent to posterior extreme of fenestra ovalis*
57	Stapes: position adjacent to anterior extreme of fenestra ovalis*
58	Stapes: position adjacent to dorsal extreme of fenestra ovalis*
59	Stapes: position adjacent to ventral extreme of fenestra ovalis*
60	Pterygoid process of quadrate (or if absent, pterygoid): posteromedial extreme of ventral surface*
61	Pterygoid process of quadrate (or if absent, pterygoid): posterolateral extreme of ventral surface*
62	Pterygoid process of quadrate (or if absent, pterygoid): anteromedial extreme of ventral surface*
63	Maxillopalatine, lateral surface: posterodorsal suture with squamosal*
64	Maxillopalatine, lateral surface: anterodorsal extreme*
65	Maxillopalatine: if tentacular groove present, suture with nasal and frontal*
66	Maxillopalatine: tentacular groove (if present), anterior extreme*
67	Maxillopalatine: tentacular groove (if present), posterior extreme*
68	Maxillopalatine: tentacular groove (if present), ventral extreme*
69	Mesethmoid (if absent, frontal): anteromedial extreme*
70	Mesethmoid (if absent, frontal): posteromedial extreme*
71	Postfrontal (if absent, squamosal): antero-dorsal extreme*
72	Pterygoid (if present): posteromedial extreme of ventral surface*
73	Pterygoid (if present): posterolateral extreme of ventral surface*
74	Pterygoid (if present): anteromedial extreme of ventral surface*

74 landmarks were placed onto the right-hand side of the cranium of each specimen. 21 landmarks (*) were removed prior to analyses as they were not homologous across all specimens. These 21 landmarks were used to fix curves around structures such as foramina

#### Surface semilandmarks

While all landmarks and curves were placed manually onto each specimen, a template was used to fit the surface semilandmarks (‘surface points’). The template used was a generic hemispherical mesh onto which all specimen landmarks and curves were placed manually. Surface points were then placed systematically across each region, in evenly spaced rows parallel to the region margins. A semi-automated procedure in the *Morpho* package v.2.5.1 [90] in R v.3.4.3 [91] was used to apply surface points onto each specimen (Fig. 7). During this patching procedure, the template is warped to the shape of each specimen based on the shared landmarks and curves, so must be of sufficient resolution (18,000 triangles in this instance). The template’s surface points are then projected onto each specimen, and these are translated along their normals until they contact the surface of the specimen. Following this process, the curve and surface points are slid to minimise bending energy. Each region was patched globally when possible, allowing bending energy to be minimised across the whole dataset. A total of 729 surface points were placed onto each specimen (Additional file 1: Table S19).

Three regions required patching across subsets of specimens. The lateral surface of the maxillopalatine was variably subdivided into two in cases where the tentacular canal is open laterally. The pterygoid region consists of either one or two surfaces (pterygoid and/or pterygoid process of the quadrate) or is absent completely (*S. kirkii* and *C. bornmuelleri*), and the stapes is absent in three of the sampled taxa (*S. kirkii, C. bornmuelleri* and *C. lamottei*). An additional 21 landmarks (Table 3, *landmarks) and 18 curves (Additional file 1: Table S18) were placed on each specimen, to aid the patching of surface points for these highly variable regions. These were not globally homologous across specimens so were removed following patching of surface points, prior to analyses, leaving only surface points for these regions in the final dataset. Absent regions were represented by one cranial landmark (whose position best represented the location of the missing region), replicated to achieve an array of the same dimension as the surface point dataset from other specimens. A missing region is therefore captured as an infinitesimal surface, with the same dimensions as present regions. A similar approach has previously been suggested to allow for incorporation of novel structures in geometric morphometric studies [92] and here this allowed us to retain all specimens and regions for analyses. After global Procrustes alignment, missing regions had non-zero (but negligible) size. The multiple templates for each of the maxillopalatine, pterygoid, and stapes had an identical number of surface points and an analogous surface point distribution. Unless otherwise specified, the term ‘semilandmarks’ refers to all sliding curve and surface semilandmarks.

### Data analyses

#### Generalised Procrustes analysis

Generalised Procrustes analysis [93] removes non-shape aspects from landmark coordinate data [94]. Curves and surface points were mirrored prior to Procrustes analysis using the mirrorfill function in the R package *paleomorph* v.0.1.4 [95]*,* because a bilaterally symmetrical structure results in a more successful alignment than using only one side [96]. Procrustes analysis was then performed using the g*eomorph* R package v.3.0.5 [97]. Mirrored data were then manually removed from the dataset following the Procrustes alignment, such that all analyses were conducted on data representing only the midline and right-side.

#### Modularity

To assess patterns of modularity we used two methods, both implemented in R: a maximum likelihood approach (EMMLi) and the covariance ratio (CR). First, we used EMMLi with the trait correlation matrix (congruence coefficients) [28]. This approach allows alternative hypotheses of modularity to be tested, with the advantage that models with different numbers of modules can be directly compared. We used the ‘EMMLi’ function in *EMMLi* to test 15 different model structures (Additional file 1: Table S20), ranging from one module (entirely integrated cranium) to a 16-module model (each of the 16 regions is a separate module). The models we assessed include multiple two-module models (e.g., division of the cranium into anterior and posterior modules, dorsal and ventral modules, and medial and lateral modules), a six-module model analogous to the therian mammal six-module model [29] and extensions of this model by further partitioning some of the original six modules into small subunits. We also investigated models from Sherratt et al.’s [36] study of caecilian crania, testing the cheek region combined with either the snout, the braincase, or as its own module.

Because of the low specimen-to-landmark (including semilandmarks) ratio in our dataset, we assessed the robustness of our results in multiple ways. First, we analysed patterns of modularity using only landmarks (excluding the regions detailed above as being represented only by surface points). Second, we applied a random jackknife resampling of our morphometric data down to 10% (*N*_lmks_ = 147) of the original dataset (*N* = 1469), using the ‘subSampleEMMLi’ function in *EMMLiv2* (https://github.com/hferg/EMMLiv2/) with results averaged over 100 iterative runs. We further analysed patterns of modularity with EMMLi after correcting our data for phylogenetic and allometric effects. To correct for allometry we performed a Procrustes ANOVA using the ‘procD.lm’ function in *geomorph* with log centroid size as a factor, and extracted the residuals from this analysis. Centroid size is the square root of the sum of squared distances of landmarks from the structure’s centroid (centre). To account for the evolutionary relationships among species, we used phylogenetic independent contrasts of shape in these analyses [98].

We further applied a second method to assess modularity: we calculated the covariance ratio [99] for the complete dataset, landmark-only, allometry-corrected and phylogenetically-corrected data using the ‘modularity.test’ function in *geomorph*.

#### Phylogenetic, allometric and other factors influencing shape

We used principal components analysis (PCA) to identify the major axes of shape variation across caecilians for the whole cranium and for individual cranial modules. Representations of morphologies defining the extremes of the significant PC axes were then used to visualise the main components of morphological variation across the cranium and cranial modules.

We quantified phylogenetic signal (the degree of similarity explained by shared ancestry) in our shape data using the *K*_mult_ statistic [100], a multivariate generalisation of the K statistic, which calculates phylogenetic signal under the assumption of Brownian motion [101]. *K*_mult_ was calculated using the ‘physignal’ function in g*eomorph* for all cranial modules separately and for the entire cranium, using our modified phylogeny. We also estimated phylogenetic signal in our centroid size data.

The amount of shape variation explained by allometry (size-related shape change) was visualised for the cranium using a multivariate regression implemented in the ‘procD.allometry’ function in *geomorph* [102]. Individual module morphologies at maximum and minimum sizes were also visualised, by partitioning the globally aligned shape data into each module’s landmarks and semilandmarks, and investigating allometry with cranial centroid size as the factor. Global Procrustes alignment retains relative positional and scaling information, whilst local Procrustes loses this information (See [103] for a discussion).

Evolutionary allometry was quantified using a phylogenetic generalised least squares analysis for high-dimensional data [104] (‘procD.pgls’ function in *geomorph*), for the cranium and for each individual module. Global Procrustes alignment was performed, because we wanted to retain information about relative positional and scaling information of modules. Statistical significance of the factors (here, centroid size) in the model is assessed by permutation of the phenotypic data across the tips of the phylogeny, for 1000 iterations.

We performed phylogenetic ANOVAs to assess the influences of fossoriality, life history and reproductive strategies on skull shape evolution, and of the influence of degree of fossoriality for each individual module. Specimens with data lacking were removed from the relevant analyses (see Additional file 1: Table S16). We applied Benjamini-Hochberg corrections [105] for the phylogenetic ANOVAs and rate shifts, to account for elevated false positive rates. The influences of life history and reproductive strategies were not explored for each module because increased numbers of statistical tests decreases statistical power.

#### Evolutionary rates and disparity

Disparity was quantified by Procrustes variance and calculated using the ‘morphol.disparity’ function in *geomorph*, for the entire cranium and for each individual cranial module. Differences in disparity between cranial modules were evaluated using the ‘TukeyHSD’ function in R, which calculates the differences in observed means, with *p*-values adjusted for multiple tests. We divided each module’s disparity by the number of landmarks and semilandmarks included in analyses for that module to correct for landmark/semilandmark number, which affects variance estimates, and to render our results more comparable across modules. Using the dated phylogenetic tree described above, we then calculated net rates of morphological evolution for each module under a Brownian motion model using the ‘compare.multi.evol.rates’ function [76] in *geomorph*, which is an extension of the ‘compare.evol.rates’ function [106] to allow comparison across multiple phenotypic traits*.* This approach allows the direct comparison of rates across a high-dimensional modular structure, using the ratio of the maximum to minimum rate as a test statistic. Significance is evaluated through phylogenetic simulation, by obtaining tips data using a global evolutionary rate and comparing simulation rate ratios to the observed ratio (see [76]). We investigated the relationship between magnitude of integration, disparity, and rate of morphological evolution for each cranial module by plotting regressions of disparity and rates on magnitude of integration (estimated within-module trait correlation, *ρ*).

We further determined the disparity (as measured by variance) and rate of morphological evolution of each individual landmark and semilandmark (through modification of the ‘compare.evol.rates’ function in *geomorph*) and colour-graded a representative caecilian cranium’s landmarks and semilandmarks according to these metrics. Although the sliding of semilandmarks and Procrustes analysis imposes some covariance on individual data points, this approach allows for clear visualization of concentrations of unusual rate or disparity in cranial regions. We also plotted the regression of disparity on evolutionary rate for each individual landmark and semilandmark, in order to conduct a more detailed assessment of these attributes across the caecilian cranium. We compared the observed relationship between variance and evolutionary rate to a simulated expectation of variance for each given evolutionary rate. Specifically, we calculated the evolutionary rate for each landmark and semilandmark and simulated trait evolution under a model of Brownian motion, assuming no trait covariation. We ran 100 simulations, using the ‘sim.char’ function in the R package *geiger* v.2.0.6 [107]. We determined the mean variance of each landmark and semilandmark across these 100 simulations and fitted a linear regression of calculated evolutionary rate to simulated variance. We also generated a 95% prediction interval using the ‘predict’ function in R and noted which of our landmarks and semilandmarks fell outside the expected variance range for each given evolutionary rate.

**Fig. 8:**
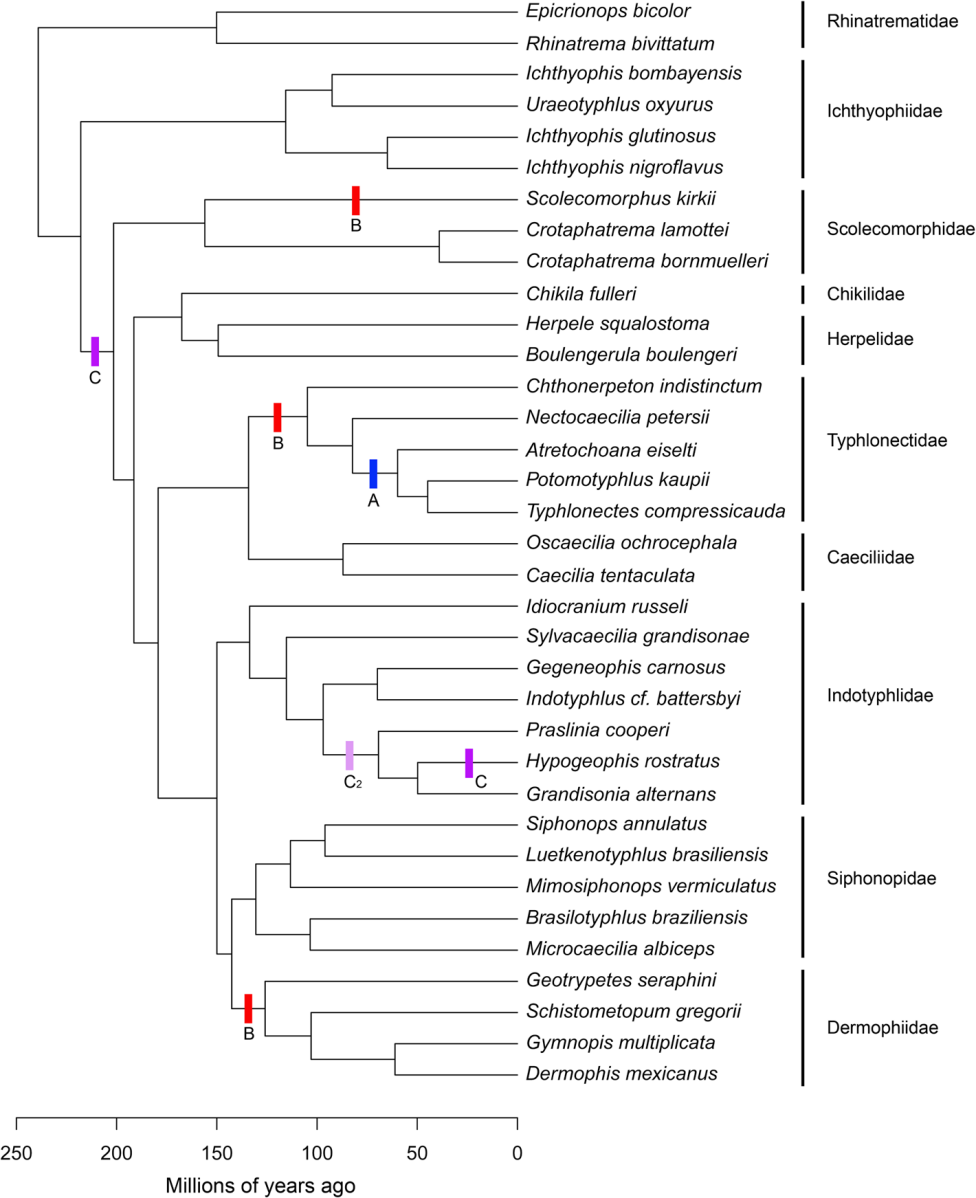
Time-calibrated phylogeny of caecilians used in this study. Modified from a Bayesian relaxed-clock timetree based on a mitogenomic dataset (See Additional file 1: Figure S2 from [82]). Scale is in million years. Node ages are based on point divergences (means) rather than confidence ranges. Vertical lines refer to rate shifts tested in our study, corresponding to (A) obligate aquatic niche, (B) viviparity, and (C) direct development, the data for which can be found in Additional file 1: Table S16. Note the re-emergence of biphasic development (C_2_)

We also tested whether shifts in rates of morphological evolution are correlated with major transitions in ecology and life history across the tree. We compared net rates of morphological evolution among obligate aquatic species and non-obligate species, among direct and indirect developers and finally among viviparous and oviparous species (Fig. 8). This was implemented in the ‘compare.evol.rates’ function in *geomorph* [106], which calculates rates of evolution under a Brownian motion model of evolution for each group, and obtains a rate ratio. Significance was assessed in the same way as with ‘compare.multi.evol.rates’ (see [76]). Within caecilians, obligate aquatic adults probably evolved only once (within Typhlonectidae), while among our sampled taxa direct development has possibly arisen at least twice, and viviparity three times (Fig. 8). We applied Benjamini-Hochberg corrections for multiple comparisons [105] for rate shifts.

## Additional files

Additional file 1: Consists of all supplementary figures and tables cited in the manuscript. This file includes 23 figures and 20 tables. (PDF 5425 kb)

Additional file 2: Consists of landmark data for the 35 specimens. (CSV 73 kb)
