# Supplementary material for: Morphological evolution and modularity of the caecilian skull
Source: BMC Evol Biol. 2019 Jan 22;19:30. doi: 10.1186/s12862-018-1342-7 (PMC6343317; doi:10.1186/s12862-018-1342-7)
Supplement: Supplementary file 1 — Consists of all supplementary figures and tables cited in the manuscript. This file includes 23 figures and 20 tables. (PDF 5425 kb) [file 12862_2018_1342_MOESM1_ESM.pdf]

Additional File 1

Morphological Evolution and Modularity of the Caecilian Skull

Carla Bardua, Mark Wilkinson, David J. Gower, Emma Sherratt, Anjali Goswami

This document includes the following supplementary contents:

**Figure S1.** Landmarks and semilandmarks colour coded by the 16 cranial regions	2
**Figure S2.** Network graphs displaying results from EMMLi analyses	3
**Figure S3.** Network graphs from EMMLi and CR analyses of the ten-module model	4
**Figure S4.** Reconstructed morphologies represented by positive and negative ends of PC1	5
**Figure S5.** Reconstructed morphologies represented by positive and negative ends of PC2	6
**Figure S6.** Reconstructed morphologies represented by positive and negative ends of PC3	7
**Figure S7.** Morphospace of all 35 specimens	8
**Figure S8.** Phylomorphospace of all specimens	9
**Figure S9.** Morphospace of the parietal module	10
**Figure S10.** Morphospace of the frontal module	11
**Figure S11.** Morphospace of the quadrate-squamosal module	12
**Figure S12.** Morphospace of the stapes module	13
**Figure S13.** Morphospace of the pterygoid module	14
**Figure S14.** Morphospace of the nasopremaxilla (dorsal) module	15
**Figure S15.** Morphospace of the maxillopalatine module	16
**Figure S16.** Morphospace of the nasopremaxilla (palatal) module	17
**Figure S17.** Morphospace of the occipital module	18
**Figure S18.** Morphospace of the ventral os basale-vomer module	19
**Figure S19.** Whole cranial morphology at (A) minimum and (B) maximum size	20
**Figure S20.** Distribution of specimens in morphospace, colour-coded by centroid size	20
**Figure S21.** Module morphologies at minimum and maximum size	21
**Figure S22.** The occipital module morphology at minimum and maximum size	22
**Figure S23.** Landmarks and semilandmarks colour coded by magnitude of disparity	23
**Table S1.** Results of EMMLi analysis for the 16 cranial regions	24
**Table S2**. Results of EMMLi analysis using subsampled data	26
**Table S3**. Results of EMMLi analysis using landmark only dataset	28
**Table S4**. Results of EMMLi analysis using allometry-corrected data	29
**Table S5**. Results of EMMLi analysis using phylogenetically-corrected data	31
**Table S6.** Covariance Ratio results for the 16 cranial regions	33
**Table S7**. Covariance Ratio results, using only anatomical landmarks	34
**Table S8**. Covariance Ratio results, corrected for allometry	35
**Table S9**. Covariance Ratio results, corrected for phylogeny	36
**Table S10.** Results of EMMLi analysis for the ten identified cranial modules	37
**Table S11**. Covariance Ratio results for the ten identified cranial modules	38
**Table S12.** Summary of the PC axes	39
**Table S13**. Centroid size of all specimens	40
**Table S14.** Differences in the observed means of disparity between the ten modules	42
**Table S15.** Significance values for differences in rates of morphological evolution	43
**Table S16**. Ecological data for all specimens in this study.	44
**Table S17.** Definitions for the 16 cranial regions analysed in this study	46
**Table S18.** Description of curves	49
**Table S19.** Number of surface points projected onto each of the cranial regions	58
**Table S20.** Alternative models of modular organisation tested in EMMLi analysis	59
References for Additional File 1	60

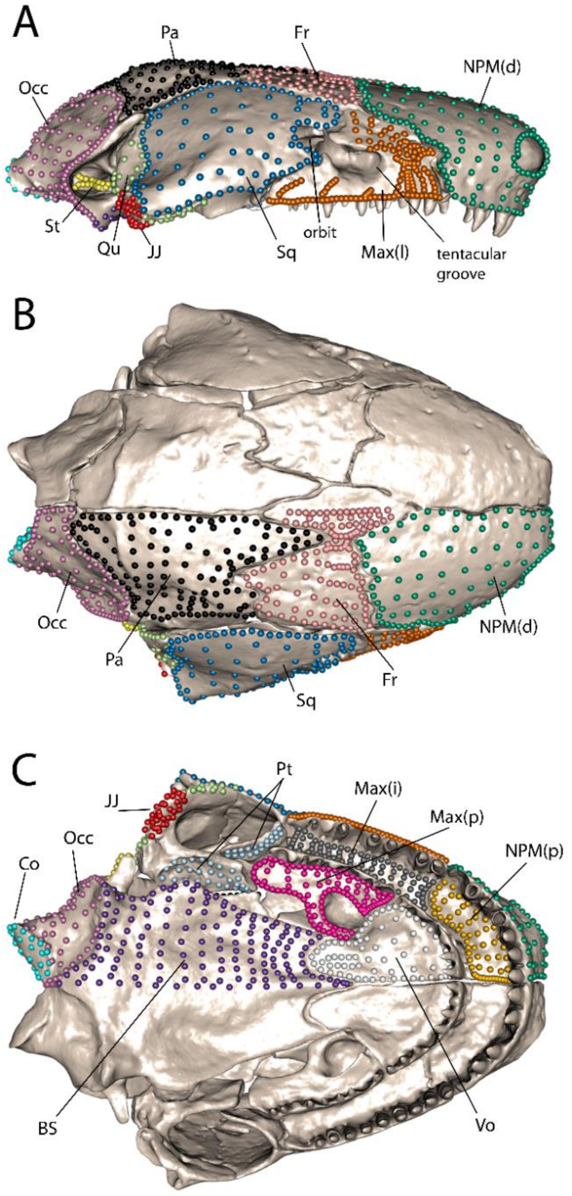

Figure S1. Landmarks and semilandmarks in (A) lateral, (B) dorsal and (C) ventral views, colour coded by the 16 cranial regions defined in our study, shown on *Siphonops annulatus*. Regions are as follows: **NPM(d) (green)**: nasal, premaxilla (or nasopremaxilla) and septomaxilla when present, dorsal surface; **Fr (light pink)**: frontal, and mesethmoid when present; **Pa (black)**: parietal; **Sq (dark blue)**: squamosal, and postfrontal when present; **Max(l) (orange)**: maxillopalatine (lateral surface), and prefrontal when present; **Qu (light green)**: quadrate (lateral surface); **JJ (red)**: quadrate (jaw joint articulation); **Occ (light purple)**: occipital (otic) region of os basale (excluding occipital condyle); **Co (aqua)**: occipital condyle; **BS (purple)**: Ventral surface of os basale; **NPM(p) (gold)**: palatal surface of nasopremaxilla, or the anterior projection of the vomer; **Vo (white)**: vomer; **Max(i) (grey)**: interdental plate of maxillopalatine; **Max(p) (hot pink)**: palatine shelf (maxillary plate) of maxillopalatine; **Pt (light blue)**: pterygoid, and/or pterygoid process of quadrate; **St (yellow)**: stapes.

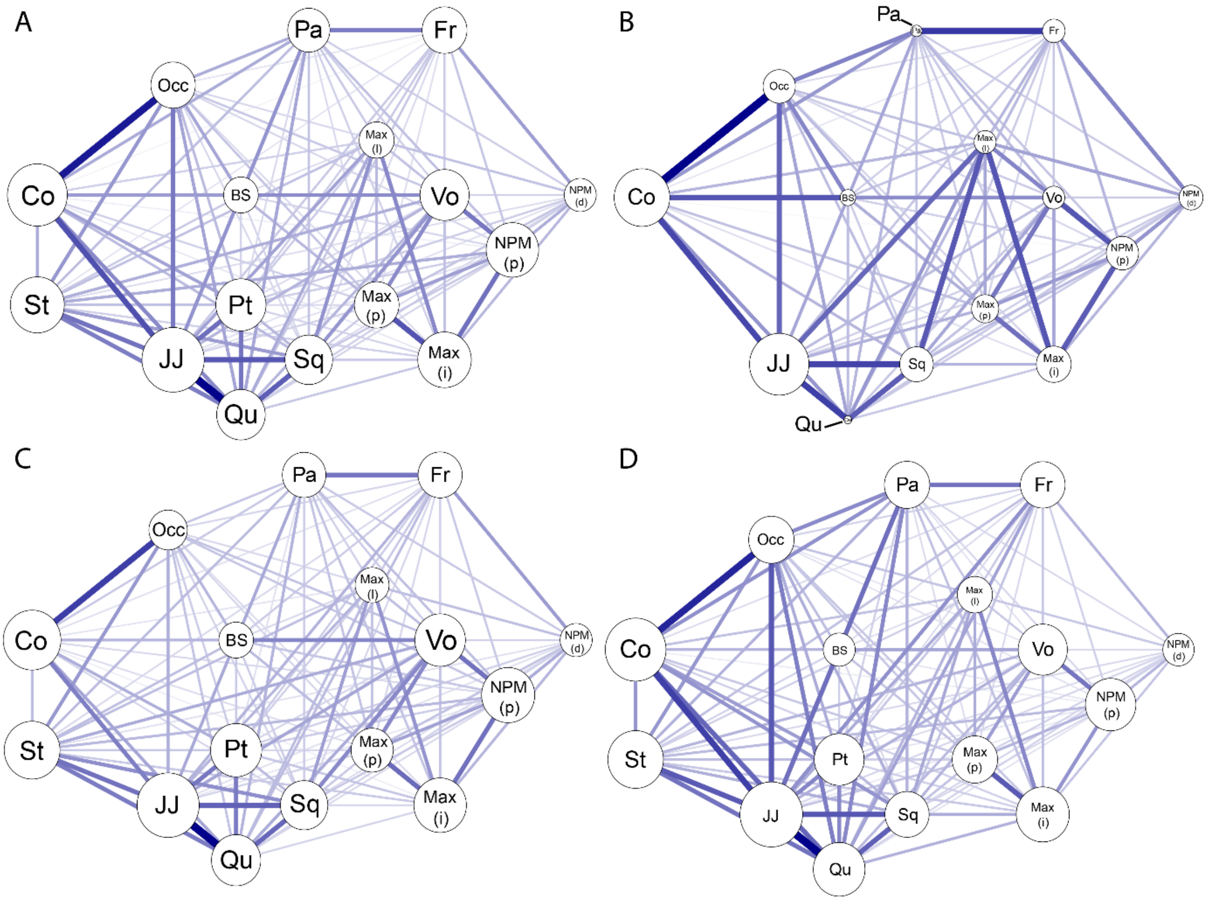

Figure S2. Network graphs displaying results from EMMLi analyses using (A) 10% subsampled, (B) anatomical landmarks only, (C) allometry-corrected, and (D) phylogenetically-corrected data. Layout approximately corresponds to a skull in lateral view, with the anterior to the right. Circle size is proportional to within-module trait correlation (ρ), and line thickness is proportional to between-module trait correlation. The stapes and pterygoid are missing from the landmark-only graph, as these are not represented by any anatomical landmarks (semilandmarks only). For abbreviations see Figure S1.

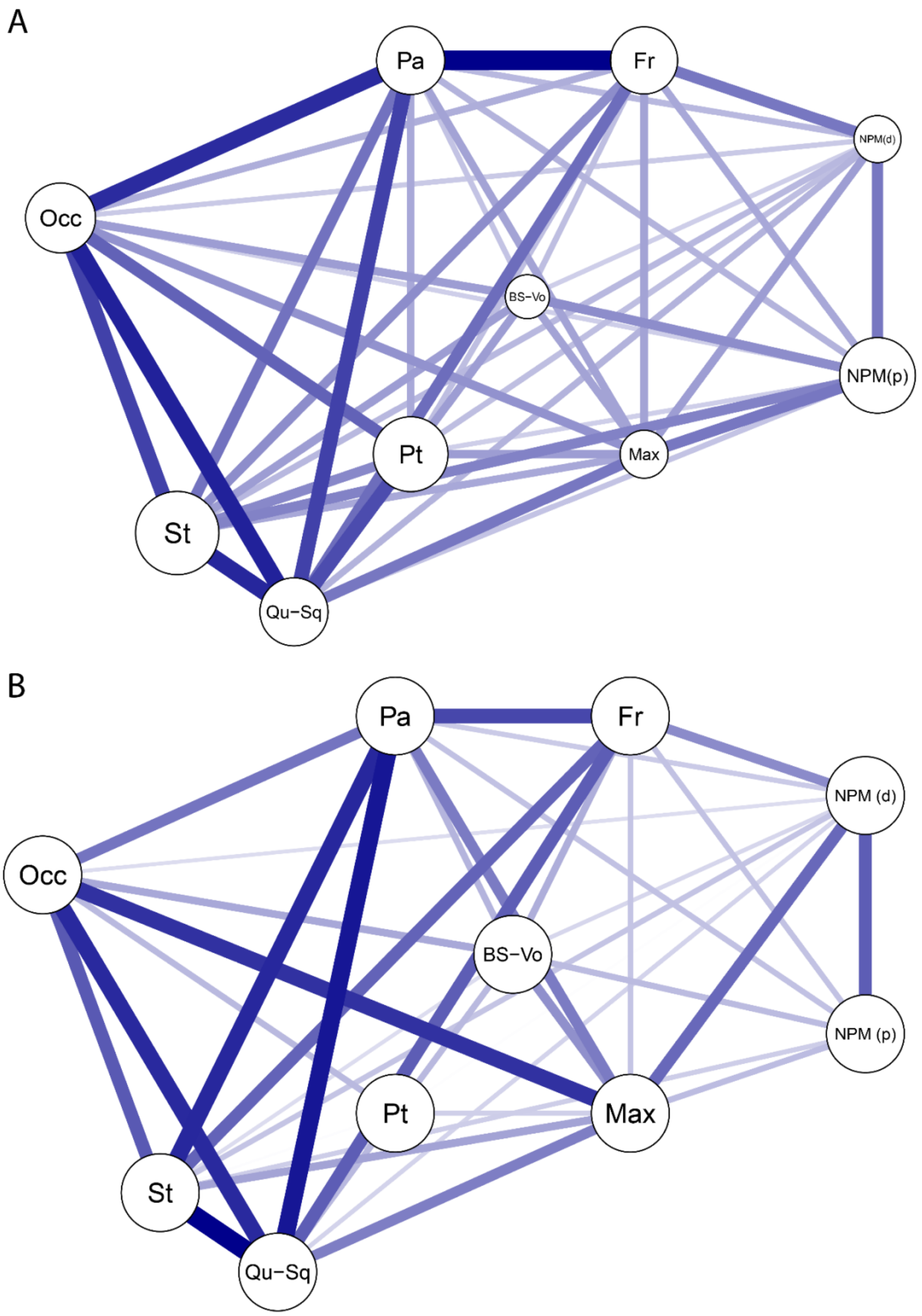

Figure S3. (A) Network graph from EMMLi analysis of the ten-module model, using phylogenetically-corrected data (see Fig.1 for module definitions). Layout approximately corresponds to a skull in lateral view, with the anterior to the right. Circle size is proportional to within-module trait correlation (ρ), and line thickness is proportional to between-module trait correlation. (B) Network graph from Covariance Ratio analysis of the ten-module model, using phylogenetically-corrected data. Line thickness is an indicator of strength of covariation (but is not proportional, as values above 0.5 are cut off for ease of visualisation).

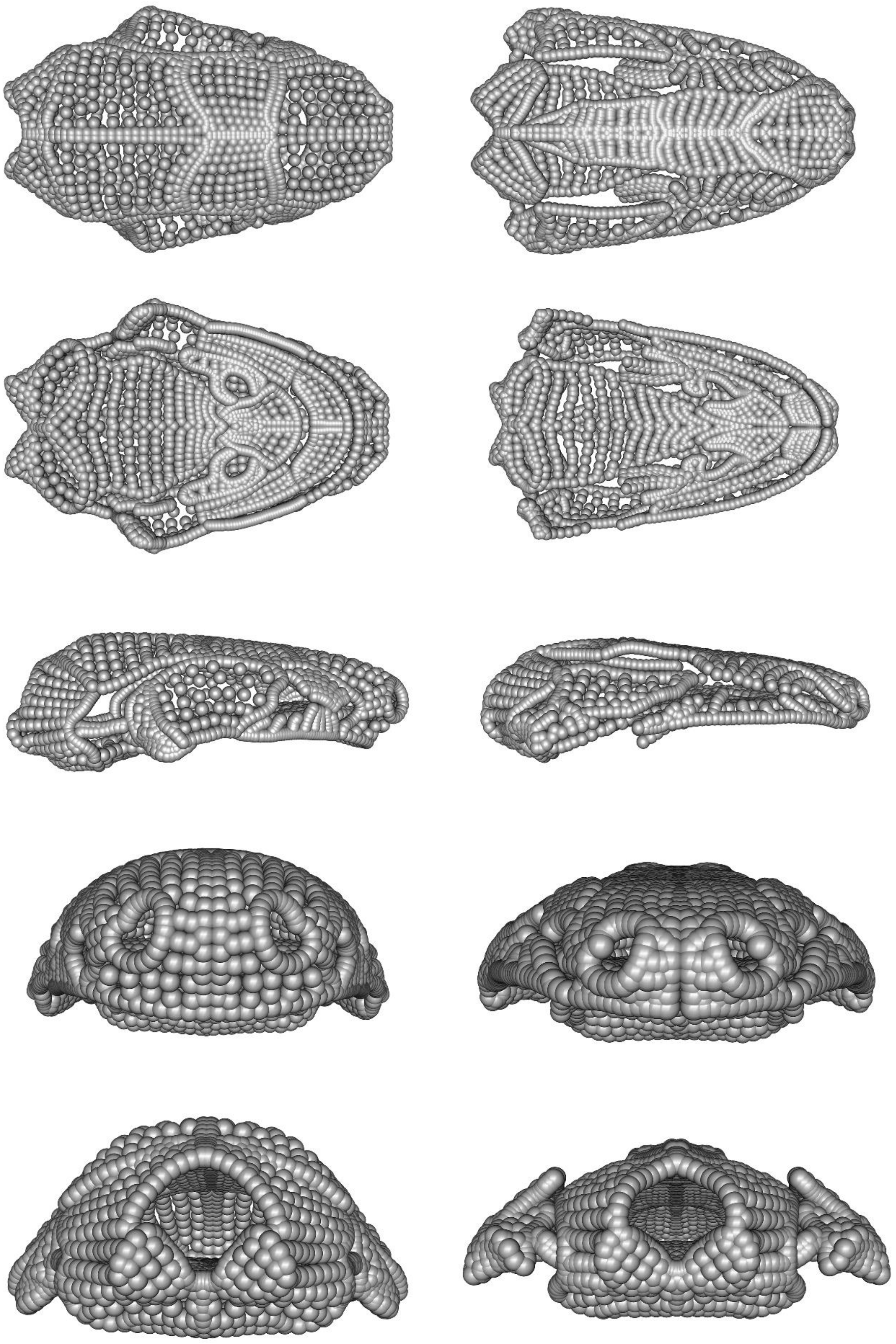

Figure S4. Reconstructed morphologies represented by positive (left) and negative (right) ends of PC1, in dorsal, ventral, lateral, anterior, and posterior views from top to bottom.

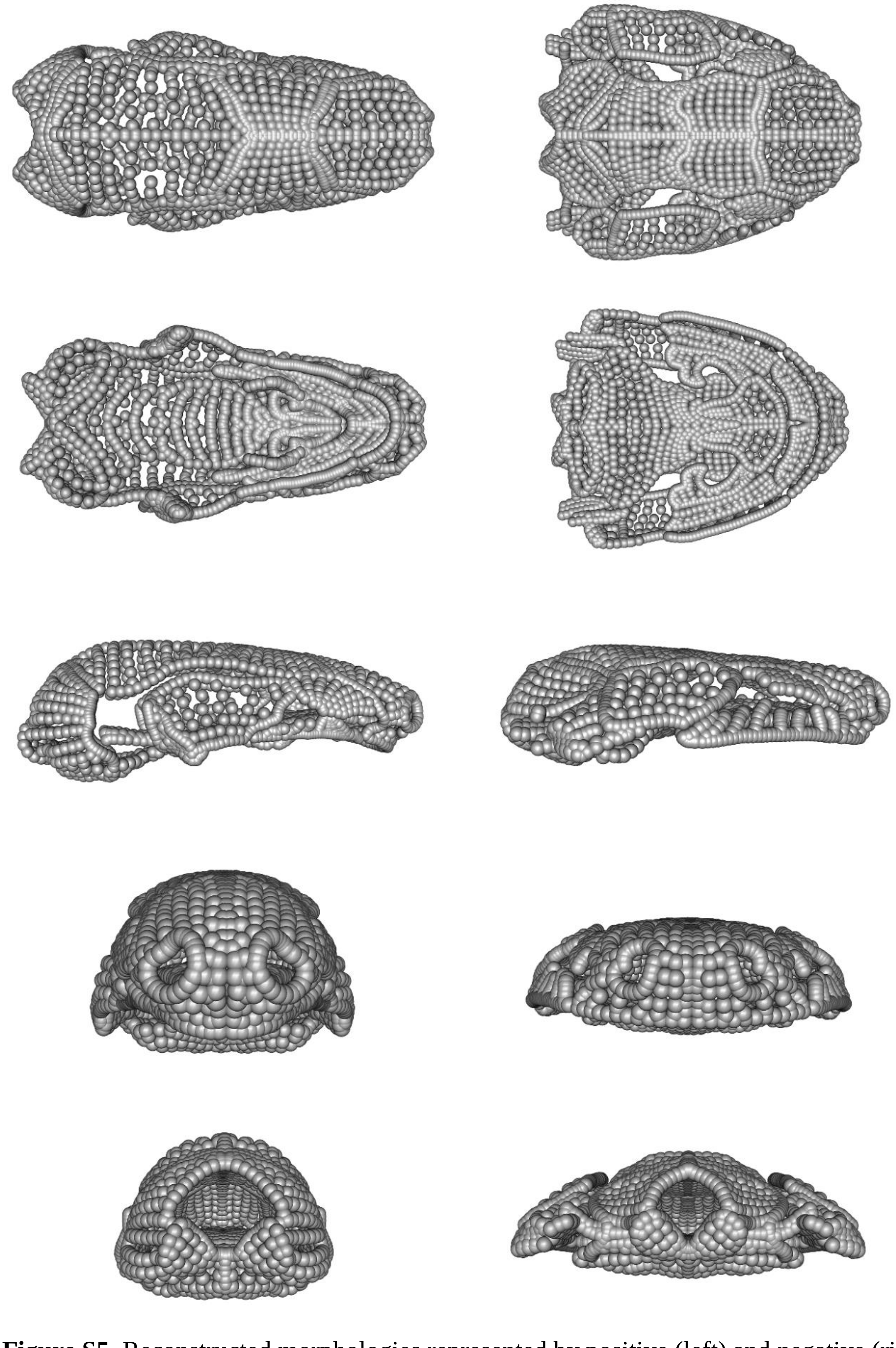

Figure S5. Reconstructed morphologies represented by positive (left) and negative (right) ends of PC2, in dorsal, ventral, lateral, anterior, and posterior views from top to bottom.

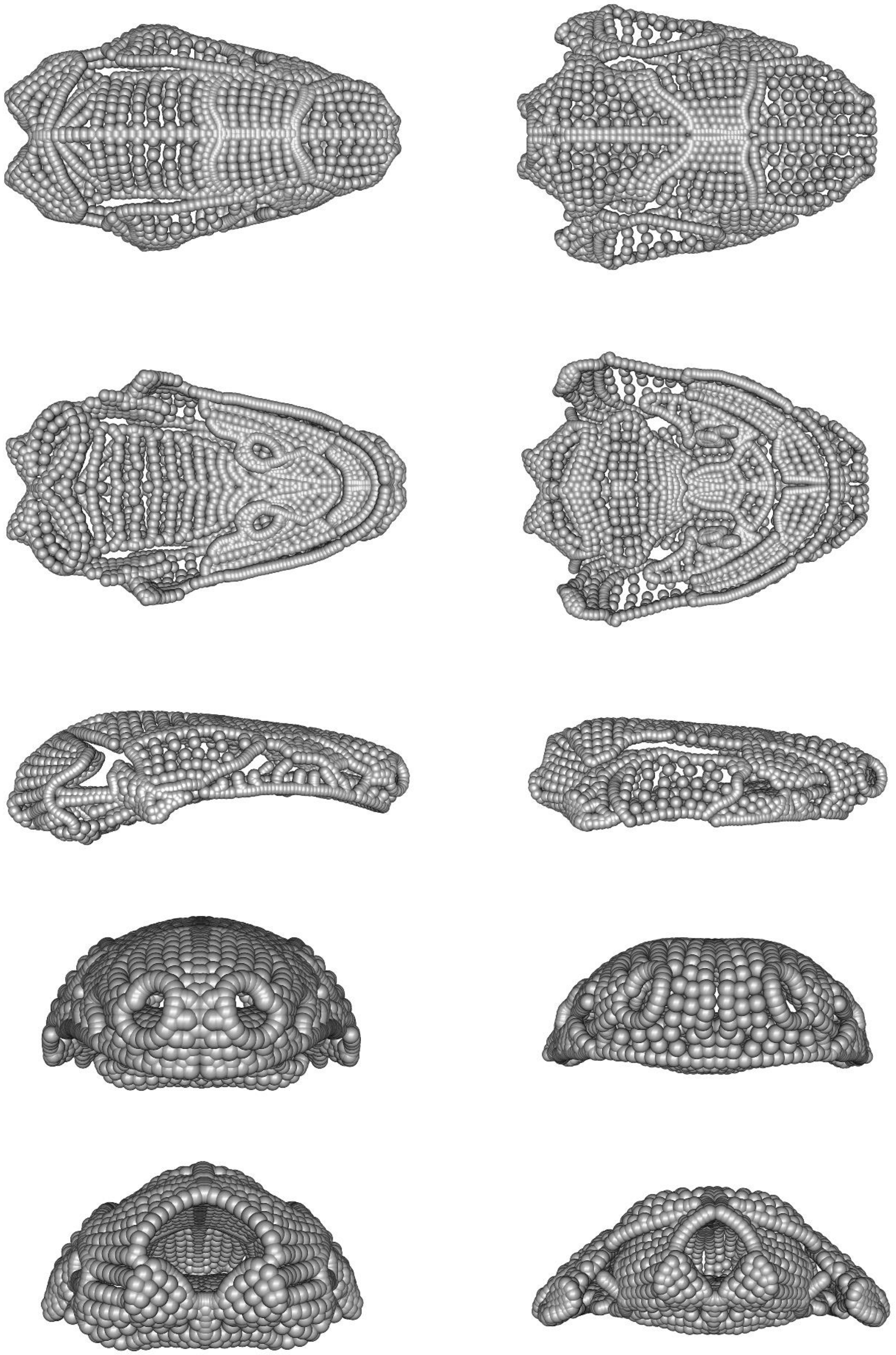

Figure S6. Reconstructed morphologies represented by positive (left) and negative (right) ends of PC3, in dorsal, ventral, lateral, anterior, and posterior views from top to bottom.

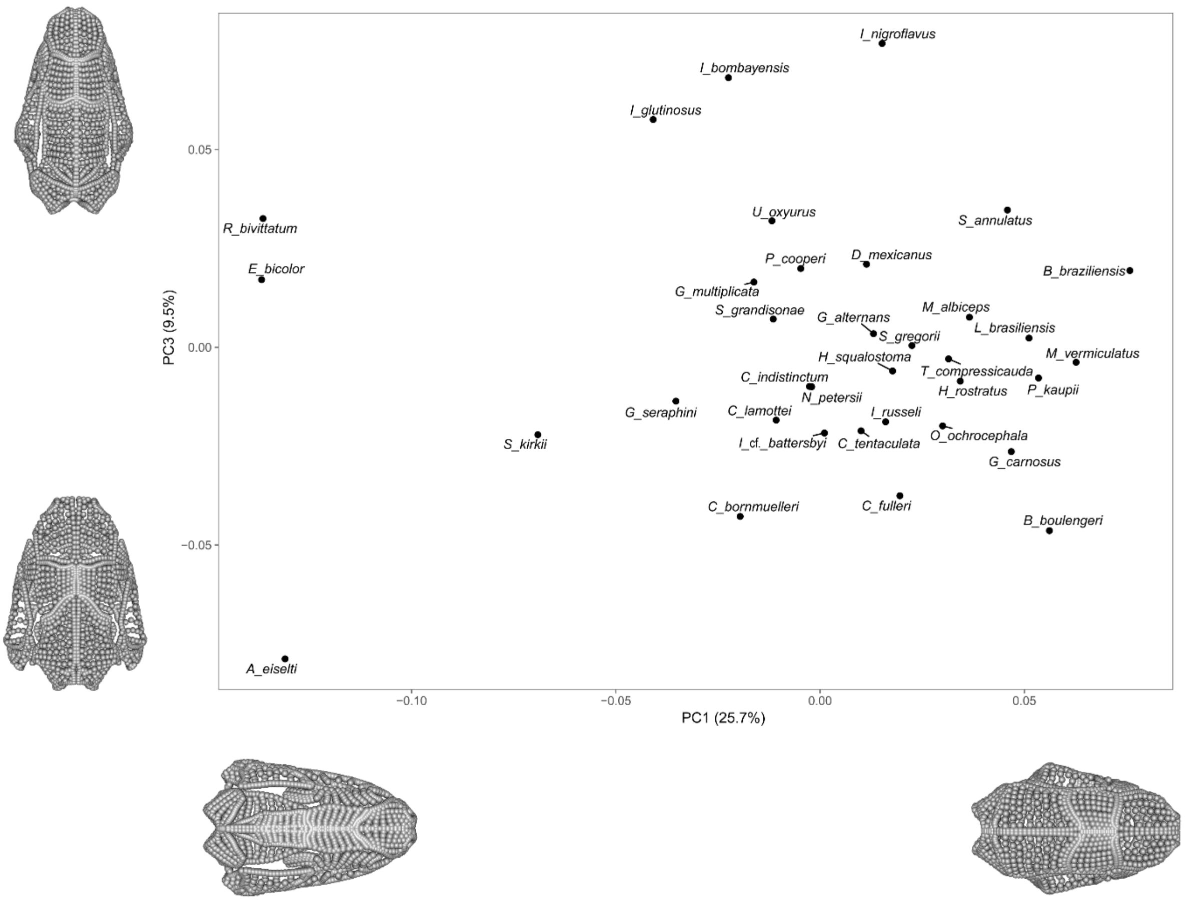

Figure S7. Morphospace of all 35 specimens based on the complete landmark and semilandmark dataset, with PC1 on the x-axis and PC3 on the y-axis. Shapes represented by the positive and negative end of each axis are displayed in dorsal view.

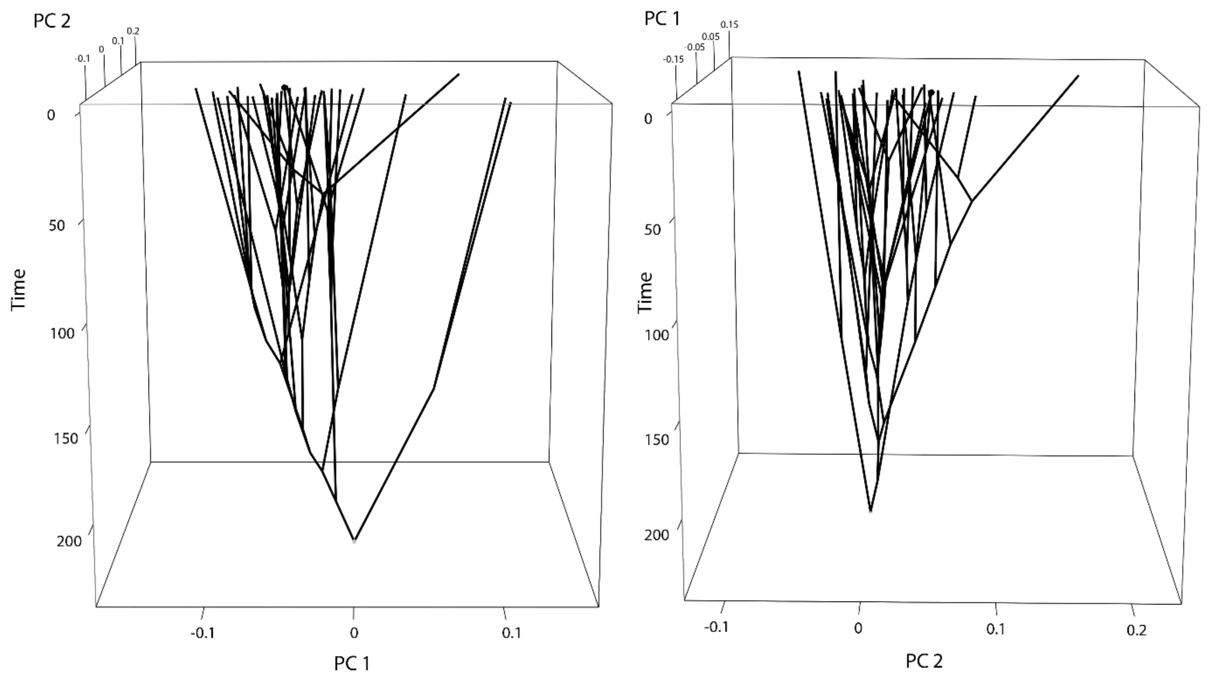

Figure S8. Phylomorphospace of all specimens in this study, with time in millions of years on the vertical axis (present day at the top).

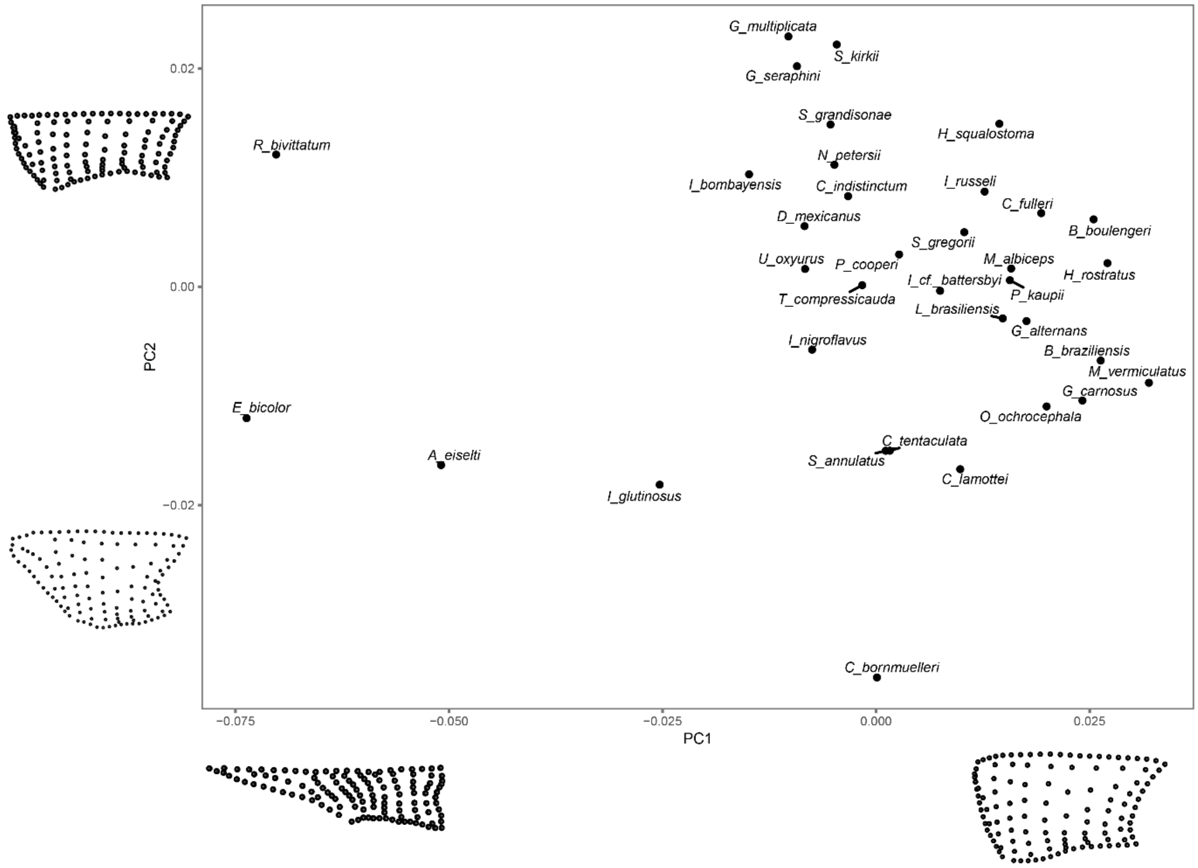

Figure S9. Morphospace of the parietal module for all specimens. This morphospace was generated from a PCA of this individual module, so axes may not align with those of the entire dataset. Shapes representing the positive and negative extremes along PC1 and PC2 are displayed in dorsal view (with anterior to the right).

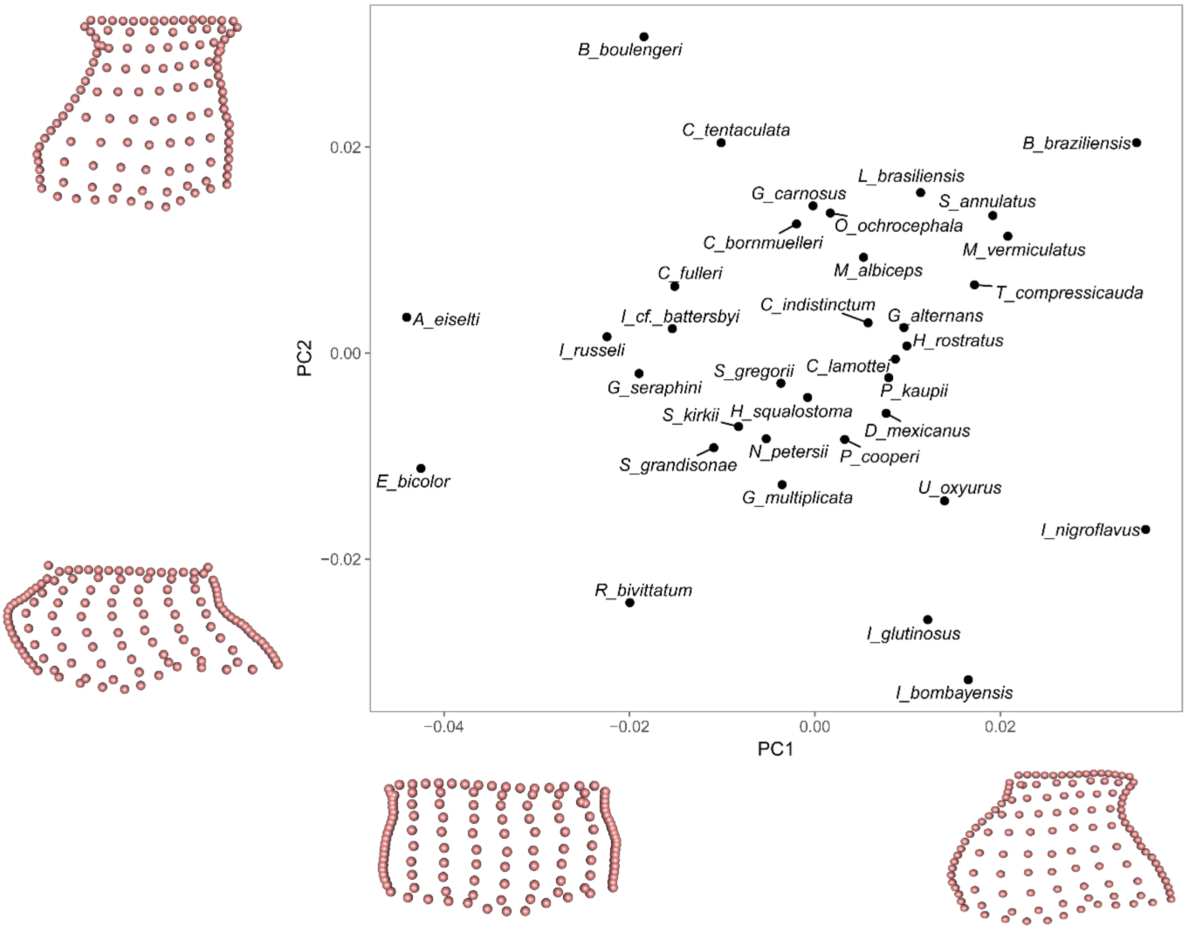

Figure S10. Morphospace of the frontal module for all specimens. This morphospace was generated from a PCA of this individual module, so axes may not align with those of the entire dataset. Shapes representing the positive and negative extremes along PC1 and PC2 are displayed in dorsal view (with anterior to the right).

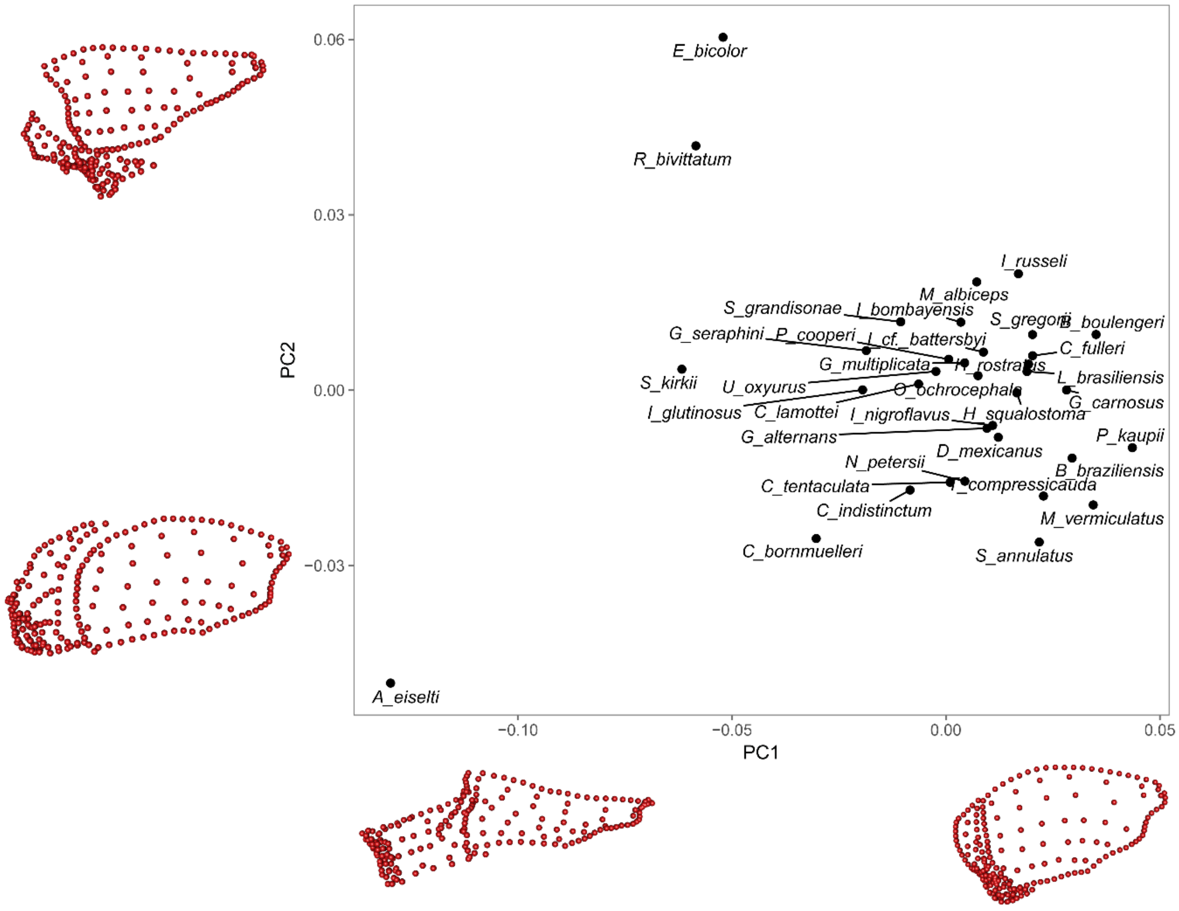

Figure S11. Morphospace of the quadrate-squamosal module for all specimens. This morphospace was generated from a PCA of this individual module, so axes may not align with those of the entire dataset. Shapes representing the positive and negative extremes along PC1 and PC2 are displayed in lateral view (with anterior to the right).

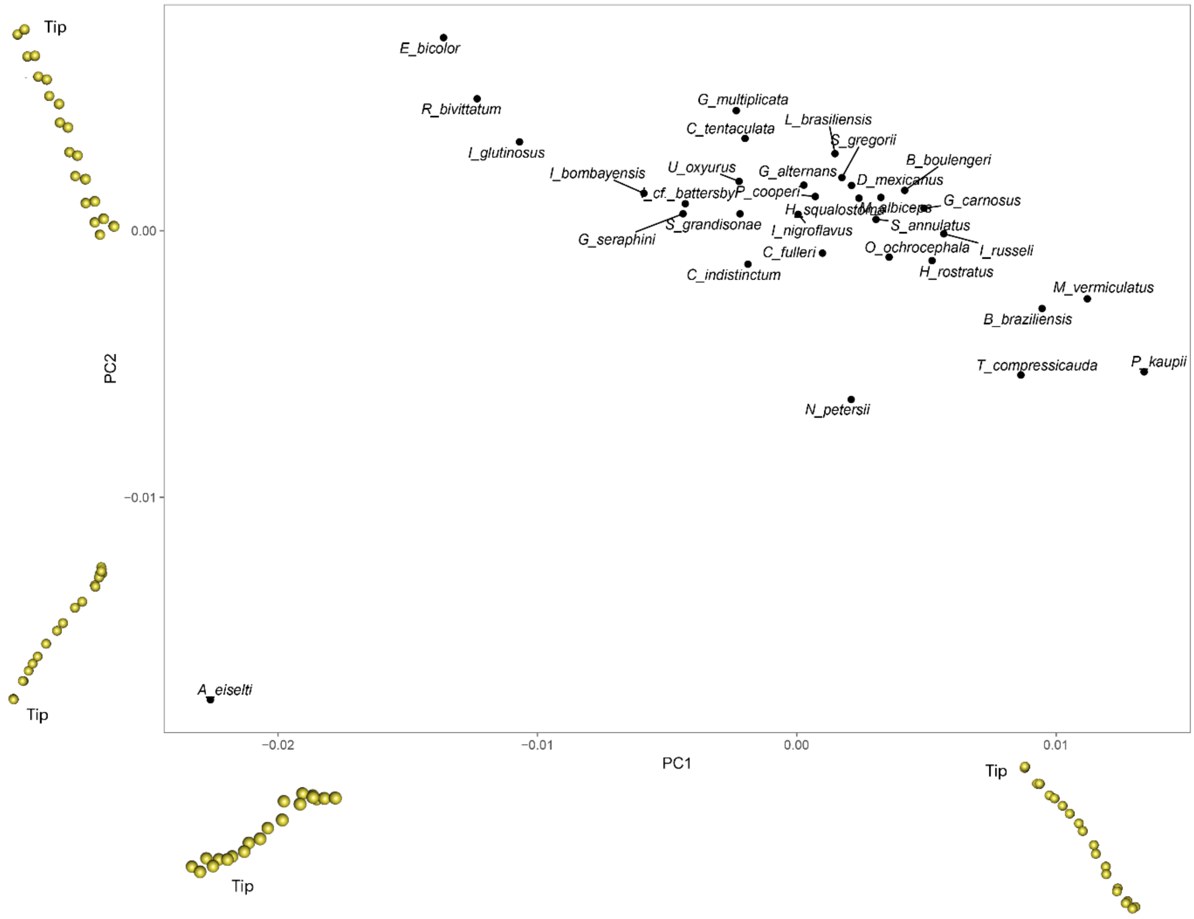

Figure S12. Morphospace of the stapes module for all specimens. This morphospace was generated from a PCA of this individual module, so axes may not align with those of the entire dataset. Shapes representing the positive and negative extremes along PC1 and PC2 are displayed in ventral view (with lateral to the left). ‘Tip’ refers to position of the lateral tip of the stapes.

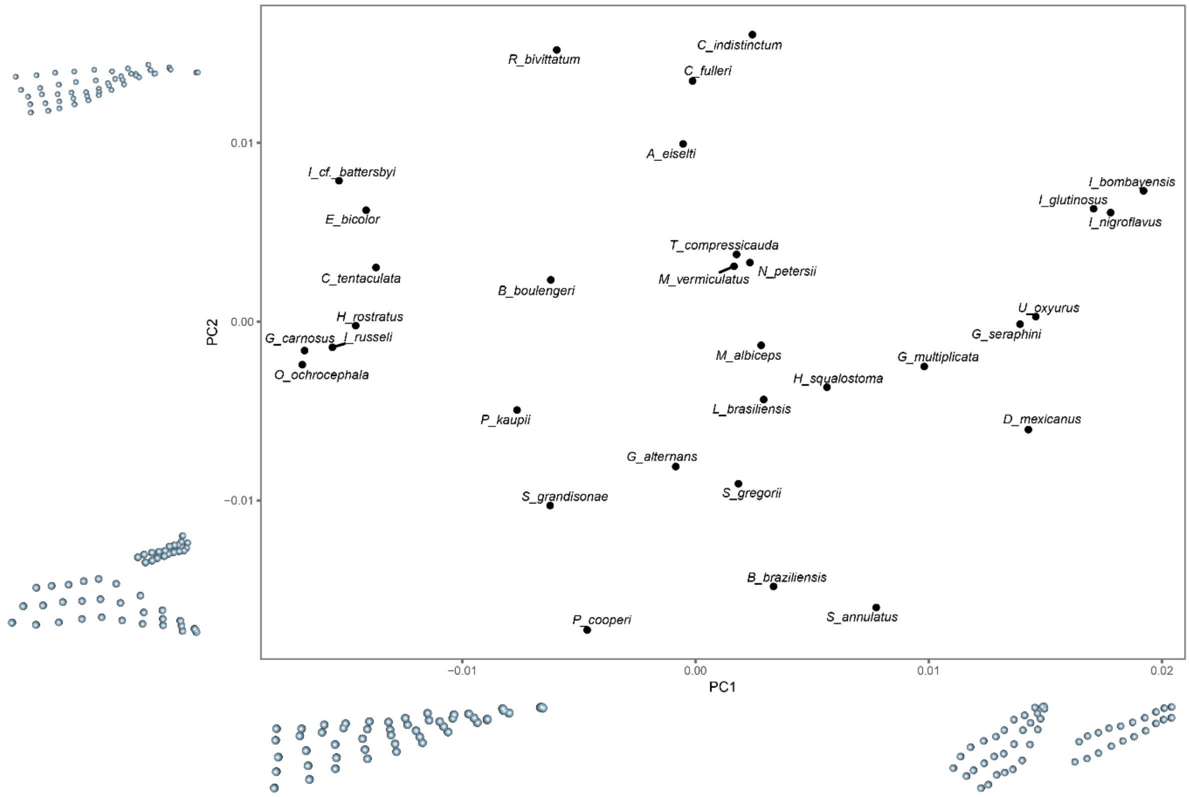

Figure S13. Morphospace of the pterygoid module for all specimens. This morphospace was generated from a PCA of this individual module, so axes may not align with those of the entire dataset. Shapes representing the positive and negative extremes along PC1 and PC2 are displayed in ventral view (with anterior to the right).

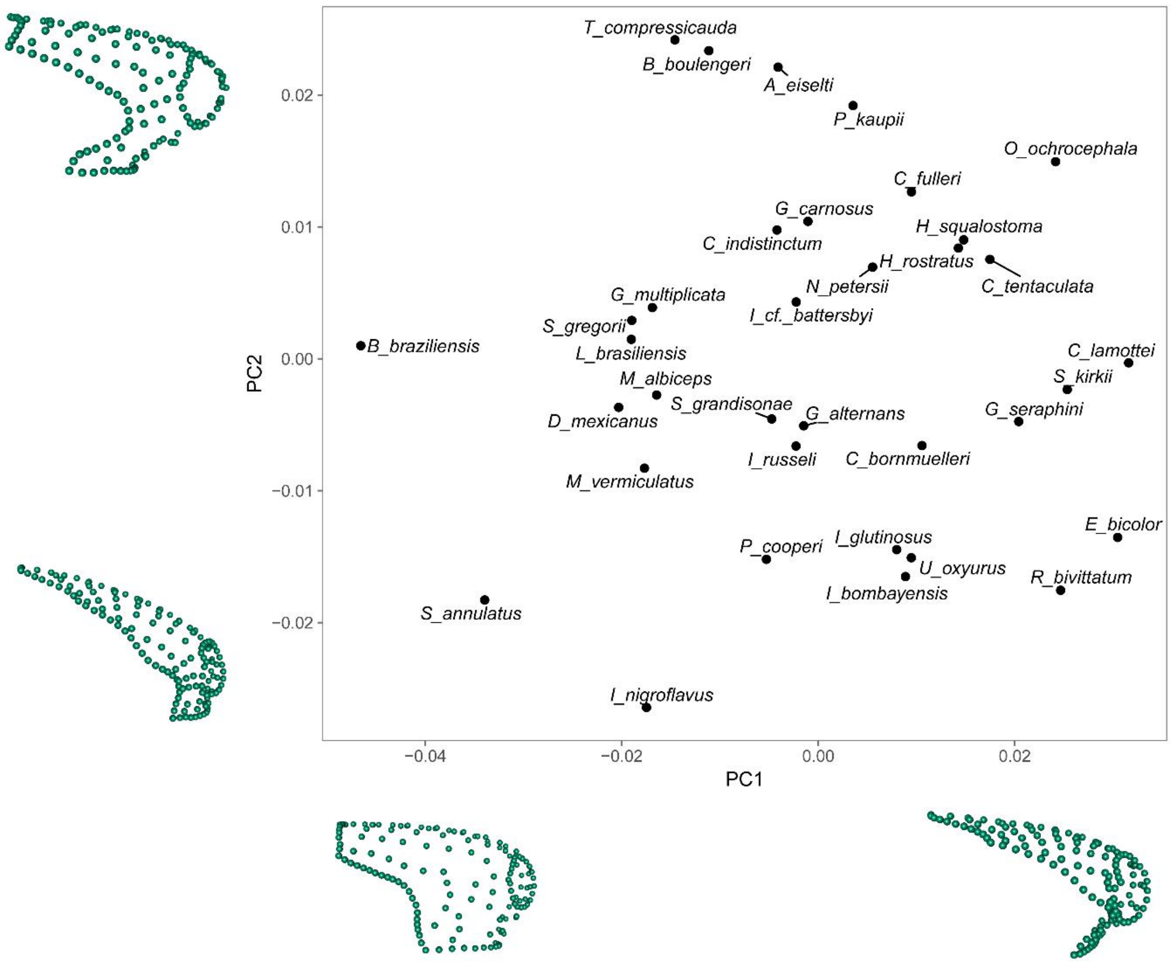

Figure S14. Morphospace of the nasopremaxilla (dorsal) module for all specimens. This morphospace was generated from a PCA of this individual module, so axes may not align with those of the entire dataset. Shapes representing the positive and negative extremes along PC1 and PC2 are displayed in lateral view (with anterior to the right).

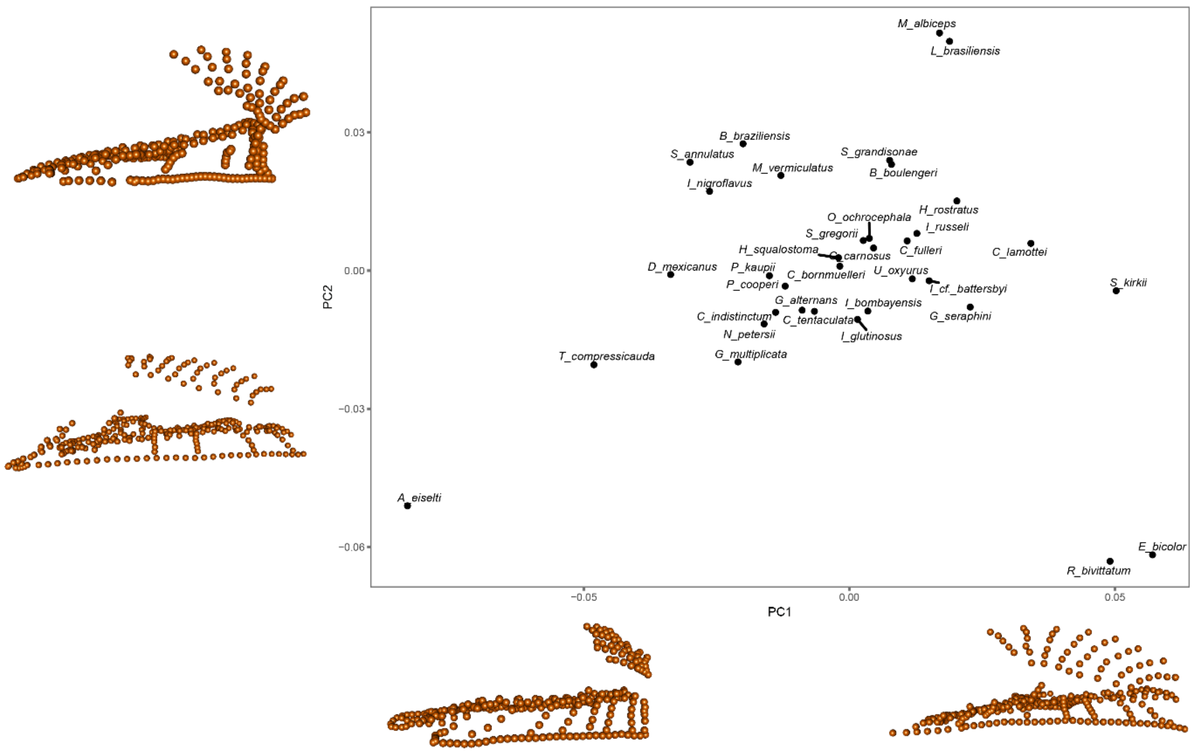

Figure S15. Morphospace of the maxillopalatine module for all specimens. This morphospace was generated from a PCA of this individual module, so axes may not align with those of the entire dataset. Shapes representing the positive and negative extremes along PC1 and PC2 are displayed in lateral view (with anterior to the right).

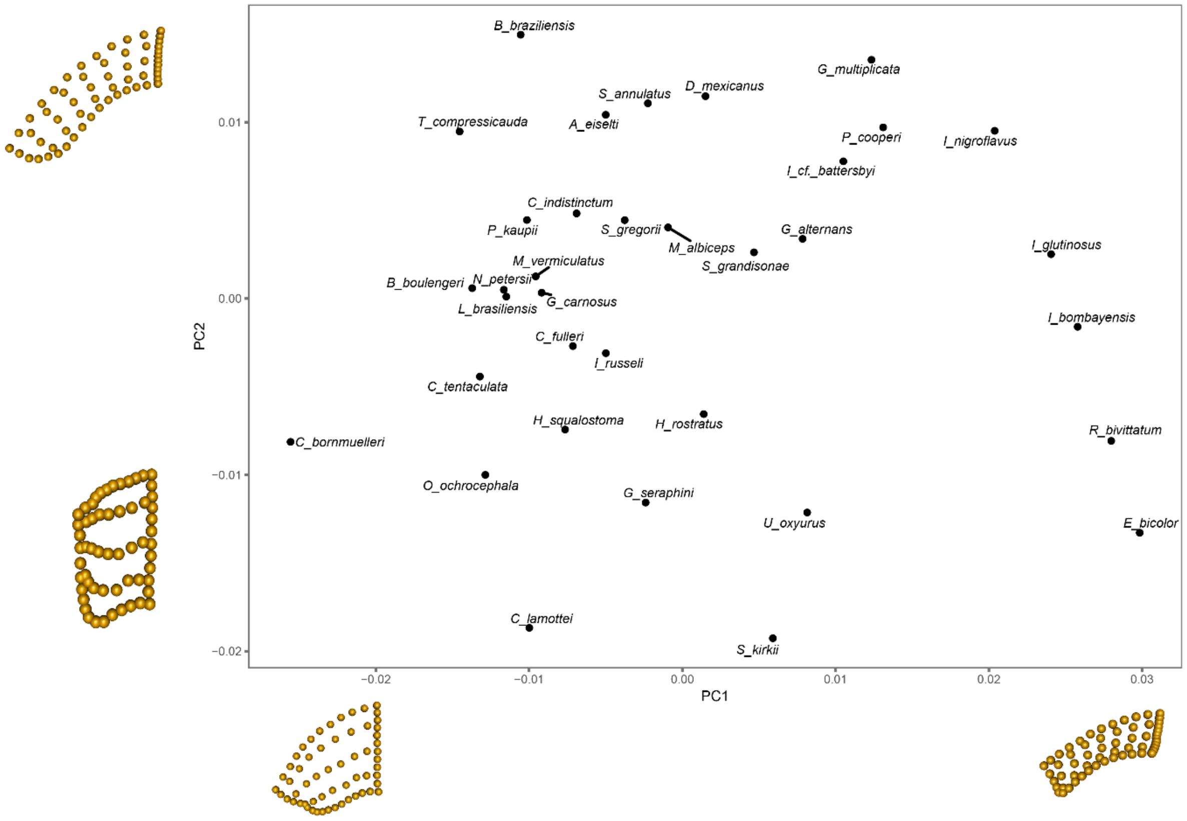

Figure S16. Morphospace of the nasopremaxilla (palatal) module for all specimens. This morphospace was generated from a PCA of this individual module, so axes may not align with those of the entire dataset. Shapes representing the positive and negative extremes along PC1 and PC2 are displayed in ventral view (with medial to the right).

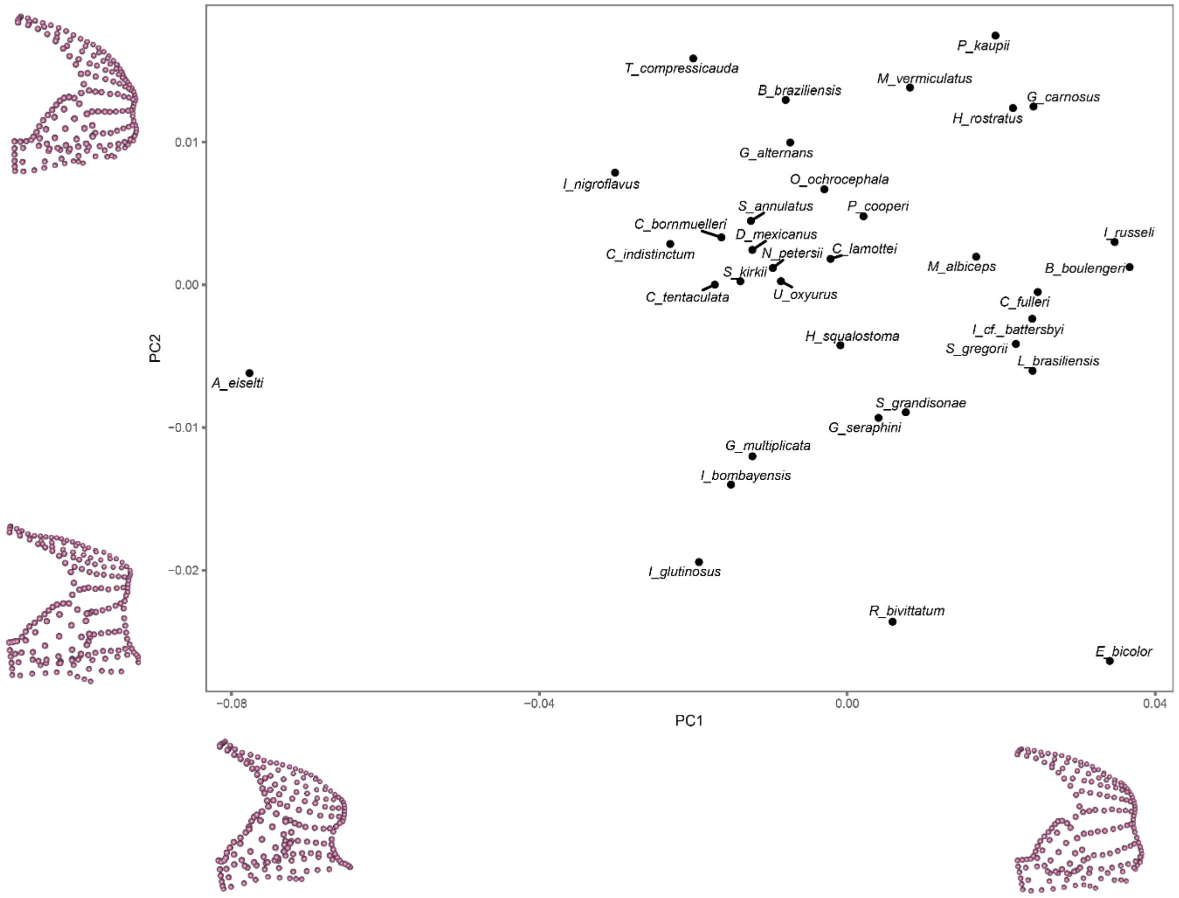

Figure S17. Morphospace of the occipital module for all specimens. This morphospace was generated from a PCA of this individual module, so axes may not align with those of the entire dataset. Shapes representing the positive and negative extremes along PC1 and PC2 are displayed in posterior view (with lateral to the right).

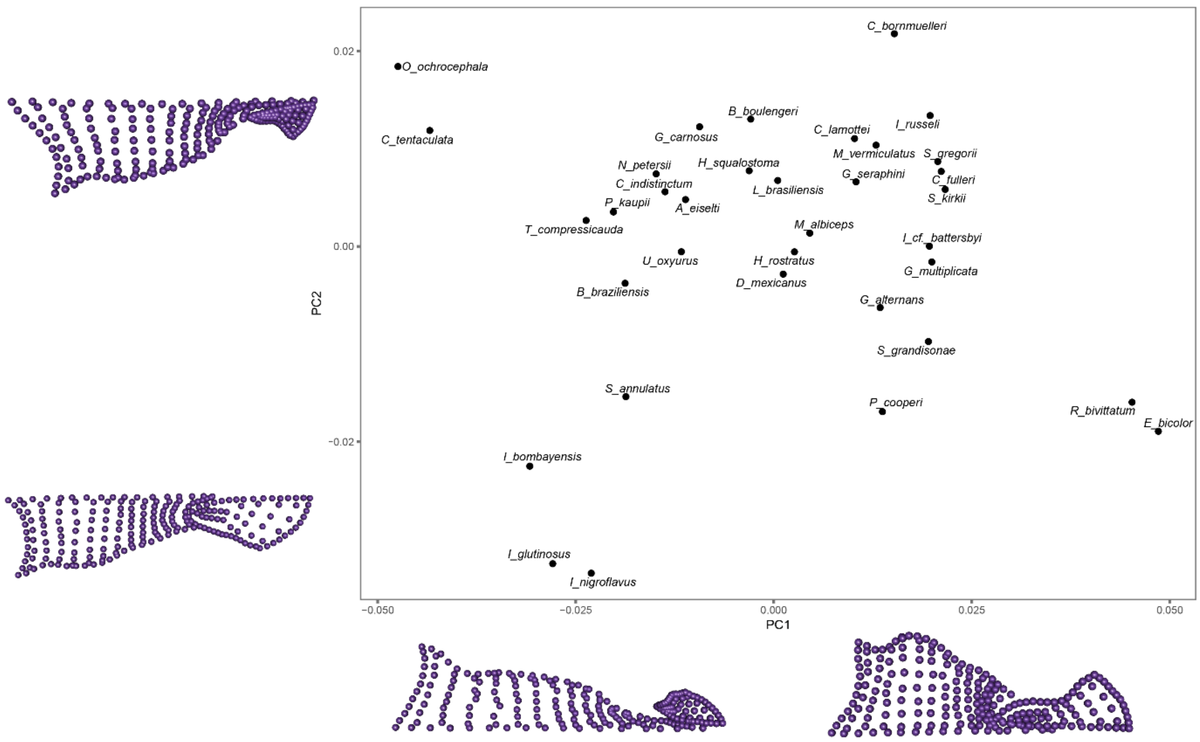

Figure S18. Morphospace of the ventral os basale-vomer module for all specimens. This morphospace was generated from a PCA of this individual module, so axes may not align with those of the entire dataset. Shapes representing the positive and negative extremes along PC1 and PC2 are displayed in ventral view (with anterior to the right).

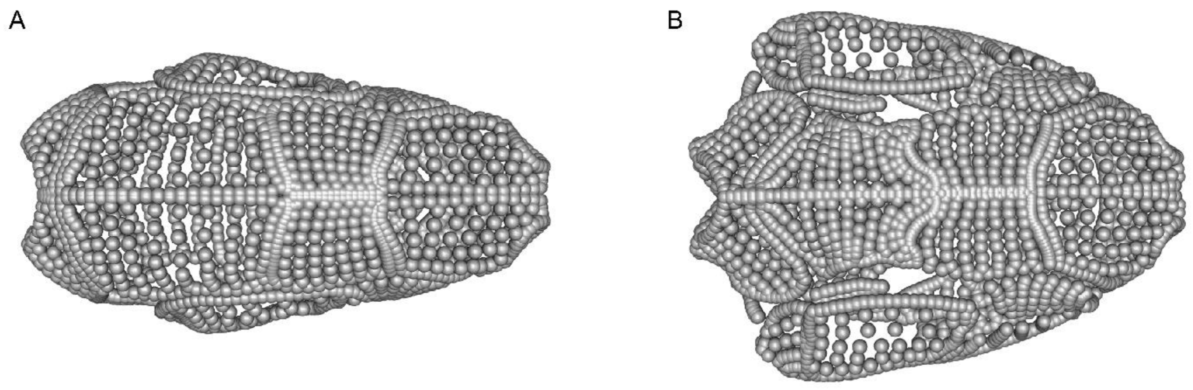

Figure S19. Whole cranial morphology at (A) minimum and (B) maximum size.

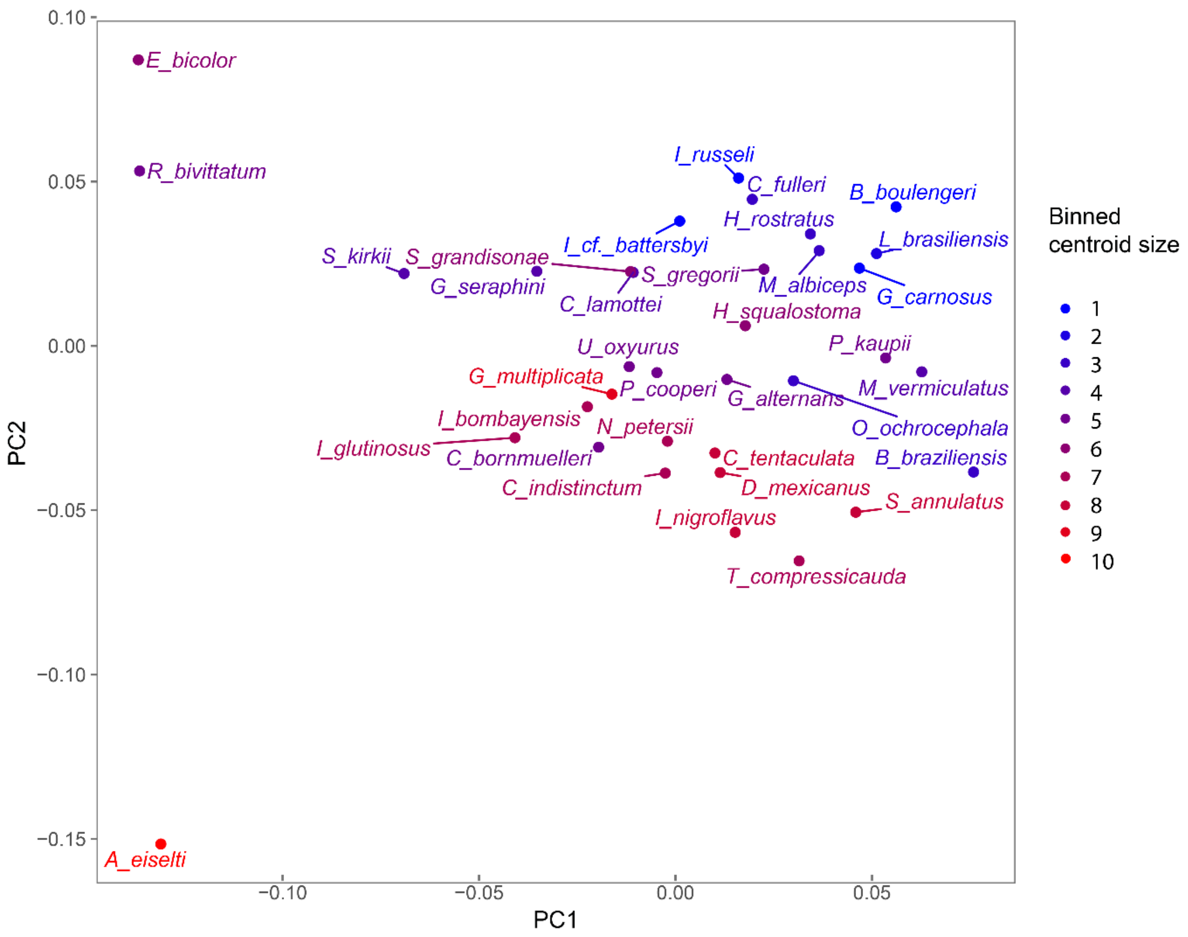

Figure S20. Distribution of specimens in morphospace, colour-coded by centroid size, binned into 10 bins of equal size.

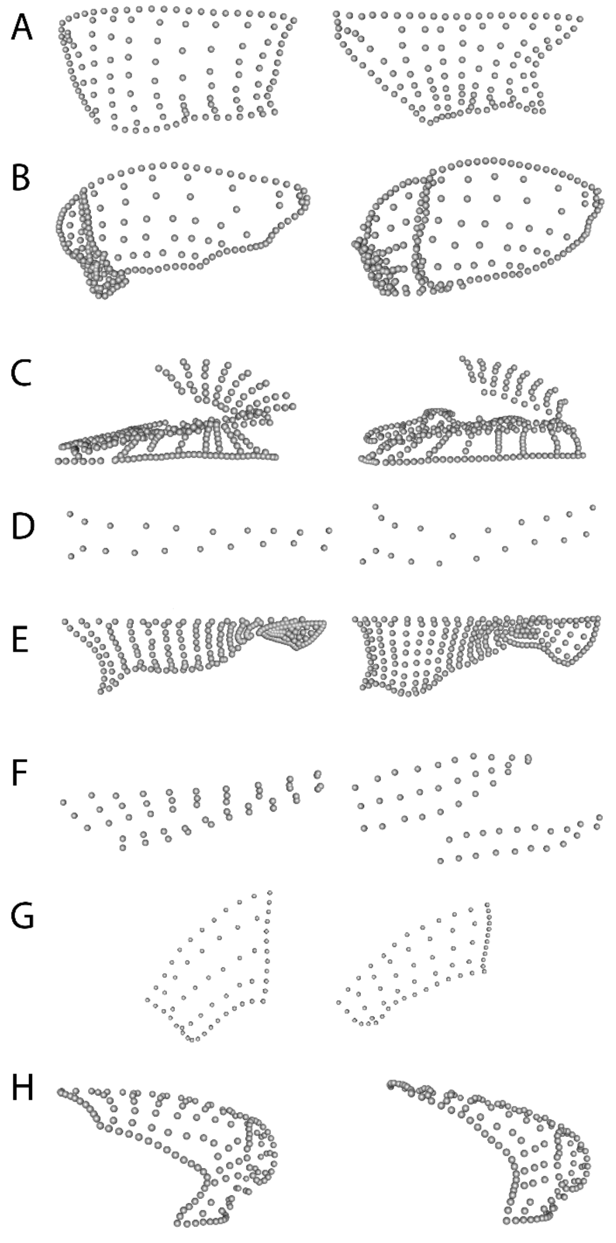

Figure S21. Module morphologies at minimum (left) and maximum (right) size. Allometry was investigated using global Procrustes alignment. The occipital module was the most influenced by allometry and can be seen in Figure S22. Remaining modules are in descending order of strength of allometry: (A) Parietal, (B) Quadrate-squamosal, (C) Maxillopalatine, (D) Stapes, (E) Ventral os basale-vomer, (F) Pterygoid, (G) Nasopremaxilla (palatal surface), (H) Nasopremaxilla (dorsal surface). The frontal module is excluded because it did not display significant allometric signal.

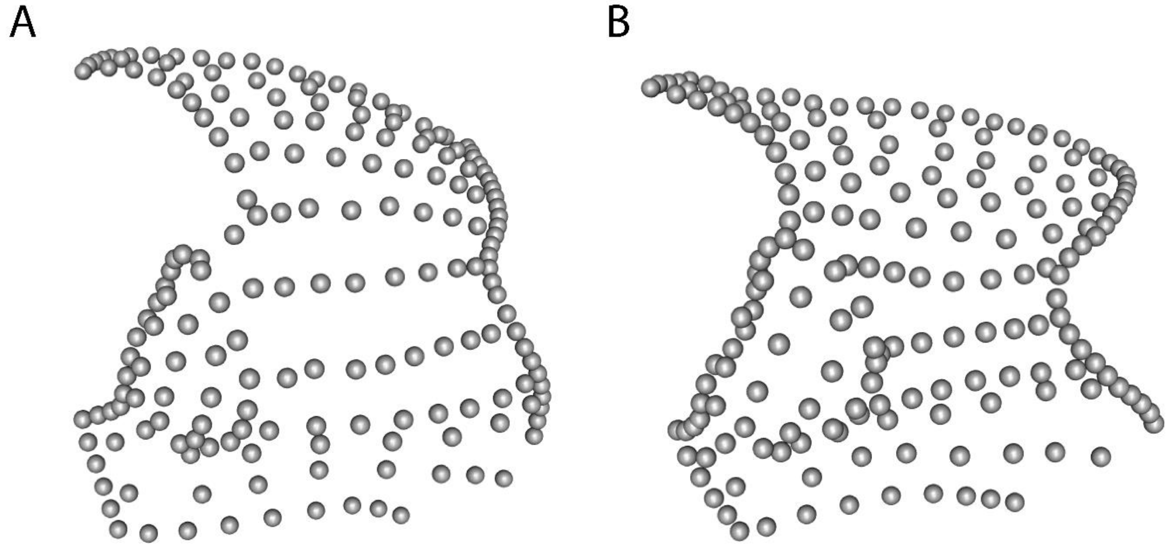

Figure S22. The occipital module morphology (posterior view), at (A) minimum and (B) maximum size. Note the 'bulging' of this module dorsally at minimum size, in the position of the otic capsule, suggesting smaller species have relatively larger otic capsules.

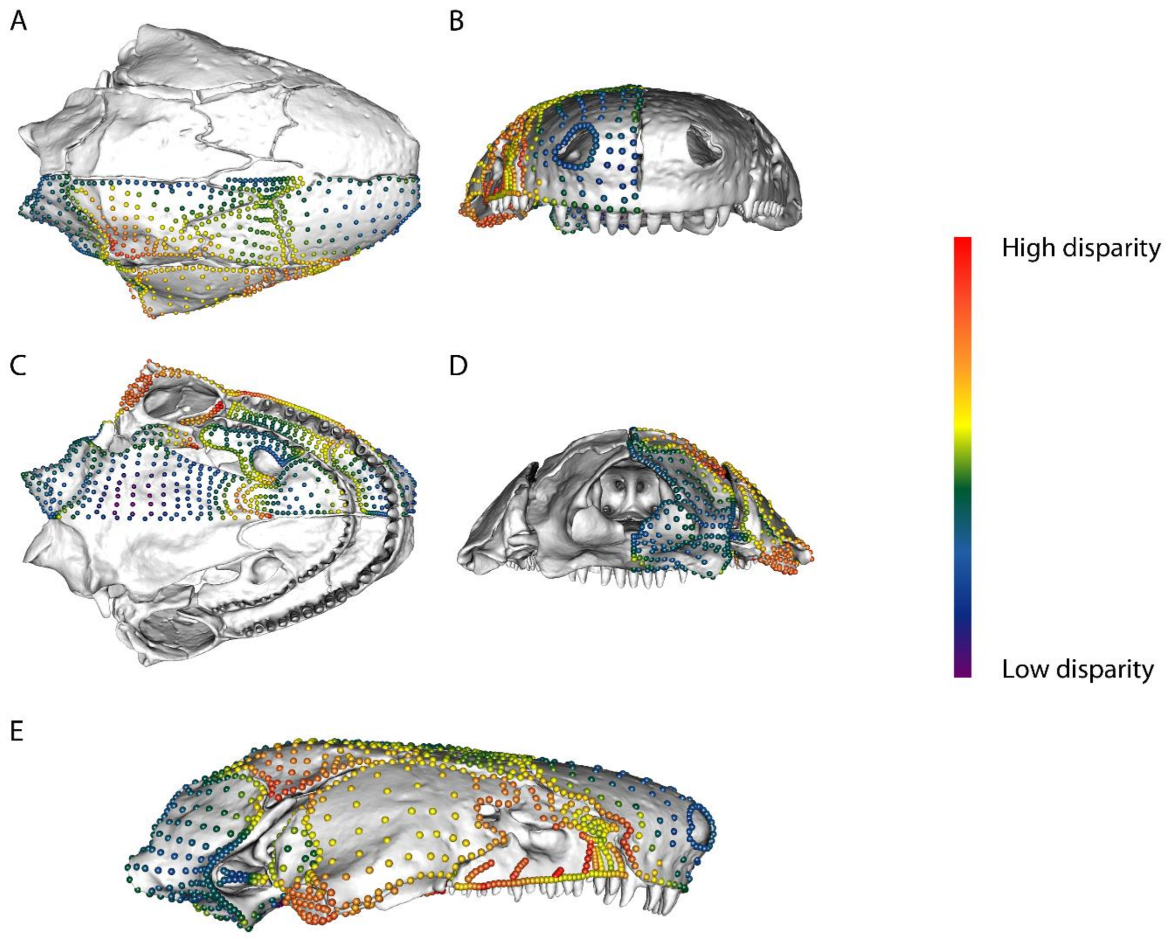

Figure S23. Landmarks and semilandmarks displayed on *Siphonops annulatus* and colour coded by magnitude of disparity, measured as Procrustes variance, from low (purple) to high (red), in (A) dorsal, (B) anterior, (C) ventral, (D) posterior, and (E) lateral aspect.

Table S1. Results of EMMLi analysis for trait correlations (ρ) within and between 16 cranial regions.

	Frontal	Parietal	Nasopremaxilla (dorsal)	Maxillopalatine (lateral)	Os basale (occipital)	Occipital condyle	Quadrate (jaw joint)	Squamosal	Quadrate (lateral)	Os basale (ventral)	Nasopremaxilla (palatal)	Vomer	Maxillopalatine (interdental plate)	Maxillopalatine (palatine shelf)	Pterygoid	Stapes
Frontal	0.70															
Parietal	0.39	0.67														
Nasopremaxilla (dorsal)	0.31	0.16	0.50													
Maxillopalatine (lateral)	0.17	0.17	0.20	0.55												
Os basale (occipital)	0.09	0.26	0.09	0.12	0.70											
Occipital condyle	0.04	0.27	0.08	0.15	0.72	0.94										
Quadrate (jaw joint)	0.17	0.34	0.10	0.28	0.42	0.54	0.97									
Squamosal	0.12	0.20	0.11	0.33	0.12	0.22	0.53	0.74								
Quadrate (lateral)	0.18	0.28	0.14	0.26	0.29	0.37	0.81	0.50	0.77							
Os basale (ventral)	0.15	0.12	0.10	0.11	0.29	0.32	0.14	0.13	0.14	0.54						
Nasopremaxilla (palatal)	0.18	0.20	0.26	0.20	0.13	0.06	0.10	0.17	0.13	0.13	0.83					
Vomer	0.14	0.15	0.15	0.18	0.17	0.12	0.23	0.33	0.23	0.35	0.44	0.77				
Maxillopalatine (interdental	0.16	0.15	0.23	0.38	0.12	0.08	0.16	0.16	0.18	0.11	0.45	0.20	0.85			

plate)																
Maxillopalatine (palatine shelf)	0.11	0.12	0.18	0.23	0.18	0.15	0.14	0.11	0.16	0.22	0.30	0.36	0.54	0.70		
Pterygoid	0.16	0.12	0.15	0.16	0.21	0.29	0.45	0.22	0.43	0.13	0.16	0.12	0.14	0.07	0.80	
Stapes	0.09	0.22	0.11	0.19	0.32	0.26	0.46	0.32	0.42	0.17	0.25	0.25	0.15	0.12	0.19	0.86

Table S2. Results of EMMLi analysis for trait correlations (ρ) within and between 16 cranial regions, for the 10% subsampled analyses, averaged over 100 iterations.

	Frontal	Parietal	Nasopremaxilla (dorsal)	Maxillopalatine (lateral)	Os basale (occipital)	Occipital condyle	Quadrate (jaw joint)	Squamosal	Quadrate (lateral)	Os basale (ventral)	Nasopremaxilla (palatal)	Vomer	Maxillopalatine (interdental plate)	Maxillopalatine (palatine shelf)	Pterygoid	Stapes
Frontal	0.69															
Parietal	0.40	0.66														
Nasopremaxilla (dorsal)	0.31	0.16	0.50													
Maxillopalatine (lateral)	0.17	0.17	0.19	0.54												
Os basale (occipital)	0.09	0.26	0.09	0.12	0.70											
Occipital condyle	0.04	0.26	0.08	0.15	0.71	0.94										
Quadrate (jaw joint)	0.17	0.34	0.10	0.27	0.42	0.55	0.97									
Squamosal	0.12	0.21	0.11	0.33	0.12	0.22	0.52	0.74								
Quadrate (lateral)	0.17	0.28	0.14	0.25	0.29	0.37	0.80	0.50	0.76							
Os basale (ventral)	0.15	0.12	0.10	0.10	0.29	0.31	0.14	0.13	0.14	0.54						
Nasopremaxilla (palatal)	0.18	0.20	0.25	0.20	0.13	0.05	0.10	0.17	0.13	0.14	0.83					

Vomer	0.14	0.16	0.15	0.18	0.18	0.12	0.23	0.33	0.23	0.35	0.44	0.77				
Maxillopalatine (interdental plate)	0.15	0.15	0.22	0.38	0.12	0.08	0.16	0.16	0.18	0.11	0.43	0.20	0.84			
Maxillopalatine (palatine shelf)	0.11	0.12	0.18	0.23	0.18	0.15	0.14	0.11	0.16	0.22	0.30	0.36	0.53	0.69		
Pterygoid	0.16	0.12	0.15	0.16	0.21	0.29	0.45	0.22	0.43	0.13	0.16	0.12	0.14	0.07	0.78	
Stapes	0.09	0.21	0.11	0.19	0.32	0.26	0.45	0.31	0.41	0.17	0.26	0.26	0.15	0.12	0.19	0.85

Table S3. Results of EMMLi analysis for trait correlations (ρ) within and between 16 cranial regions, for the anatomical landmark only dataset. The stapes and pterygoid regions are excluded from this analysis as they are only represented by semilandmarks.

	Frontal	Parietal	Nasopremaxilla (dorsal)	Maxillopalatine (lateral)	Os basale (occipital)	Occipital condyle	Quadrate (jaw joint)	Squamosal	Quadrate (lateral)	Os basale (ventral)	Nasopremaxilla (palatal)	Vomer	Maxillopalatine (interdental plate)	Maxillopalatine (palatine shelf)
Frontal	0.35													
Parietal	0.54	0.17												
Nasopremaxilla (dorsal)	0.33	0.14	0.37											
Maxillopalatine (lateral)	0.2	0.15	0.25	0.34										
Os basale (occipital)	0.1	0.34	0.09	0.17	0.5									
Occipital condyle	0.07	0.28	0.07	0.2	0.69	0.86								
Quadrate (jaw joint)	0.16	0.24	0.13	0.41	0.42	0.5	0.92							
Squamosal	0.14	0.16	0.13	0.45	0.18	0.2	0.51	0.52						
Quadrate (lateral)	0.19	0.2	0.15	0.28	0.25	0.29	0.52	0.44	0.12					
Os basale (ventral)	0.13	0.19	0.1	0.16	0.33	0.46	0.18	0.14	0.18	0.24				
Nasopremaxilla (palatal)	0.2	0.16	0.29	0.33	0.11	0.02	0.12	0.18	0.14	0.13	0.5			
Vomer	0.15	0.19	0.15	0.21	0.14	0.12	0.18	0.24	0.15	0.31	0.46	0.34		
Maxillopalatine (interdental plate)	0.16	0.14	0.2	0.46	0.12	0.09	0.21	0.21	0.15	0.13	0.44	0.24	0.54	
Maxillopalatine (palatine shelf)	0.12	0.14	0.14	0.24	0.18	0.17	0.21	0.14	0.17	0.22	0.27	0.37	0.39	0.42

Table S4. Results of EMMLi analysis for trait correlations (ρ) within and between 16 cranial regions, for the allometry-corrected data. Analyses were conducted on all 16 cranial regions.

	Frontal	Parietal	Nasopremaxilla (dorsal)	Maxillopalatine (lateral)	Os basale (occipital)	Occipital condyle	Quadrate (jaw joint)	Squamosal	Quadrate (lateral)	Os basale (ventral)	Nasopremaxilla (palatal)	Vomer	Maxillopalatine (interdental plate)	Maxillopalatine (palatine shelf)	Pterygoid	Stapes
Frontal	0.69															
Parietal	0.43	0.68														
Nasopremaxilla (dorsal)	0.31	0.17	0.5													
Maxillopalatine (lateral)	0.17	0.15	0.2	0.53												
Os basale (occipital)	0.13	0.18	0.12	0.12	0.6											
Occipital condyle	0.1	0.17	0.11	0.09	0.6	0.9										
Quadrate (jaw joint)	0.24	0.27	0.1	0.23	0.25	0.4	0.97									
Squamosal	0.13	0.16	0.11	0.3	0.14	0.13	0.49	0.74								
Quadrate (lateral)	0.21	0.24	0.14	0.21	0.19	0.28	0.79	0.47	0.77							
Os basale (ventral)	0.16	0.12	0.12	0.12	0.23	0.23	0.16	0.16	0.17	0.54						
Nasopremaxilla (palatal)	0.17	0.2	0.26	0.19	0.18	0.12	0.08	0.17	0.12	0.18	0.83					
Vomer	0.13	0.16	0.15	0.2	0.19	0.16	0.28	0.37	0.26	0.38	0.44	0.79				
Maxillopalatine (interdental plate)	0.15	0.19	0.23	0.33	0.11	0.06	0.13	0.13	0.14	0.11	0.44	0.19	0.82			
Maxillopalatine	0.13	0.19	0.19	0.19	0.13	0.11	0.14	0.15	0.17	0.21	0.29	0.37	0.46	0.66		

(palatine shelf)																
Pterygoid	0.16	0.1	0.15	0.15	0.16	0.25	0.42	0.21	0.41	0.14	0.16	0.13	0.18	0.13	0.8	
Stapes	0.12	0.21	0.1	0.2	0.32	0.21	0.44	0.35	0.41	0.2	0.22	0.26	0.17	0.09	0.18	0.87

Table S5. Results of EMMLi analysis for trait correlations (ρ) within and between 16 cranial regions, for the phylogenetically-corrected dataset. Analyses were conducted on all 16 cranial regions.

	Frontal	Parietal	Nasopremaxilla (dorsal)	Maxillopalatine (lateral)	Os basale (occipital)	Occipital condyle	Quadrate (jaw joint)	Squamosal	Quadrate (lateral)	Os basale (ventral)	Nasopremaxilla (palatal)	Vomer	Maxillopalatine (interdental plate)	Maxillopalatine (palatine shelf)	Pterygoid	Stapes
Frontal	0.7															
Parietal	0.47	0.71														
Nasopremaxilla (dorsal)	0.25	0.13	0.49													
Maxillopalatine (lateral)	0.18	0.19	0.19	0.55												
Os basale (occipital)	0.15	0.39	0.1	0.2	0.71											
Occipital condyle	0.12	0.37	0.11	0.21	0.73	0.95										
Quadrate (jaw joint)	0.37	0.48	0.14	0.28	0.56	0.64	0.98									
Squamosal	0.2	0.26	0.14	0.29	0.27	0.36	0.56	0.69								
Quadrate (lateral)	0.31	0.4	0.15	0.25	0.43	0.51	0.85	0.53	0.81							
Os basale (ventral)	0.12	0.11	0.07	0.11	0.22	0.25	0.2	0.13	0.17	0.5						
Nasopremaxilla (palatal)	0.16	0.14	0.25	0.18	0.1	0.07	0.08	0.13	0.1	0.09	0.79					

Vomer	0.12	0.1	0.13	0.13	0.09	0.1	0.26	0.25	0.23	0.3	0.46	0.77				
Maxillopalatine (interdental plate)	0.19	0.21	0.2	0.4	0.19	0.16	0.28	0.27	0.26	0.14	0.36	0.22	0.83			
Maxillopalatine (palatine shelf)	0.13	0.12	0.15	0.28	0.19	0.19	0.2	0.2	0.2	0.2	0.25	0.33	0.55	0.71		
Pterygoid	0.12	0.16	0.12	0.19	0.28	0.33	0.41	0.24	0.4	0.12	0.09	0.11	0.16	0.17	0.78	
Stapes	0.21	0.26	0.13	0.16	0.35	0.35	0.55	0.27	0.47	0.15	0.23	0.25	0.19	0.14	0.22	0.87

Table S6. Covariance Ratio results for the full landmark and semilandmark dataset between each of the 16 cranial regions.

	Frontal	Parietal	Nasopremaxilla (dorsal)	Maxillopalatine (lateral)	Os basale (occipital)	Occipital condyle	Quadrate (jaw joint)	Squamosal	Quadrate (lateral)	Os basale (ventral)	Nasopremaxilla (palatal)	Vomer	Maxillopalatine (interdental plate)	Maxillopalatine (palatine shelf)	Pterygoid
Parietal	0.72														
Nasopremaxilla (dorsal)	0.71	0.54													
Maxillopalatine (lateral)	0.67	0.71	0.72												
Os basale (occipital)	0.48	0.59	0.44	0.64											
Occipital condyle	0.34	0.49	0.37	0.59	0.94										
Quadrate (jaw joint)	0.56	0.69	0.44	0.52	0.71	0.69									
Squamosal	0.69	0.84	0.64	0.76	0.61	0.56	0.71								
Quadrate (lateral)	0.62	0.73	0.56	0.6	0.7	0.66	0.97	0.8							
Os basale (ventral)	0.58	0.45	0.49	0.59	0.67	0.63	0.35	0.56	0.46						
Nasopremaxilla (palatal)	0.64	0.65	0.76	0.58	0.45	0.28	0.32	0.65	0.47	0.45					
Vomer	0.62	0.63	0.54	0.51	0.42	0.31	0.35	0.64	0.46	0.79	0.73				
Maxillopalatine (interdental plate)	0.59	0.57	0.69	0.74	0.7	0.62	0.46	0.65	0.57	0.6	0.7	0.45			
Maxillopalatine (palatine shelf)	0.54	0.59	0.62	0.68	0.7	0.61	0.5	0.64	0.6	0.68	0.57	0.62	0.8		
Pterygoid	0.45	0.33	0.53	0.43	0.49	0.44	0.52	0.49	0.59	0.44	0.43	0.37	0.48	0.42	
Stapes	0.64	0.79	0.57	0.63	0.67	0.57	0.81	0.77	0.84	0.45	0.61	0.47	0.55	0.53	0.53

Table S7. Covariance Ratio results using only anatomical landmarks for 14 cranial regions. The stapes and pterygoid regions are excluded from this analysis as they are only represented by semilandmarks.

	Frontal	Parietal	Nasopremaxilla (dorsal)	Maxillopalatine (lateral)	Os basale (occipital)	Occipital condyle	Quadrate (jaw joint)	Squamosal	Quadrate (lateral)	Os basale (ventral)	Nasopremaxilla (palatal)	Vomer	Maxillopalatine (interdental plate)
Parietal	1.40												
Nasopremaxilla (dorsal)	1.16	0.80											
Maxillopalatine (lateral)	1.14	1.07	1.09										
Os basale (occipital)	0.70	0.94	0.66	0.92									
Occipital condyle	0.54	0.80	0.54	0.88	1.15								
Quadrate (jaw joint)	0.78	0.89	0.64	0.85	0.89	0.82							
Squamosal	0.99	0.96	0.95	1.09	0.81	0.73	0.87						
Quadrate (lateral)	1.03	1.00	1.05	1.09	0.98	0.93	1.10	1.22					
Os basale (ventral)	0.96	0.96	0.72	0.91	0.89	1.04	0.51	0.81	0.89				
Nasopremaxilla (palatal)	1.04	0.87	1.20	1.01	0.60	0.40	0.52	0.95	1.02	0.60			
Vomer	0.94	0.96	0.79	0.74	0.61	0.49	0.42	0.85	0.77	0.93	1.12		
Maxillopalatine (interdental plate)	0.97	0.92	1.06	1.17	0.85	0.77	0.62	0.93	1.01	0.75	1.18	0.75	
Maxillopalatine (palatine shelf)	0.85	0.93	0.82	0.94	0.89	0.82	0.68	0.83	1.06	0.81	0.87	0.94	0.96

Table S8. Covariance Ratio results for the full landmark and semilandmark dataset, corrected for allometry.

	Frontal	Parietal	Nasopremaxilla (dorsal)	Maxillopalatine (lateral)	Os basale (occipital)	Occipital condyle	Quadrate (jaw joint)	Squamosal	Quadrate (lateral)	Os basale (ventral)	Nasopremaxilla (palatal)	Vomer	Maxillopalatine (interdental plate)	Maxillopalatine (palatine shelf)	Pterygoid
Parietal	0.75														
Nasopremaxilla (dorsal)	0.72	0.58													
Maxillopalatine (lateral)	0.71	0.72	0.75												
Os basale (occipital)	0.60	0.61	0.54	0.58											
Occipital condyle	0.46	0.41	0.45	0.45	0.85										
Quadrate (jaw joint)	0.61	0.62	0.47	0.43	0.58	0.56									
Squamosal	0.71	0.87	0.65	0.76	0.66	0.52	0.68								
Quadrate (lateral)	0.67	0.71	0.59	0.55	0.63	0.56	0.96	0.78							
Os basale (ventral)	0.62	0.57	0.51	0.57	0.64	0.56	0.35	0.60	0.49						
Nasopremaxilla (palatal)	0.61	0.63	0.76	0.60	0.57	0.39	0.36	0.68	0.51	0.49					
Vomer	0.60	0.67	0.53	0.53	0.50	0.37	0.42	0.66	0.51	0.82	0.71				
Maxillopalatine (interdental plate)	0.62	0.60	0.73	0.70	0.58	0.42	0.37	0.66	0.55	0.52	0.74	0.43			
Maxillopalatine (palatine shelf)	0.58	0.67	0.67	0.65	0.63	0.43	0.43	0.68	0.61	0.66	0.60	0.64	0.72		
Pterygoid	0.46	0.34	0.54	0.45	0.50	0.43	0.53	0.51	0.58	0.47	0.46	0.38	0.50	0.47	
Stapes	0.67	0.75	0.60	0.63	0.70	0.51	0.75	0.76	0.80	0.51	0.63	0.50	0.58	0.58	0.56

Table S9. Covariance Ratio results for the full landmark and semilandmark dataset, corrected for phylogeny.

	Frontal	Parietal	Nasopremaxilla (dorsal)	Maxillopalatine (lateral)	Os basale (occipital)	Occipital condyle	Quadrate (jaw joint)	Squamosal	Quadrate (lateral)	Os basale (ventral)	Nasopremaxilla (palatal)	Vomer	Maxillopalatine (interdental shelf)	Maxillopalatine (palatine shelf)	Pterygoid
Parietal	0.79														
Nasopremaxilla (dorsal)	0.68	0.58													
Maxillopalatine (lateral)	0.57	0.66	0.68												
Os basale (occipital)	0.50	0.73	0.56	0.75											
Occipital condyle	0.37	0.62	0.49	0.69	0.95										
Quadrate (jaw joint)	0.73	0.83	0.52	0.58	0.82	0.75									
Squamosal	0.72	0.86	0.62	0.69	0.79	0.75	0.85								
Quadrate (lateral)	0.75	0.85	0.55	0.61	0.84	0.75	0.99	0.89							
Os basale (ventral)	0.58	0.53	0.50	0.61	0.68	0.66	0.54	0.64	0.57						
Nasopremaxilla (palatal)	0.60	0.60	0.75	0.54	0.50	0.37	0.41	0.55	0.44	0.43					
Vomer	0.59	0.64	0.55	0.48	0.43	0.36	0.48	0.60	0.51	0.69	0.72				
Maxillopalatine (interdental shelf)	0.55	0.67	0.71	0.78	0.80	0.75	0.62	0.70	0.65	0.63	0.62	0.48			
Maxillopalatine (palatine shelf)	0.50	0.64	0.65	0.69	0.77	0.74	0.63	0.68	0.65	0.66	0.55	0.56	0.84		
Pterygoid	0.45	0.48	0.50	0.52	0.61	0.57	0.57	0.58	0.61	0.56	0.35	0.48	0.54	0.56	
Stapes	0.75	0.84	0.59	0.61	0.77	0.68	0.90	0.81	0.89	0.52	0.58	0.52	0.62	0.56	0.51

Table S10. Results from EMMLi analysis for trait correlations (ρ) within and between the ten identified cranial modules, using phylogenetically-corrected data.

	Frontal	Parietal	Nasopremaxilla (dorsal)	Maxillopalatine	Occipital	Quadrate-Squamosal	Ventral os basale-vomer	Nasopremaxilla (palatal)	Pterygoid	Stapes
Frontal	0.7									
Parietal	0.47	0.71								
Nasopremaxilla (dorsal)	0.25	0.13	0.49							
Maxillopalatine	0.17	0.17	0.18	0.5						
Occipital	0.15	0.39	0.1	0.2	0.73					
Quadrate-Squamosal	0.27	0.35	0.14	0.25	0.41	0.71				
Ventral os basale-vomer	0.12	0.11	0.09	0.17	0.18	0.18	0.46			
Nasopremaxilla (palatal)	0.16	0.14	0.25	0.25	0.09	0.11	0.21	0.79		
Pterygoid	0.12	0.16	0.12	0.18	0.29	0.33	0.12	0.09	0.78	
Stapes	0.21	0.26	0.13	0.16	0.35	0.4	0.18	0.23	0.22	0.87

Table S11. Covariance Ratio results between each of the ten identified cranial modules, using phylogenetically corrected data.

	Frontal	Parietal	Nasopremaxilla (dorsal)	Maxillopalatine	Occipital	Quadrate-squamosal	Ventral os basale-vomer	Nasopremaxilla (palatal)	Pterygoid
Parietal	0.79								
Nasopremaxilla (dorsal)	0.68	0.58							
Maxillopalatine	0.59	0.71	0.74						
Occipital	0.48	0.72	0.55	0.82					
Quadrate-squamosal	0.75	0.86	0.57	0.70	0.83				
Ventral os basale-vomer	0.63	0.62	0.56	0.67	0.64	0.61			
Nasopremaxilla (palatal)	0.60	0.60	0.75	0.60	0.48	0.47	0.61		
Pterygoid	0.45	0.48	0.50	0.58	0.61	0.60	0.57	0.35	
Stapes	0.74	0.84	0.59	0.64	0.76	0.90	0.56	0.58	0.51

Table S12. Summary of the PC axes for the full landmark and semilandmark dataset. 29 (indicated in bold) of the 34 PC axes were required to explain 99% of cranial shape variation.

	**PC1**	**PC2**	**PC3**	**PC4**	**PC5**	**PC6**	**PC7**	**PC8**	**PC9**	**PC10**
Standard deviation	0.05224	0.04406	0.03181	0.03047	0.02475	0.02248	0.01988	0.01817	0.01757	0.01533
Proportion of Variance	0.25671	0.18265	0.09521	0.08737	0.05765	0.04755	0.03717	0.03107	0.02903	0.0221
Cumulative Proportion	0.25671	0.43936	0.53457	0.62194	0.67959	0.72713	0.7643	0.79537	0.82439	0.84649
	**PC11**	**PC12**	**PC13**	**PC14**	**PC15**	**PC16**	**PC17**	**PC18**	**PC19**	**PC20**
Standard deviation	0.0144	0.01245	0.01183	0.01171	0.01078	0.009748	0.009577	0.008971	0.008639	0.007665
Proportion of Variance	0.0195	0.01458	0.01316	0.0129	0.01093	0.00894	0.00863	0.00757	0.00702	0.00553
Cumulative Proportion	0.866	0.88057	0.89374	0.90664	0.91757	0.92651	0.93514	0.94272	0.94974	0.95526
	**PC21**	**PC22**	**PC23**	**PC24**	**PC25**	**PC26**	**PC27**	**PC28**	**PC29**	PC30
Standard deviation	0.007387	0.007118	0.007054	0.00661	0.006593	0.006075	0.005835	0.005472	0.005267	0.004982
Proportion of Variance	0.00513	0.00477	0.00468	0.00411	0.00409	0.00347	0.0032	0.00282	0.00261	0.00234
Cumulative Proportion	0.9604	0.96516	0.96984	0.97395	0.97804	0.98152	0.98472	0.98754	0.99015	0.99248
	PC31	PC32	PC33	PC34						
Standard deviation	0.004845	0.004573	0.004475	0.003938						
Proportion of Variance	0.00221	0.00197	0.00188	0.00146						
Cumulative Proportion	0.99469	0.99666	0.99854	1						

Table S13. Centroid size of all specimens.

**Species**	**Centroid size**
Atretochoana eiselti	429.83
Boulengerula boulengeri	79.72
Brasilotyphlus braziliensis	115.38
Caecilia tentaculata	284.91
Ichthyophis nigroflavus	255.15
Chikila fulleri	121.96
Chthonerpeton indistinctum	237.84
Crotaphatrema bornmuelleri	156.99
Crotaphatrema lamottei	136.73
Dermophis mexicanus	299.74
Epicrionops bicolor	197.55
Gegeneophis carnosus	75.15
Geotrypetes seraphini	146.92
Grandisonia alternans	178.54
Gymnopis multiplicata	309.86
Herpele squalostoma	192.55
Hypogeophis rostratus	124.27
Ichthyophis bombayensis	252.80
Ichthyophis glutinosus	246.50
Idiocranium russeli	75.01
Indotyphlus cf. *battersbyi*	86.15
Luetkenotyphlus brasiliensis	103.13
Microcaecilia albiceps	111.87
Mimosiphonops vermiculatus	142.57
Nectocaecilia petersii	251.04
Oscaecilia ochrocephala	126.26

Potomotyphlus kaupii	161.32
Praslinia cooperi	157.78
Rhinatrema bivittatum	170.79
Schistometopum gregorii	163.21
Scolecomorphus kirkii	148.90
Siphonops annulatus	282.88
Sylvacaecilia grandisonae	209.00
Typhlonectes compressicauda	224.47
Uraeotyphlus oxyurus	152.94

Table S14. Differences ($x10^{-6}$) in the observed means of disparity among the ten cranial modules. A negative value indicates the module in the column has a higher disparity than the module in the row. Test performed using the 'TukeyHSD' function in R. * p ≤0.05, **p ≤0.01, ***p≤0.001, ****p≤0.0000001.

	Frontal	Parietal	Nasopremaxilla (dorsal)	Maxillopalatine	Occipital	Quadrate-Squamosal	Ventral os basale -vomer	Nasopremaxilla (palatal)	Pterygoid
Parietal	2.18****								
Nasopremaxilla (dorsal)	-0.47	-2.64****							
Maxillopalatine	1.57***	-0.61	2.04****						
Occipital	-1.66***	-3.84****	-1.19**	-3.23****					
Quadrate-Squamosal	4.04****	1.86****	4.51****	2.47****	5.70****				
Ventral os basale-vomer	-1.39***	-3.57****	-0.92*	-2.96****	0.27	-5.43****			
Nasopremaxilla (palatal)	-0.83	-3.01****	-0.36	-2.40****	0.83	-4.87****	0.56		
Pterygoid	4.40****	2.22***	4.87****	2.83****	6.06****	0.36	5.79****	5.23****	
Stapes	-1.60	-3.78***	-1.13	-3.17***	0.06	-5.64****	-0.21	-0.77	-6.00****

Table S15. Significance (p) values for differences in rates of morphological evolution among the ten cranial modules identified in this study. Results indicated in bold are significant ($p < 0.05$).

	Frontal	Parietal	Nasopremaxilla (dorsal)	Maxillopalatine	Occipital	Quadrate-Squamosal	Ventral os basale-vomer	Nasopremaxilla (palatal)	Pterygoid
Parietal	1								
Nasopremaxilla (dorsal)	1	1							
Maxillopalatine	1	1	0.093						
Occipital	1	1	0.738	0.146					
Quadrate-Squamosal	1	0.866	**0.001**	0.381	**0.005**				
Ventral os basale-vomer	1	0.191	0.933	0.524	0.822	**0.001**			
Nasopremaxilla (palatal)	1	**0.003**	1	0.999	1	**0.002**	0.989		
Pterygoid	0.243	1	0.999	1	0.998	0.999	1	1	
Stapes	1	1	1	1	1	1	1	1	1

Table S16. Ecological data for all species sampled in this study. For degree of fossoriality, the categories are: 0, aquatic species (which sometimes burrow in soft substrates); 1, tailed species (which have relatively short and stout bodies and zygokrotaphic skulls); 2, tailless species with zygokrotaphic skulls; 3, tailless species with stegokrotaphic skulls and open orbits; and 4, tailless species with closed orbits. All larval stage and reproductive mode information was taken from San Mauro *et al* 2014 [1] except for * species, whose data was based on nearest relatives. ** information is from personal observation (Mark Wilkinson). Data collected for *Indotyphlus cf. battersbyi* was based on *Indotyphlus battersbyi*.

**Species**	**Degree of fossoriality as adult (0,1,2,3,4)**	**Life history strategy (larval stage absent/present)**	**Reproductive mode**
Atretochoana eiselti	0	absent*	viviparous*
Boulengerula boulengeri	4	absent	oviparous
Brasilotyphlus braziliensis	4	absent*	oviparous*
Caecilia tentaculata	3	absent*	oviparous*
Chikila fulleri	4	absent	oviparous
Chthonerpeton indistinctum	2	absent	viviparous
Crotaphatrema bornmuelleri	4	absent*	NA
Crotaphatrema lamottei	4	absent*	NA
Dermophis mexicanus	3	absent	viviparous
Epicrionops bicolor	1	present*	oviparous*
Gegeneophis carnosus	4	absent*	oviparous*
Geotrypetes seraphini	2	absent	viviparous
Grandisonia alternans	3	NA	oviparous*
Gymnopis multiplicata	4	absent	viviparous
Herpele squalostoma	4	absent	oviparous
Hypogeophis rostratus	3	absent	oviparous
Ichthyophis bombayensis	1	present	oviparous
Ichthyophis glutinosus	1	present	oviparous
Ichthyophis nigroflavus	1	present*	oviparous*
Idiocranium russeli	3	absent	oviparous

Indotyphlus cf. *battersbyi*	3	absent*	oviparous*
Luetkenotyphlus brasiliensis	3	absent*	oviparous*
Microcaecilia albiceps	4	absent*	oviparous*
Mimosiphonops vermiculatus	3	absent*	oviparous*
Nectocaecilia petersii	2	absent**	viviparous**
Oscaecilia ochrocephala	4	absent*	oviparous*
Potomotyphlus kaupii	0	absent	viviparous
Praslinia cooperi	3	present	oviparous*
Rhinatrema bivitattum	1	present	oviparous
Schistometopum gregorii	3	absent	viviparous
Scolecomorphus kirkii	2	absent**	viviparous**
Siphonops annulatus	3	absent	oviparous
Sylvacaecilia grandisonae	3	present**	oviparous**
Typhlonectes compressicauda	0	absent**	viviparous**
Uraeotyphlus oxyurus	1	present	oviparous*

Table S17. Definitions for the 16 cranial regions analysed in this study, corresponding to coloured regions in Figure S1.

Region colour in Fig. S1	Region	Region description
Purple	Os basale (ventral)	The ventral surface of the os basale, separated from the occipital region by a curve tracing the posterior muscle ridge. This is analogous to the basisphenoid module previous identified in birds [2].
Pale pink	Frontal	Intraspecific analyses [3] have found the mesethmoid shape to correlate most strongly with the frontal, so the mesethmoid and frontal were patched as one region. The whole frontal and mesethmoid (down its midline).
Red	Quadrate (jaw joint)	The articular surface of the quadrate.
Grey	Maxillopalatine (interdental plate)	The ventral surface of the maxillopalatine immediately posterior to the primary tooth row.
Orange	Maxillopalatine (lateral)	The prefrontal was included in the lateral maxillopalatine region, because it has been shown to fuse with the maxillopalatine through development [4] and not with the frontal as was previously believed [5]. This region was the most difficult to landmark, because of its large variation in shape and housing of sensory openings. The maxillopalatine can house the orbit, the tentacular foramen, both or neither. The orbit, when present, is either housed completely within the maxillopalatine or sits between the maxillopalatine and squamosal. The tentacular foramen is either in the maxillopalatine or between the maxillopalatine and nasopremaxilla. The tentacle and orbit openings can be connected or separated. In addition, the tentacular opening can be a foramen (closed hole) or a fossa (a groove passing through the entire length of the maxillopalatine). This huge variation means defining homologous landmarks and curves is very difficult. Consequently, landmarks and curves were used as “fences” to constrain the regions for patching, but most were then removed before analyses. This way, we could use just two templates for patching these regions, one template with a defined hole (a negligibly sized hole was used for maxillopalatines with no hole) and one template that separated the maxillopalatine into regions dorsal and ventral to the tentacular fossa.
Hot	Maxillopalatine	The ventral surface posterior to the secondary tooth row is known as the palatine shelf (or maxillary plate) and

pink	(palatine shelf)	partially or completely borders the choana. When closed, the two landmarks defining the anterior and posterior border extremes of the choana were adjacently placed, reflecting the closing of the choana.
Green	Nasopremaxilla (dorsal)	The dorsal surface of the nasopremaxilla (or nasal and premaxilla), and septomaxilla when present.
Gold	Nasopremaxilla (palatal)	The ventral (palatal) surface of the nasopremaxilla posterior to the tooth row. The ventral surface consists of the naso/premaxilla in most specimens, but can be partially (e.g. *Geotrypetes seraphini*) or completely (*Scolecomorphus kirkii* and *Crotaphatrema lamottei*) covered by an anterior projection of the vomer. These bones were patched as one region, defined as the bone surface between the anterior first and second rows of teeth.
Aqua	Occipital condyle	The occipital condyle. The occipital condyle was separated from the occipital region by two curves defining its border.
Pale purple	Os basale (occipital)	Occipital region of the os basale, excluding occipital condyle. Ventrally, this region ended at the muscle ridge.
Black	Parietal	We are interested in the external bone surface, so when a parietal-squamosal fenestra was present, we excluded the parietal region covered by the adductor musculature in life. This region is covered in fasciae so is considered internal. The parietal region was therefore defined by the parietal's medial, anterior, and posterior margins, and the lateral margin was defined by the adductor ridge. For specimens *Rhinatrema bivittatum* and *Epicrionops bicolor,* which represent the extreme (primitive) condition of a large adductor muscle, this region is especially small.
Pale blue	Pterygoid	There is current debate surrounding bone homology for the pterygoid region of caecilians. There is a putative pterygoid (also referred to as the ectopterygoid) in some specimens, and all (except *Scolecomorphus kirkii* and *Crotaphatrema bornmuelleri*) have an anterior projection of the quadrate in ventral view, resembling the pterygoid in both shape and position. This is postulated to function as a pterygoid.<br>Given the variable presence of the pterygoid and the pterygoid process of the quadrate, we defined the pterygoid region as both bones. Separate patching across specimens was necessary, as this region variably includes only one of the bones, both, or neither. *Scolecomorphus kirkii* and *Crotaphatrema bornmuelleri*, which have neither bone, were given a negligibly sized pterygoid region on the ventral side of the quadrate.
Pale	Quadrate (lateral)	The lateral surface of the quadrate was defined by its anterior, lateral and medial borders, and the posterior edge

green		traced around the jaw joint and the stapes articulation, excluding both.
Dark blue	Squamosal	The postfrontal was present in only three specimens and was patched with the squamosal. The posterior orbit margin, when the orbit is not enclosed within the maxillopalatine, consists of the squamosal or the postfrontal. Although the depressor musculature attaches onto the squamosal, this region is not covered by fasciae so was considered external bone surface.
Yellow	Stapes	The stapes is present in all but three of the caecilian dataset (*Scolecomorphus kirkii*, *Crotaphatrema bornmuelleri* and *Crotaphatrema lamottei*). For all species with a stapes present, the lateral surface of the stapes was captured.
White	Vomer	The surface of the vomer posterior to the second row of teeth.

Table S18. Description of curves (consisting of sliding semilandmarks) placed onto each specimen. The manually-placed curve length refers to the amount of curve points placed in Landmark Editor, but, when exported from Landmark Editor, each curve is converted into eight or nine curve points. Following this, we subsampled the curve points to ensure all curve points were placed equidistantly along curves. These curve points were later slid to minimise bending energy across the specimens. Some curves (curves 58-75) were placed onto some specimens in order to aid the patching process but were deleted prior to analyses as they were not present across all specimens.

Original curves placed in Landmark Editor	Curves retained for analyses	Description	Bone	Region	Starting landmark	Ending landmark	Manually-placed curve length	Resampled curve length
SC1	SC1	Posteriorly, following midline of os basale	Os basale	Ventral surface	10	24	7	15
SC2	SC2	Laterally, following ridge	Os basale	Ventral surface	24	25	4	10
SC3	SC3	Anteriorly, following lateral outline of os basale	Os basale	Ventral surface	25	26	9	20
SC4	SC4	Anteriorly along anterior process of os basale	Os basale	Ventral surface	26	10	3	10
SC5	SC5	Posteriorly to foramen magnum	Os basale	Occipital	50	7	1	3
SC6	SC6	Following edge of foramen magnum	Os basale	Occipital	7	27	5	10
SC7	SC7	Following lateral edge of occipital condyle	Os basale	Occipital	27	28	5	10
SC8	SC8	Following foramen magnum	Os basale	Occipital	28	9	1	2

		medially to midline						
SC9	SC9	Ventrally to ridge	Os basale	Occipital	9	24	3	5
SC10	SC10	Follow outline of fenestra ovalis to maximum inflection point	Os basale	Occipital	25	29	3	10
SC11	SC11	Following the muscle ridge and not the bone margin (excluding the anterior process)	Os basale	Occipital	29	30	4	15
SC12	SC12	Following os basale-parietal suture to midline	Os basale	Occipital	30	50	6	15
SC13	SC13	Following anterior jaw joint outline	Quadrate	Jaw joint	11	12	3	6
SC14	SC14	Following posterior jaw joint outline	Quadrate	Jaw joint	12	54	2	3
SC15	SC15	Following posterior jaw joint outline	Quadrate	Jaw joint	54	11	2	3
SC16	SC16	Posteriorly, following medial edge of frontal	Frontal	Frontal	69	70	4	15
SC17	SC17	Laterally, following frontal-parietal suture	Frontal	Frontal	70	37	5	20
SC18	SC18	Anteriorly, along lateral frontal margin	Frontal	Frontal	37	5	8	10
SC19	SC19	Medially, following frontal-nasal suture	Frontal	Frontal	5	69	5	20

SC20	SC20	Posteriorly, following medial edge of parietal	Parietal	Parietal	38	6	8	20
SC21	SC21	Laterally, following parietal-os basale suture	Parietal	Parietal	6	40	6	15
SC22	SC22	Anteriorly, along lateral parietal margin	Parietal	Parietal	40	39	7	15
SC23	SC23	Medially, following parietal-frontal suture	Parietal	Parietal	39	38	4	10
SC24	SC24	Posteriorly, following the edge of the tooth row	Maxillopalatine	Interdental surface	14	15	8	20
SC25	SC25	Medially, along posterior edge	Maxillopalatine	Interdental surface	15	49	2	5
SC26	SC26	Anteriorly, in front of second tooth row	Maxillopalatine	Interdental surface	49	16	6	15
SC27	SC27	Laterally, following anterior edge	Maxillopalatine	Interdental surface	16	14	2	5
SC28	SC28	Laterally, following edge of the tooth row	Nasopremaxilla	Palatal surface	1	20	6	10
SC29	SC29	Following the edge of the naso/premaxilla posteriorly	Nasopremaxilla	Palatal surface	20	21	5	15
SC30	SC30	Anteriorly following the medial edge of the naso/premaxilla	Nasopremaxilla	Palatal surface	21	1	3	10
SC31	SC31	Laterally, following tooth row	Vomer	Vomer	22	23	4	10

SC32	SC32	Posteriorly, following edge of vomer	Vomer	Vomer	23	8	6	15
SC33	SC33	Anteriorly, following the medial edge of the vomer	Vomer	Vomer	8	22	5	15
SC34	SC34	Posteriorly, following the edge of the tooth row	Maxillopalatine	Palatine shelf	17	18	7	20
SC35	SC35	Following the bone margin	Maxillopalatine	Palatine shelf	18	19	5	15
SC36	SC36	Following the bone margin	Maxillopalatine	Palatine shelf	19	51	4	10
SC37	SC37	Following the margin of the choana	Maxillopalatine	Palatine shelf	51	52	8	20
SC38	SC38	Laterally, following the edge of the bone margin	Maxillopalatine	Palatine shelf	52	17	2	10
SC39	SC39	Ventrally, following the quadrate-squamosal suture	Quadrate	Lateral surface	53	48	7	15
SC40	SC40	Posteriorly, following the lateral edge of the quadrate	Quadrate	Lateral surface	48	12	4	5
SC41	SC41	Anteriorly, following the bone margin, tracing around where the stapes articulates with the quadrate	Quadrate	Lateral surface	11	53	6	15
SC42	SC42	Posteriorly along the medial margin of the squamosal	Squamosal	Squamosal	71	33	8	20
SC43	SC43	Ventrally, following the posterior margin of the squamosal	Squamosal	Squamosal	33	34	6	15

SC44	SC44	Anteriorly, following the lateral margin of the squamosal	Squamosal	Squamosal	34	32	5	10
SC45	SC45	Dorsally, following the anterior margin of the squamosal	Squamosal	Squamosal	32	71	7	20
SC46	SC46	Posteriorly, following the lateral edge of the maxillopalatine above the tooth row	Maxillopalatine	Lateral surface	41	42	7	30
SC47	SC47	Following the lateral edge of the maxillopalatine	Maxillopalatine	Lateral surface	42	2	2	5
SC48	SC48	Following the medial margin of the occipital condyle	Os basale	Occipital condyle	28	27	4	10
SC49	SC49	Posteriorly, following the medial margin of the nasopremaxilla	Nasopremaxilla	Dorsal surface	3	4	10	20
SC50	SC50	Laterally, following the nasopremaxilla-frontal suture	Nasopremaxilla	Dorsal surface	4	13	4	10
SC51	SC51	Ventrally, following the edge of the nasopremaxilla	Nasopremaxilla	Dorsal surface	13	43	7	20
SC52	SC52	Anteriorly, following the lateral edge of the nasopremaxilla, above the tooth row	Nasopremaxilla	Dorsal surface	43	3	5	10
SC53	SC53	Following the outline of the nares, from the dorsal to	Nasopremaxilla	Dorsal	45	44	3	5

		anterior extreme		surface				
SC54	SC54	Following the outline of the nares, from the anterior to ventral extreme	Nasopremaxilla	Dorsal surface	44	47	3	5
SC55	SC55	Following the outline of the nares, from the ventral to lateral extreme	Nasopremaxilla	Dorsal surface	47	46	3	5
SC56	SC56	Following the outline of the nares, from the lateral to dorsal extreme	Nasopremaxilla	Dorsal surface	46	45	3	5
SC57	SC57	Medially, following the ridge of the jaw joint	Quadrate	Jaw joint	12	11	5	5
SC58	*(removed)*	*Laterally, following the base of the process (excluding the "stem")*	*Quadrate*	*Pterygoid process*	*60*	*61*	*3*	*N/A*
SC59	*(removed)*	*Anteriorly, following the lateral margin*	*Quadrate*	*Pterygoid process*	*61*	*62*	*6*	*N/A*
SC60	*(removed)*	*Posteriorly, following the medial margin*	*Quadrate*	*Pterygoid process*	*62*	*60*	*5*	*N/A*
SC61	*(removed)*	*Dorsally, following the posterior margin of the maxillopalatine*	*Maxillopalatine*	*Lateral surface*	*2*	*63*	*9*	*N/A*
SC62	*(removed)*	*Anteriorly, following the medial margin of the maxillopalatine*	*Maxillopalatine*	*Lateral surface*	*63*	*64*	*5*	*N/A*
SC63	*(removed)*	*Ventrally, following the*	*Maxillopalatine*	*Lateral*	*64*	*2*	*9*	*N/A*

		anterior margin		*surface*				
SC61 (alternative)	*(removed)*	*Dorsally, following the posterior margin of the ventral section of maxillopalatine*	*Maxillopalatine*	*Lateral surface*	*2*	*63*	*9*	*N/A*
SC62 (alternative)	*(removed)*	*Anteriorly, following the medial margin of the ventral section of the maxillopalatine*	*Maxillopalatine*	*Lateral surface*	*63*	*64*	*5*	*N/A*
SC63 (alternative)	*(removed)*	*Ventrally, following the anterior margin of the ventral section of the maxillopalatine*	*Maxillopalatine*	*Lateral surface*	*64*	*2*	*9*	*N/A*
SC64	*(removed)*	*Following the base of the stapes, from posterior to dorsal extreme*	*Stapes*	*Stapes*	*56*	*58*	*2*	*N/A*
SC65	*(removed)*	*Following the base of the stapes, from dorsal to anterior extreme*	*Stapes*	*Stapes*	*58*	*57*	*2*	*N/A*
SC66	*(removed)*	*Following the base of the stapes, from anterior to ventral extreme*	*Stapes*	*Stapes*	*57*	*59*	*2*	*N/A*
SC67	*(removed)*	*Following the base of the stapes, from ventral to posterior extreme*	*Stapes*	*Stapes*	*59*	*56*	*2*	*N/A*
SC68	*(removed)*	*Following the main axes of the stapes rod, taking the most direct, lateral route whilst following any natural*	*Stapes*	*Stapes*	*56*	*55*	*4*	*N/A*

		curve						
SC69	*(removed)*	*Following the outline of the orbit/tentacular foramen, from posterodorsal to anterodorsal extreme*	*Maxillopalatine*	*Lateral surface*	*65*	*66*	*5*	*N/A*
SC70	*(removed)*	*Following the outline of the orbit/tentacular foramen, from anterodorsal to anteroventral extreme*	*Maxillopalatine*	*Lateral surface*	*66*	*68*	*5*	*N/A*
SC71	*(removed)*	*Following the outline of the orbit/tentacular foramen, from anteroventral to posteroventral extreme*	*Maxillopalatine*	*Lateral surface*	*68*	*67*	*5*	*N/A*
SC72	*(removed)*	*Following the outline of the orbit/tentacular foramen, from posteroventral to posterodorsal extreme*	*Maxillopalatine*	*Lateral surface*	*67*	*65*	*5*	*N/A*
SC69 (alternative)	*(removed)*	*Anteriorly, following the edge of the dorsal section of the maxillopalatine*	*Maxillopalatine*	*Lateral surface*	*65*	*66*	*5*	*N/A*
SC70 (alternative)	*(removed)*	*Posteriorly, following the edge of the dorsal section of the maxillopalatine*	*Maxillopalatine*	*Lateral surface*	*66*	*68*	*5*	*N/A*
SC71 (alternative)	*(removed)*	*Posteriorly, following the edge of the dorsal section of the maxillopalatine*	*Maxillopalatine*	*Lateral surface*	*68*	*67*	*5*	*N/A*
SC72	*(removed)*	*Anteriorly, following the edge of the dorsal section of the*	*Maxillopalatine*	*Lateral*	*67*	*65*	*5*	*N/A*

(alternative)		*maxillopalatine*		*surface*				
SC73	*(removed)*	*Laterally along posterior margin of the pterygoid*	*Pterygoid*	*Pterygoid*	*72*	*73*	*2*	*N/A*
SC74	*(removed)*	*Anteriorly along lateral margin of the pterygoid*	*Pterygoid*	*Pterygoid*	*73*	*74*	*6*	*N/A*
SC75	*(removed)*	*Posteriorly along medial margin of the pterygoid*	*Pterygoid*	*Pterygoid*	*74*	*72*	*5*	*N/A*

Table S19. Number of surface points projected onto each of the cranial regions.

**Cranial region**	**Number of surface points**
Frontal	56
Parietal	63
Nasopremaxilla (dorsal)	60
Maxillopalatine (lateral)	96
Os basale (occipital)	88
Occipital condyle	12
Quadrate (jaw joint)	30
Squamosal	35
Quadrate (lateral)	20
Os basale (ventral)	96
Nasopremaxilla (palatal)	21
Vomer	26
Maxillopalatine (interdental plate)	26
Maxillopalatine (palatine shelf)	30
Pterygoid	50
Stapes	20
**TOTAL**	**729**

Table S20. Alternative models of modular organisation tested in EMMLi analysis: 2A (1- not cheek, 2- cheek), 2B (1- anterior, 2-posterior), 2C (1-dorsal, 2-ventral), 2D (1-snout&cheek, 2-braincase), 2E (1-snout, 2-cheek&braincase), 3 (1-snout, 2-braincase, 3-cheek), 6A (approx. analogous to 1-vault, 2-anterior oral-nasal, 3-orbit, 4-basicranium, 5-zygomatic-pterygoid, 6-molar, see Goswami 2006), 6B (1-vault, 2-facial, 3-occipital, 4-cheek, 5-sphenoid, 6-palate). For 8 modules and higher, different groupings of the 16 cranial regions were tested.

**Region**	**No modules**	**2A**	**2B**	**2C**	**2D**	**2E**	**3**	**6A**	**6B**	**8**	**9A**	**9B**	**12**	**15**	**16**
Frontal	1	1	1	1	1	1	1	1	1	1	1	1	1	1	1
Parietal	1	1	2	1	2	2	2	1	1	1	1	1	2	2	2
Nasopremaxilla (dorsal)	1	1	1	1	1	1	1	2	2	2	2	2	3	3	3
Maxillopalatine (lateral)	1	2	1	1	1	1	1	3	2	3	2	3	4	4	4
Os basale (occipital)	1	1	2	1	2	2	2	4	3	4	3	4	5	5	5
Occipital condyle	1	1	2	1	2	2	2	4	3	4	3	4	5	6	6
Quadrate (jaw joint)	1	2	2	2	1	2	3	5	4	5	4	5	6	7	7
Squamosal	1	2	2	1	1	2	3	5	4	5	5	5	6	8	8
Quadrate (lateral)	1	2	2	1	1	2	3	5	4	5	5	5	6	7	9
Os basale (ventral)	1	1	2	2	2	2	2	4	5	5	6	6	7	9	10
Nasopremaxilla (palatal)	1	1	1	2	1	1	1	2	6	6	7	7	8	10	11
Vomer	1	1	1	2	1	1	1	2	6	6	7	7	9	11	12
Maxillopalatine (interdental plate)	1	2	1	2	1	1	1	6	6	6	7	7	10	12	13
Maxillopalatine (palatine shelf)	1	2	1	2	1	1	1	6	6	6	7	7	10	13	14
Pterygoid	1	2	2	2	1	2	3	5	4	7	8	8	11	14	15
Stapes	1	1	2	1	2	2	2	5	4	8	9	9	12	15	16

References for Additional File 1:

1. San Mauro D, Gower DJ, Müller H, Loader SP, Zardoya R, Nussbaum RA, et al. Life-history evolution and mitogenomic phylogeny of caecilian amphibians. Mol Phylogenet Evol. 2014;73:177–89.

2. Felice RN, Goswami A. Developmental origins of mosaic evolution in the avian cranium. Proc Natl Acad Sci U S A. 2018;115:555–60.

3. Marshall AF, Bardua C, Gower DJ, Wilkinson M, Sherratt E, Goswami A. Assessing variational modularity in caecilian (Amphibia: Gymnophiona) crania using a dense three-dimensional geometric morphometric approach (*in press*).

4. Wake MH, Hanken J. Development of the skull of *Dermophis mexicanus* (Amphibia: Gymnophiona), with comments on skull kinesis and amphibian relationships. J Morphol. 1982;173:203–23.

5. Marcus H, Stimmelmayr E, Porsch G. Beitrage zur Kenntnis der Gymnophionen. XXV. Die Ossifikation des Hypogeophisschddels. Morphol Jahrb. 1935;76:375–420.
